# Supplementary material for: FEMA-Long: Modeling unstructured covariances for discovery of time-dependent effects in large-scale longitudinal datasets
Source: PLoS Genet. 2026 Jun 11;22(6):e1012184. doi: 10.1371/journal.pgen.1012184 (PMC13286282; doi:10.1371/journal.pgen.1012184)
Supplement: S1 Text — (DOCX) [file pgen.1012184.s001.docx]

FEMA-Long: Modeling unstructured covariances for discovery of
time-dependent effects in large-scale longitudinal datasets

--- Supplementary Text ---

**List of authors:**

Pravesh Parekh^1,2,*^, Nadine Parker^1^, Diliana Pecheva^2^, Evgeniia Frei^1^, Marc Vaudel^4,5,6^, Diana M. Smith^7,8^, Alison Rigby^9^, Piotr Jahołkowski^1^, Ida Elken Sønderby^1,10^, Viktoria Birkenæs^1,11^, Nora Refsum Bakken^1,11^, Chun Chieh Fan^12^, Carolina Makowski^13^, Jakub Kopal^1^, Robert Loughnan^2,14^, Donald J. Hagler Jr.^2^, Dennis van der Meer^1^, Stefan Johansson^4,15^, Pål Rasmus Njølstad^4,15^, Terry L. Jernigan^3,7,13,16^, Wesley K. Thompson^12^, Oleksandr Frei^1,17^, Alexey A. Shadrin^1^, Thomas E. Nichols^18,19^, Ole A. Andreassen^1^, Anders M. Dale^2,20,21*^

**Affiliations:**

**^1^**Centre for Precision Psychiatry, Division of Mental Health and Addiction, University of Oslo and Oslo University Hospital, Oslo, Norway.

**^2^**Center for Multimodal Imaging and Genetics, J. Craig Venter Institute, La Jolla, California, United States of America.

**^3^**Department of Radiology, School of Medicine, University of California San Diego, La Jolla, California, United States of America.

^4^Mohn Center for Diabetes Precision Medicine, Department of Clinical Science, University of Bergen, Bergen, Norway.

^5^Computational Biology Unit, Department of Informatics, University of Bergen, Bergen, Norway.

^6^Department of Genetics and Bioinformatics, Norwegian Institute of Public Health, Bergen, Norway.

^7^Center for Human Development, University of California San Diego, La Jolla, California, United States of America.

^8^Medical Scientist Training Program, University of California San Diego, La Jolla, California, United States of America.

^9^Neuroscience Graduate Program, University of California San Diego, La Jolla, California, United States of America.

^10^Department of Medical Genetics, Oslo University Hospital & University of Oslo, Oslo, Norway.

^11^PsychGen Center for Genetic Epidemiology and Mental Health, Norwegian Institute of Public Health, Oslo, Norway.

^12^Center for Population Neuroscience and Genetics, Laureate Institute for Brain Research, Tulsa, OK, United States of America.

^13^Department of Psychiatry, School of Medicine, University of California San Diego, La Jolla, California, United States of America.

^14^Population Neuroscience and Genetics Lab, University of California San Diego, La Jolla, California, United States of America.

^15^Department of Pediatrics and Adolescents, Haukeland University Hospital, Bergen, Norway.

^16^Department of Cognitive Science, University of California San Diego, La Jolla, California, United States of America.

^17^Department of Pharmacy, Section for Pharmacology and Pharmaceutical Biosciences, University of Oslo, Oslo, Norway.

^18^Big Data Institute, Li Ka Shing Centre for Health Information and Discovery, Nuffield Department of Population Health, University of Oxford, Oxford, United Kingdom.

^19^Oxford Centre for Integrative Neuroimaging, FMRIB, Nuffield Department of Clinical Neurosciences, University of Oxford, Oxford, UK.

^20^University of California San Diego, La Jolla, California, United States of America.

^21^University of Oslo, Oslo, Norway.

*Correspondence should be addressed to:

Dr. Pravesh Parekh: [praveshparekh@gmail.com](mailto:praveshparekh@gmail.com) (PP)

Prof. Anders M. Dale: [andersmdale@gmail.com](mailto:andersmdale@gmail.com) (AMD)

# Phenotype for GWAS

As mentioned in the main manuscript, the phenotype of interest was the length, weight, and BMI of infants at six time points during the first year of life: birth, length at six weeks, three months, six months, eight months, and twelve months. The starting sample size was data on 113,342 infants. The measurements were derived from a combination of Medical Birth Registry of Norway (MBRN) and questionnaires filled by the mothers six and eighteen months after birth. Within the questionnaire filled by mothers when their child was six months old, the questions included a record of the length and weight of the child at birth, six weeks, three months, and six months. For the latter three, the mother was asked to refer to the child’s health card. Within the questionnaire filled by mothers when their child was eighteen months old, the questions included the length and weight of the child at eight months, around one year, and between fifteen to eighteen months.

## Consolidating measurements

First, we ensured that we had information regarding birth year for every child. Additionally, we ensured that there were no duplicated records. Then, we removed the data on 214 individuals where the sex of the child was unknown. Next, we removed 646 individuals where the pregnancy duration was less than 22 weeks or more than 44 weeks and 7,343 individuals where the gestational period was less than 37 weeks. Next, using the MBRN records, we removed the data on 4,582 infants with congenital malformations; we additionally checked the records for chromosomal abnormalities and Down’s syndrome but at this point no such records were in the dataset.

Next, for each timepoint, we ensured that the age at that timepoint was greater than the mode of the age at its preceding timepoint and less than the mode of the age at the next timepoint. For example, age of infants at three months measurement ought to be greater than the mode of age at six weeks and less than the mode of age at six months. For the measurement at twelve months, we used the upper cut-off of 425 days. This resulted in removing the data of 1,127 individuals (six weeks), 1,009 individuals (three months), 1,019 individuals (six months), 2,223 individuals (eight months), and 1,286 individuals (twelve months). Next, for any individual where the birth length or weight was missing, we replaced the measurement with the questionnaire data; for 1,611 individuals, birth length or weight record was missing in both the MBRN and the questionnaire data – these entries were removed. Finally, we removed the data on fifteen individuals where the six-week age was less than seven days, and the weight gain (since birth) was more than 400 grams.

## Examining growth charts

We calculated sex-specific percentiles of length and weight measurements at each timepoint. Specifically, we removed any observation that was less than 0.5^th^ percentile or greater than 99.5^th^ percentile at each timepoint. After this step, we were left with the data on 98,946 individuals. Then, we examined growth curves and flagged data points as outliers using a strategy described in (1): for each successive pair of timepoints (birth and six weeks, six weeks and three months, and so forth), we computed the $\text{log}_{2}$ of the ratios (for both length and weight; for example, $\text{log}_{2}\left( \frac{3m}{6w} \right)$). Using this value, we computed the median and the 0.0013 and 0.9986 quantiles of the data (normal cumulative distribution function at values -3 and 3 respectively). Using these three values, we derived a signed ratio which identified whether the timepoint value was an outlier (see (1) for more details). We ran this growth curve inspection stage only once (rather than iteratively) and marked these outliers. At this point, we also calculated the BMI for the infants and merged the dataset with the subjects who had been genotyped and who’s genetic data had passed quality control (2). We removed the records of three individuals where there was a mismatch between genotyping sex and the information retrieved from MBRN/questionnaire. The steps till this point were carried out in Python 3.9.6 using libraries Pandas 2.2.3 (3), NumPy 2.0.2 (4), and SciPy 1.13.1 (5).

## Final quality check

At this point, we had data on 68,514 unique individuals and a total of 411,084 observations. Out of these, we removed 93,024 observations where the age was missing, followed by removing 16,524 observations where either length or weight information was missing. These 301,536 observations with full information were subjected to a few additional quality check steps. We identified 1,065 individuals where there was one or more duplicated age; out of these, there were 244 observations which had different length or weight values at the same age (which we removed). There were 66 observations where there was a discrepancy between the timepoint and the ordering of age (for example, age at eight months timepoint was smaller than the age at six months timepoint).

In the remaining dataset of 300,283 observations, we found 109 individuals where there was one instance of duplicated BMI value (i.e., observations with the same value of length and weight but different age value) – since it was not possible to ascertain which of these observations were artifactual, we removed 218 observations of duplicated BMI. Next, we checked for scenarios where the length of the individual decreased with age. We found 114 individuals where this was so; for these individuals, we removed 228 measurements where length had decreased. Finally, we removed the data for 85 monozygotic twin pairs (one of the twins). At the end of this process, we were left with 299,447 complete length, weight, and BMI observations on 68,273 infants between birth and first year of life. The final sample size for GWAS was 68,273 infants with 299,447 observations having complete data on length, weight, and BMI (i.e., each of the 68,273 subjects had all three measurements).

# Comparison between two-stage regression and full regression

When fitting GWAS-like models, we assume that the effect of each SNP is small and would not have an impact on the estimation of the random effects covariance components. To evaluate the impact of this assumption, for a selected subset of SNPs, we performed a comparison of parameter estimation from fitting a full model vs. when fitting a two-stage model. Concretely, from the time-varying GWAS analyses of length, weight, and BMI, we extracted top ten SNPs per chromosome, per phenotype. This resulted in a subset of 1,110 SNPs. For each of these SNPs $g$, we fit the full regression model (i.e., 1,110 separate models):

$$\begin{aligned} \text{Phenotype \textasciitilde}1+g\boldsymbol{+}g\boldsymbol{\odot}s\left( \text{age} \right)\boldsymbol{+}s\left( \text{age} \right)+\text{sex}+\text{PC}_{1-20}+\text{Batch}+ \\ \text{us}\left( 1 | \text{Family} \right)+\text{us}\left( 1 | \text{GRM} \right)+\text{us}\left( 1 | \text{Subject} \right) \#\left( \text{S}1 \right) \end{aligned}$$

Then, we compared the estimated model parameters from the full model against the estimated parameters when performing two-stage regression described in the main manuscript. The results (**S46-S51 Figs**) indicate that the two approaches yield comparable estimates.

# Assessing the impact of using nearestSPD

As mentioned in the main manuscript text, when calculating the standard errors for the fixed effects using the generalized least squares solution (GLS), we use a nearest symmetric positive semidefinite algorithm, based on (6) and implemented in (7). To assess the impact of using this on the standard errors, we performed the following simulation:

Let the condition number (the ratio of the largest singular value to the smallest) of a covariance matrix be $C$. Let $\lambda_{\text{min}}$ be the smallest singular value; then $\lambda_{\text{max}}=\lambda_{\text{min}}\times C$. We simulated the case of 50 $X$ variables for a range of $\lambda_{\text{min}}$ and $C$, with linearly spaced 50 singular values between $\lambda_{\text{min}}$ and $\lambda_{\text{max}}$. Then, using QR decomposition, we created a symmetric covariance matrix. Since the square root of the diagonal elements of this matrix is the standard error, we compared the standard errors before and after applying the nearestSPD (7). We generated eight log-spaced values of $C$ (between 0 and 7) and let $\lambda_{\text{min}}$vary between 0.1 and 1 with a spacing of 0.1. For each of the combination of $C$ and $\lambda_{\text{min}}$, we generated 10 covariance matrices and calculated the largest difference in the standard errors before and after converting the covariance matrices to be symmetric positive semidefinite.

The results of this simulation (see, **S52 Fig**) show that the largest difference in the standard errors were minuscule (ranging between $5.55e^{-17}$ and $1.82e^{-12}$), indicating that the nearestSPD algorithm did not make any substantial differences to the covariance matrix.

# Supplementary References

1. Helgeland Ø, Vaudel M, Juliusson PB, Lingaas Holmen O, Juodakis J, Bacelis J, et al. Genome-wide association study reveals dynamic role of genetic variation in infant and early childhood growth. Nat Commun. 2019 Oct 1;10(1):4448. doi:10.1038/s41467-019-12308-0

2. Corfield EC, Shadrin AA, Frei O, Rahman Z, Lin A, Athanasiu L, et al. The Norwegian Mother, Father, and Child cohort study (MoBa) genotyping data resource: MoBaPsychGen pipeline v.1 [Internet]. bioRxiv; 2024 [cited 2024 Jul 17]. p. 2022.06.23.496289. Available from: https://www.biorxiv.org/content/10.1101/2022.06.23.496289v4 doi:10.1101/2022.06.23.496289

3. The pandas development team. pandas-dev/pandas: Pandas [Internet]. Zenodo; 2020. Available from: https://doi.org/10.5281/zenodo.3509134 doi:10.5281/zenodo.3509134

4. Harris CR, Millman KJ, Walt SJ van der, Gommers R, Virtanen P, Cournapeau D, et al. Array programming with NumPy. Nature. 2020 Sep;585(7825):357–62. doi:10.1038/s41586-020-2649-2

5. Virtanen P, Gommers R, Oliphant TE, Haberland M, Reddy T, Cournapeau D, et al. SciPy 1.0: Fundamental Algorithms for Scientific Computing in Python. Nature Methods. 2020;17:261–72. doi:10.1038/s41592-019-0686-2

6. Higham NJ. Computing a nearest symmetric positive semidefinite matrix. Linear Algebra and its Applications. 1988;103:103–18. doi:https://doi.org/10.1016/0024-3795(88)90223-6

7. D’Errico J. nearestSPD [MATLAB] [Internet]. MATLAB Central File Exchange; [cited 2026 Mar 16]. Located at: MATLAB Central File Exchange. Available from: https://www.mathworks.com/matlabcentral/fileexchange/42885-nearestspd
