# Supplementary figures and images for: FEMA-Long: Modeling unstructured covariances for discovery of time-dependent effects in large-scale longitudinal datasets
Source: PLoS Genet. 2026 Jun 11;22(6):e1012184. doi: 10.1371/journal.pgen.1012184 (PMC13286282; doi:10.1371/journal.pgen.1012184)

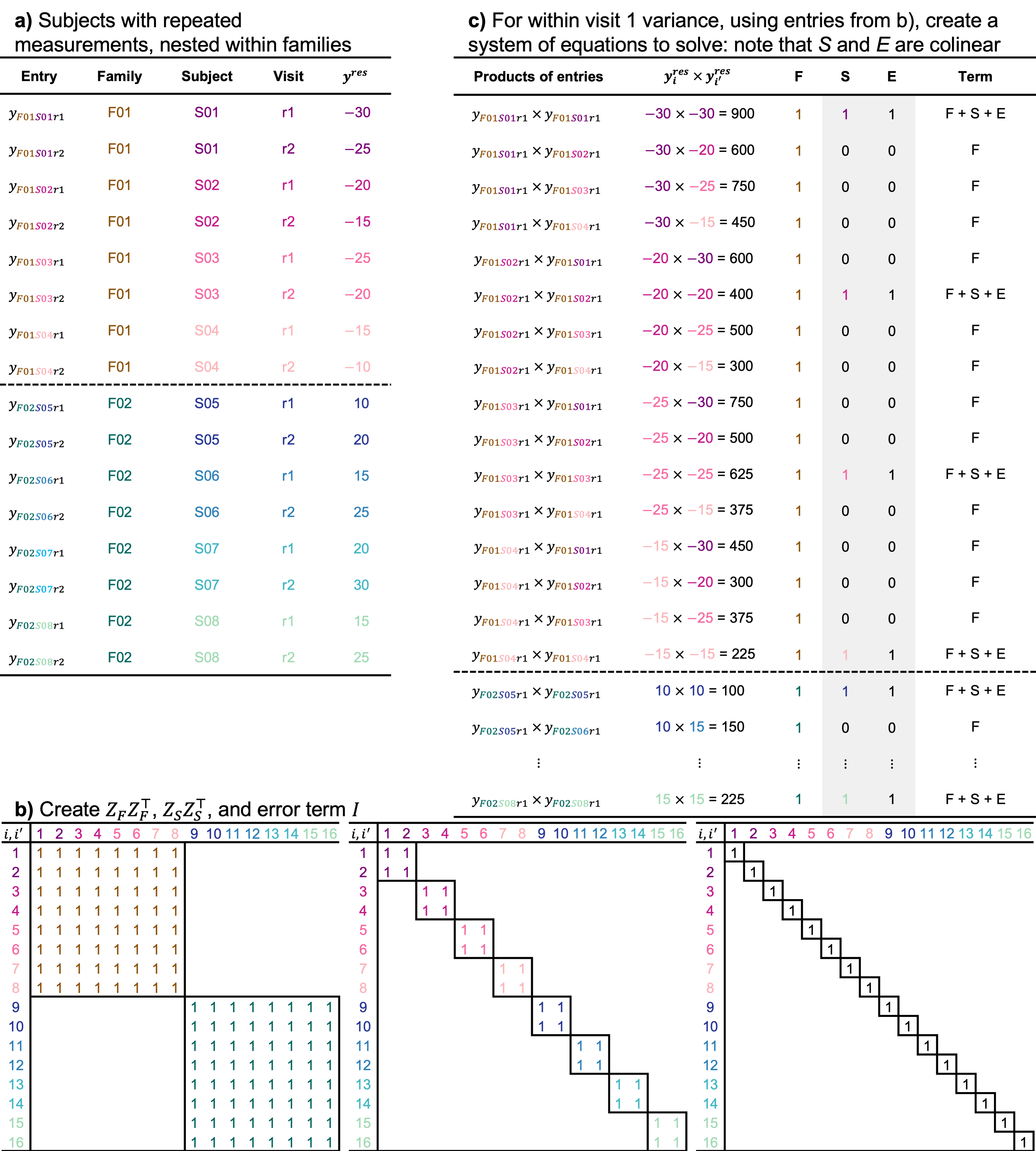

Supplement: S1 Fig — a) consider a dataset with eight subjects across two families, each having two repeated measurements; b) dummy-coding families ZF and subjects ZS, followed by creating the ZFZF⊤, ZSZS⊤ terms and the independent error term denoted by I; c) following equation 11 in the manuscript, create a system of equations to solve for visit 1: the terms S and E are colinear, and cannot be uniquely identified; therefore, these are estimated together. A similar set of equations can be constructed for visit 2. (TIFF) [file pgen.1012184.s013.tiff]

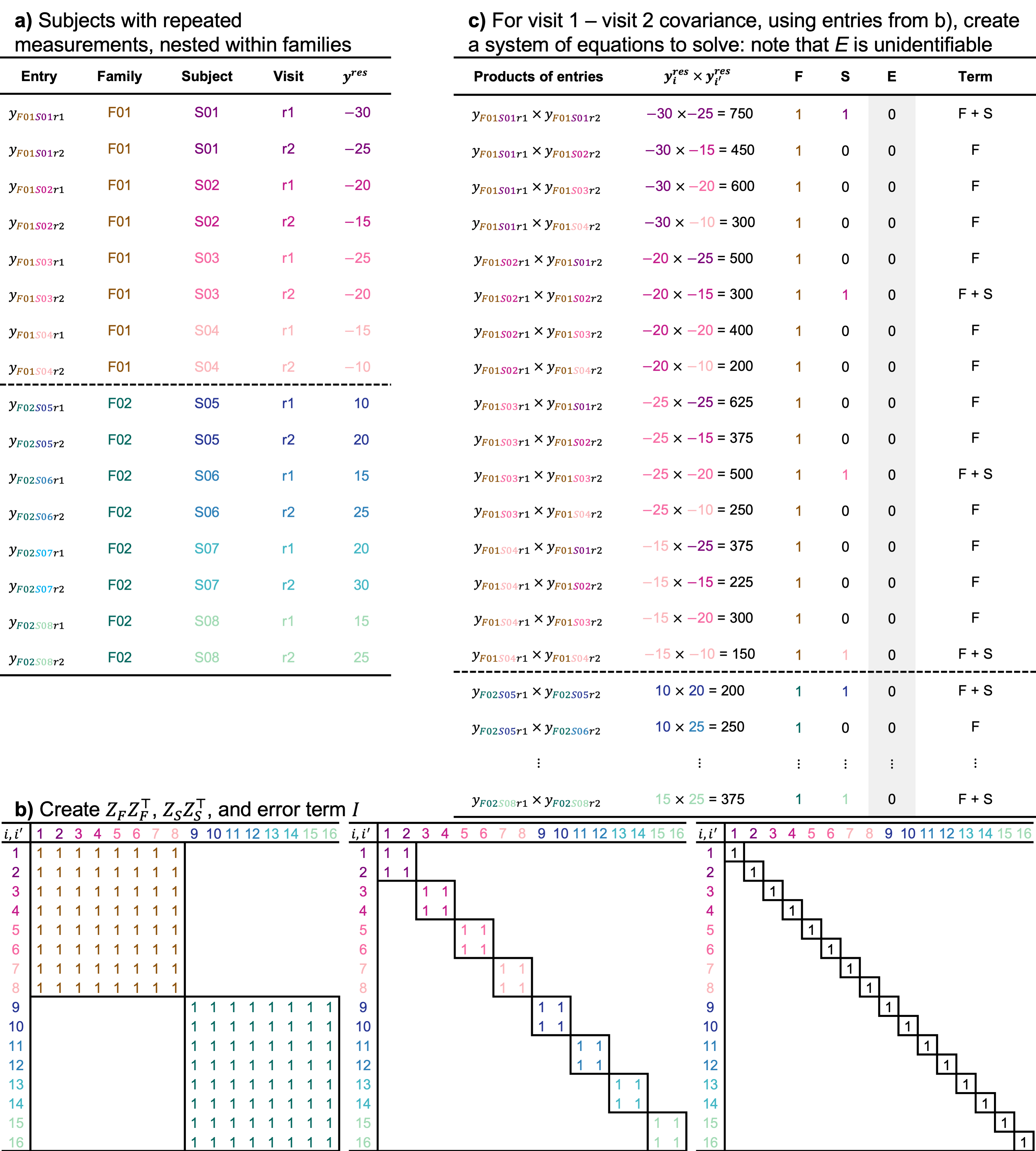

Supplement: S2 Fig — a) consider a dataset with eight subjects across two families, each having two repeated measurements; b) dummy-coding families ZF and subjects ZS, followed by creating the ZFZF⊤, ZSZS⊤ terms and the independent error term denoted by I; c) following equation 11 in the manuscript, create a system of equations to solve for visit 1 – visit 2: the term E becomes unidentifiable in this case. (TIFF) [file pgen.1012184.s014.tiff]

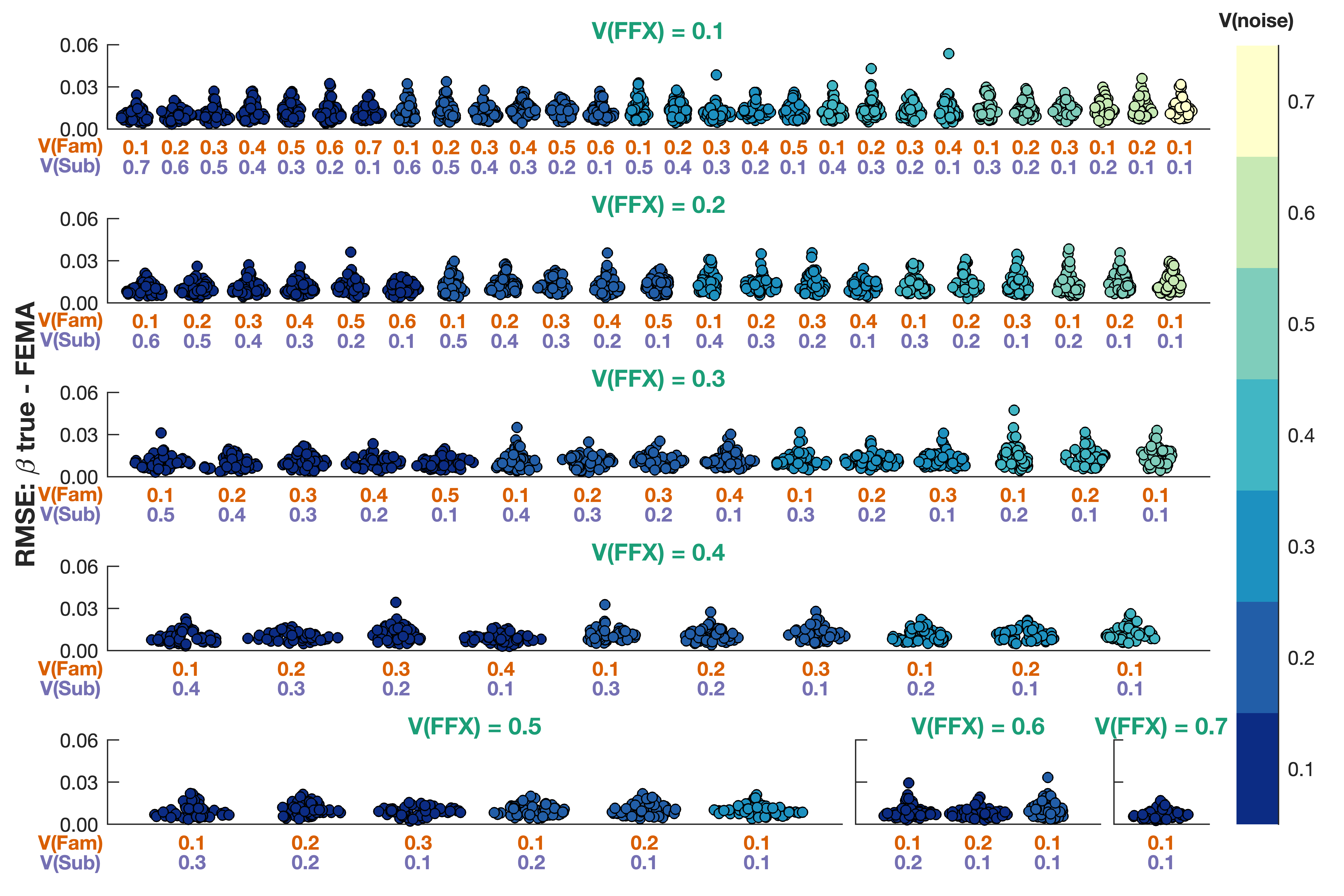

Supplement: S3 Fig — Each panel shows a simulation condition with the amount of variance in the phenotypes explained by the fixed effect V(FFX) shown on the top and the amounts of variances explained by family and subject effects V(Fam) and V(Sub) labeled on the x-axis. Each point shows the root mean squared error (RMSE) between the simulated ground truth and the estimates from FEMA, repeated 50 times for each simulation scenario, color-coded by the amount of noise in the phenotype. (TIFF) [file pgen.1012184.s015.tiff]

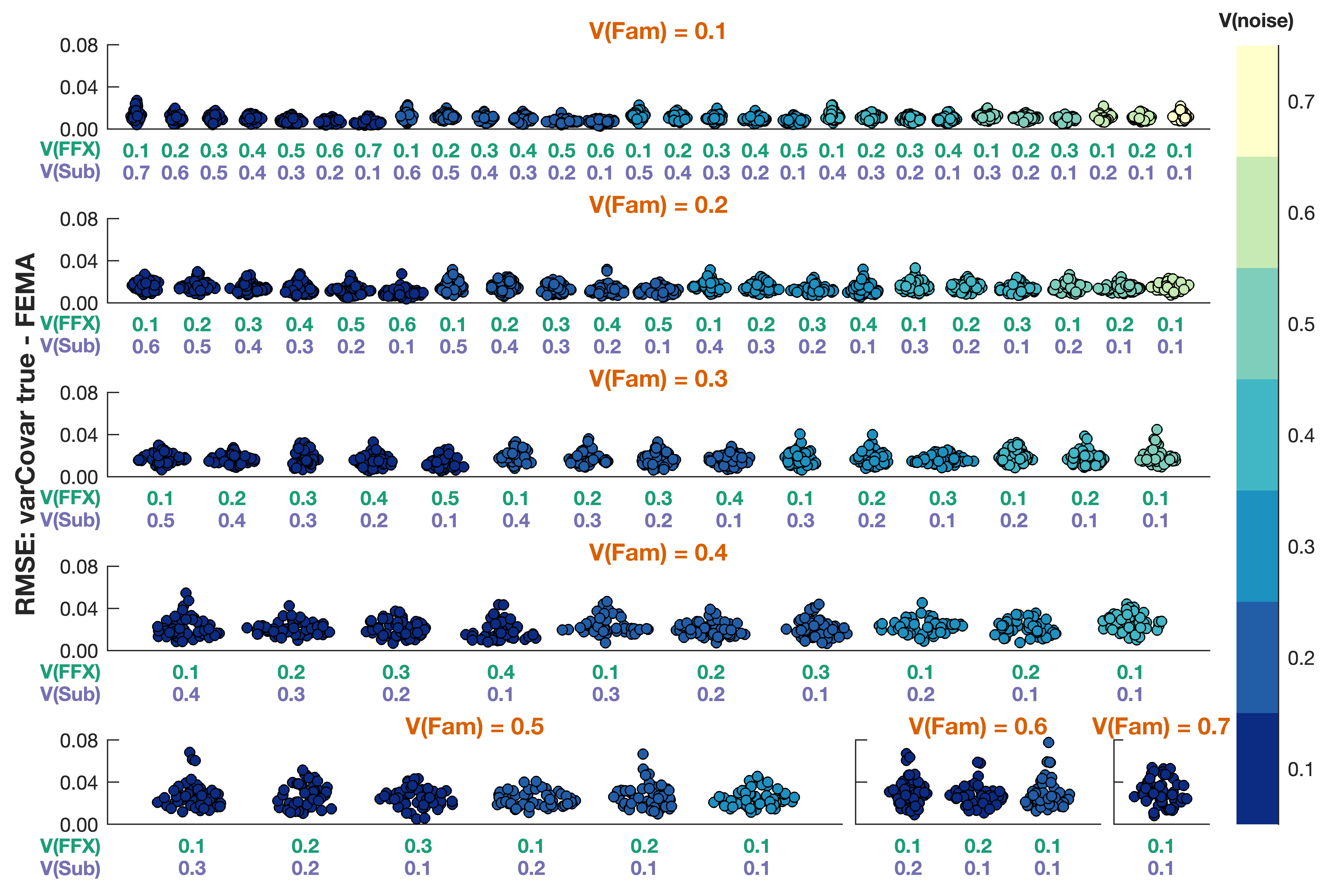

Supplement: S4 Fig — Each panel shows a simulation condition with the amount of variance in the phenotypes explained by the family effect V(Fam) shown on the top and the amounts of variances explained by fixed effects and subject effects V(FFX) and V(Sub) labeled on the x-axis. Each point shows the root mean squared error (RMSE) between the simulated ground truth and the estimates from FEMA, repeated 50 times for each simulation scenario, color-coded by the amount of noise in the phenotype. (TIFF) [file pgen.1012184.s016.tiff]

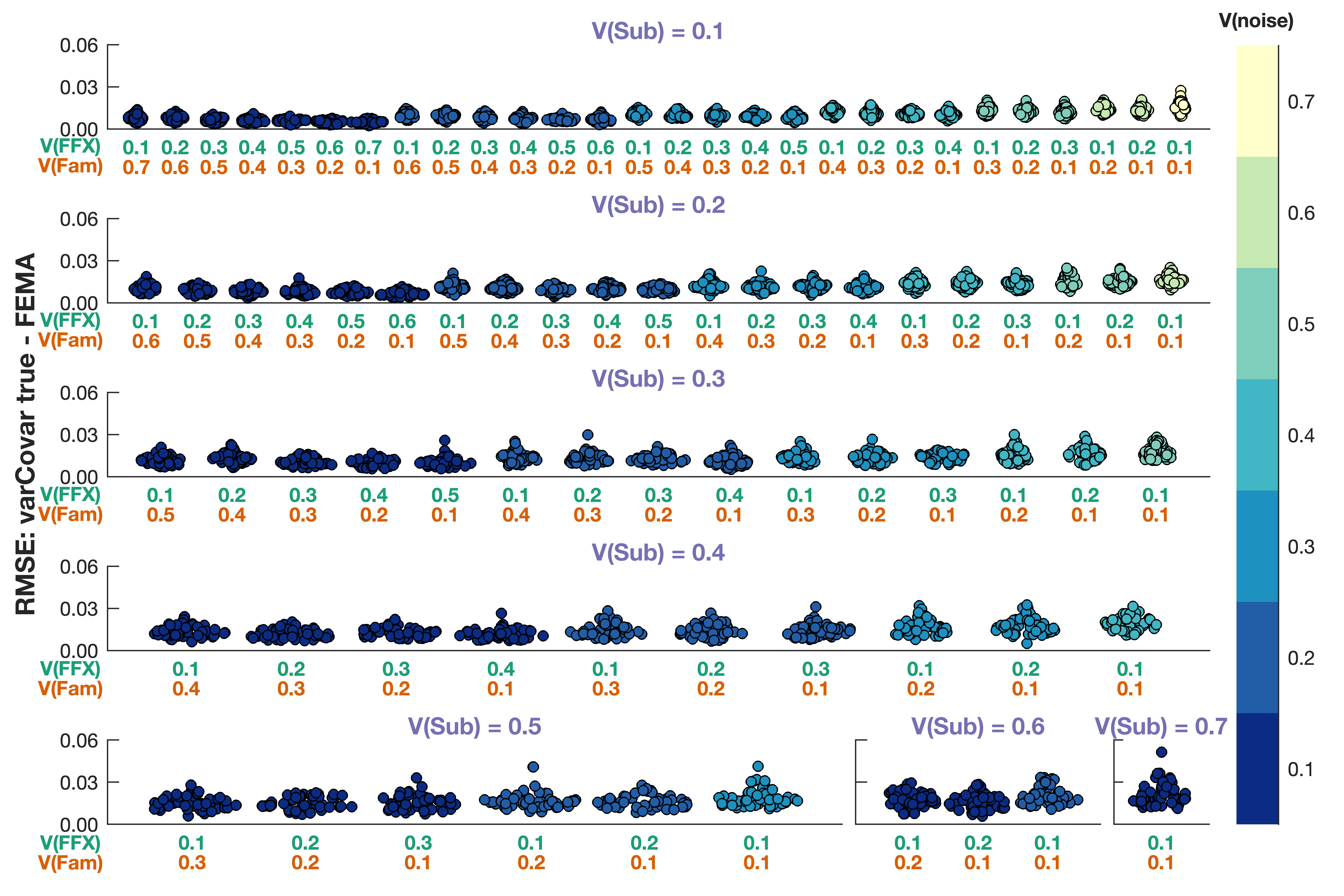

Supplement: S5 Fig — Each panel shows a simulation condition with the amount of variance in the phenotypes explained by the subject effect V(Sub) shown on the top and the amounts of variances explained by fixed effects and family effects V(FFX) and V(Fam) labeled on the x-axis. Each point shows the root mean squared error (RMSE) between the simulated ground truth and the estimates from FEMA, repeated 50 times for each simulation scenario, color-coded by the amount of noise in the phenotype. (TIFF) [file pgen.1012184.s017.tiff]

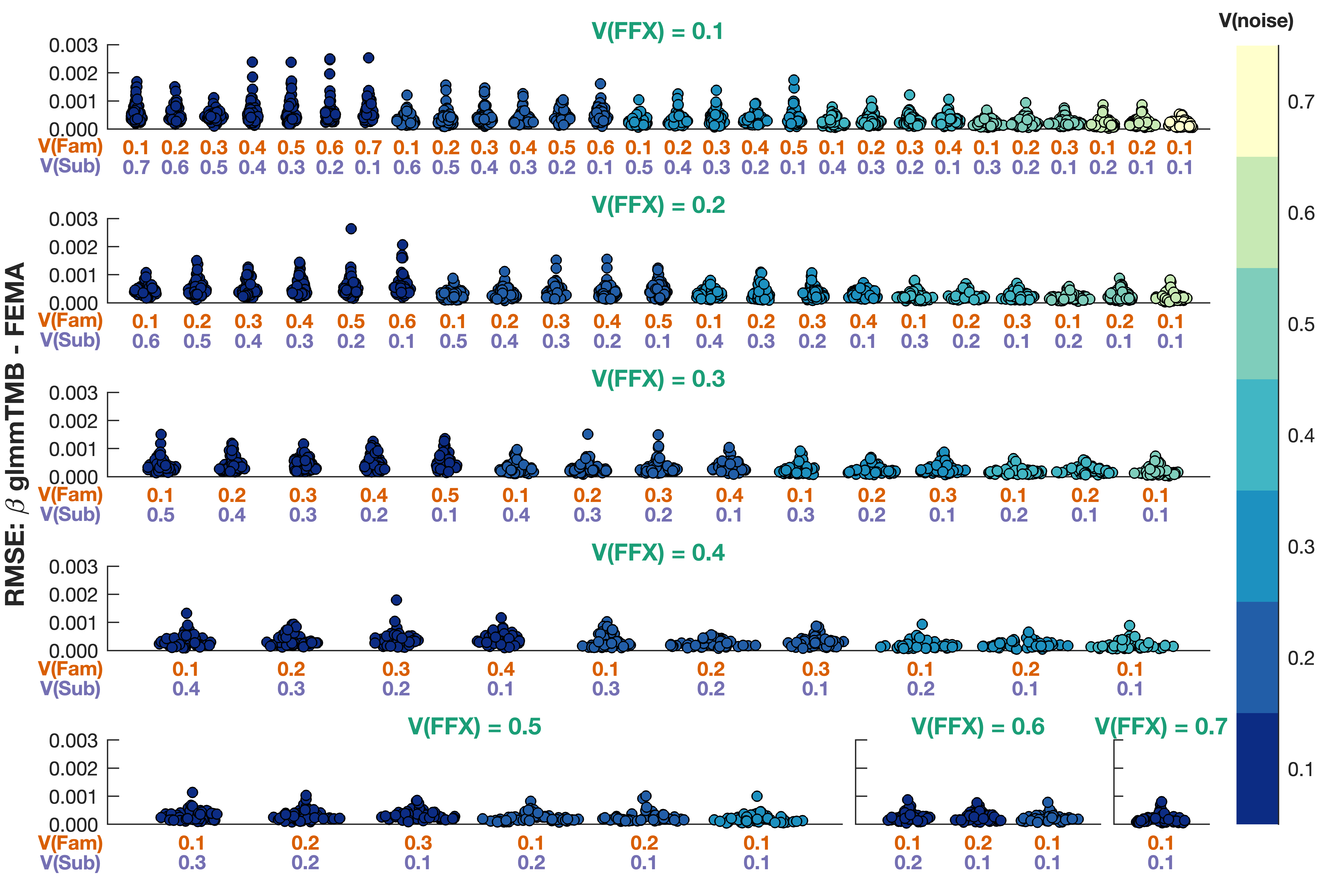

Supplement: S6 Fig — Each panel shows a simulation condition with the amount of variance in the phenotypes explained by the fixed effects V(FFX) shown on the top and the amounts of variances explained by family effects and subject effects V(Fam) and V(Sub) labeled on the x-axis. Each point shows the root mean squared error (RMSE) between the estimates from glmmTMB and the estimates from FEMA, repeated 50 times for each simulation scenario, color-coded by the amount of noise in the phenotype. (TIFF) [file pgen.1012184.s018.tiff]

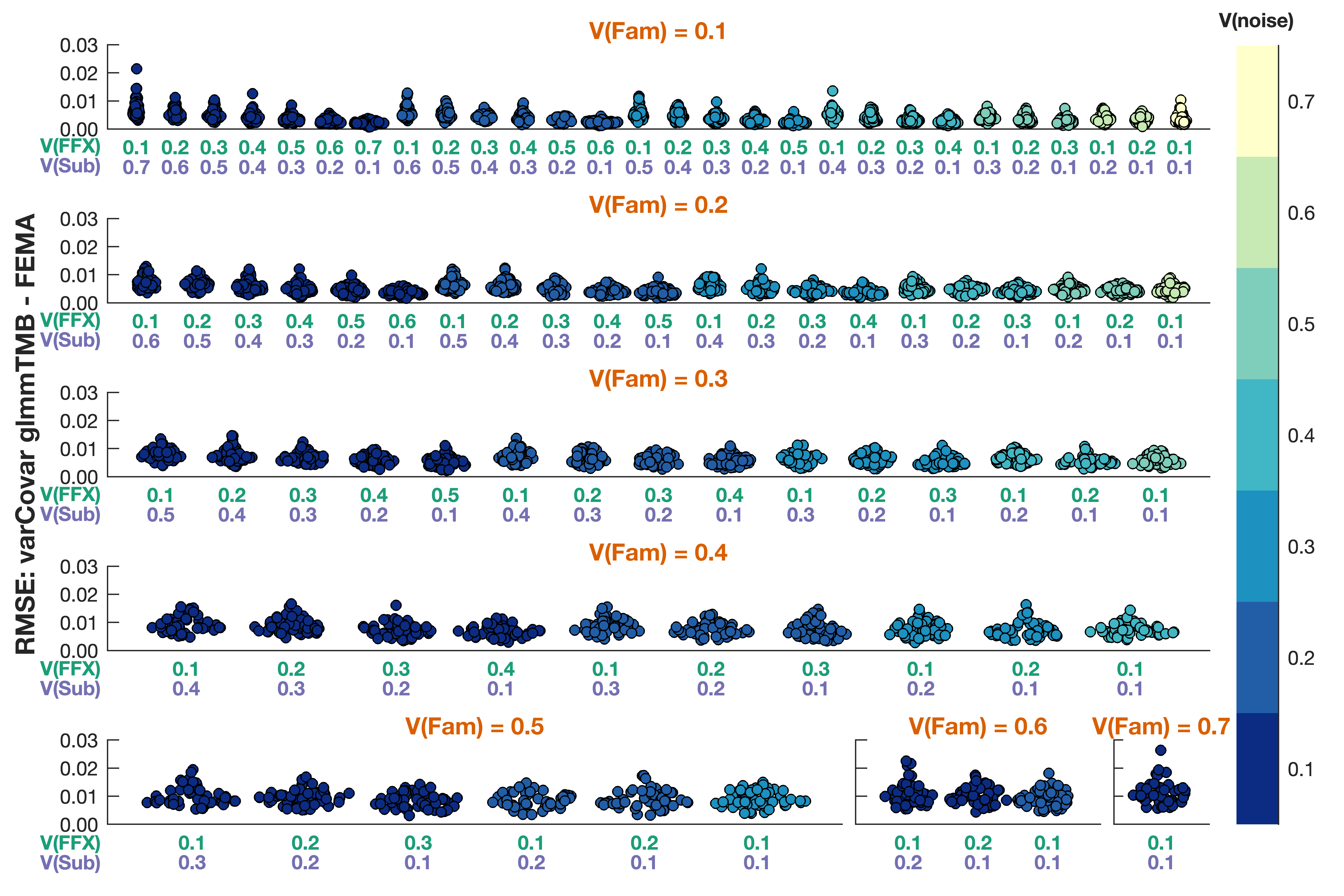

Supplement: S7 Fig — Each panel shows a simulation condition with the amount of variance in the phenotypes explained by the family effects V(Fam) shown on the top and the amounts of variances explained by fixed effects and subject effects V(FFX) and V(Sub) labeled on the x-axis. Each point shows the root mean squared error (RMSE) between the estimates from glmmTMB and the estimates from FEMA, repeated 50 times for each simulation scenario, color-coded by the amount of noise in the phenotype. (TIFF) [file pgen.1012184.s019.tiff]

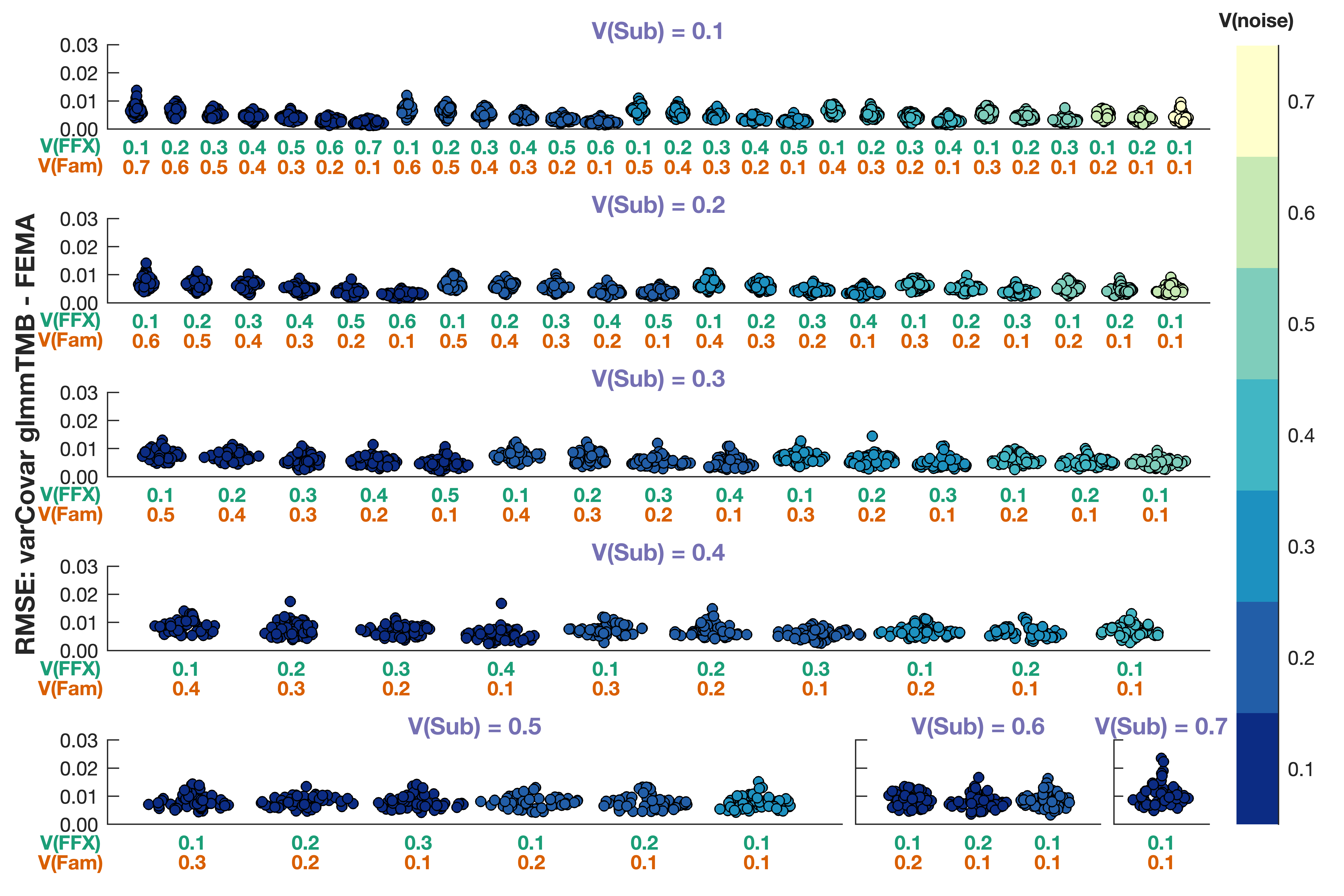

Supplement: S8 Fig — Each panel shows a simulation condition with the amount of variance in the phenotypes explained by the subject effects V(Sub) shown on the top and the amounts of variances explained by fixed effects and family effects V(FFX) and V(Fam) labeled on the x-axis. Each point shows the root mean squared error (RMSE) between the estimates from glmmTMB and the estimates from FEMA, repeated 50 times for each simulation scenario, color-coded by the amount of noise in the phenotype. (TIFF) [file pgen.1012184.s020.tiff]

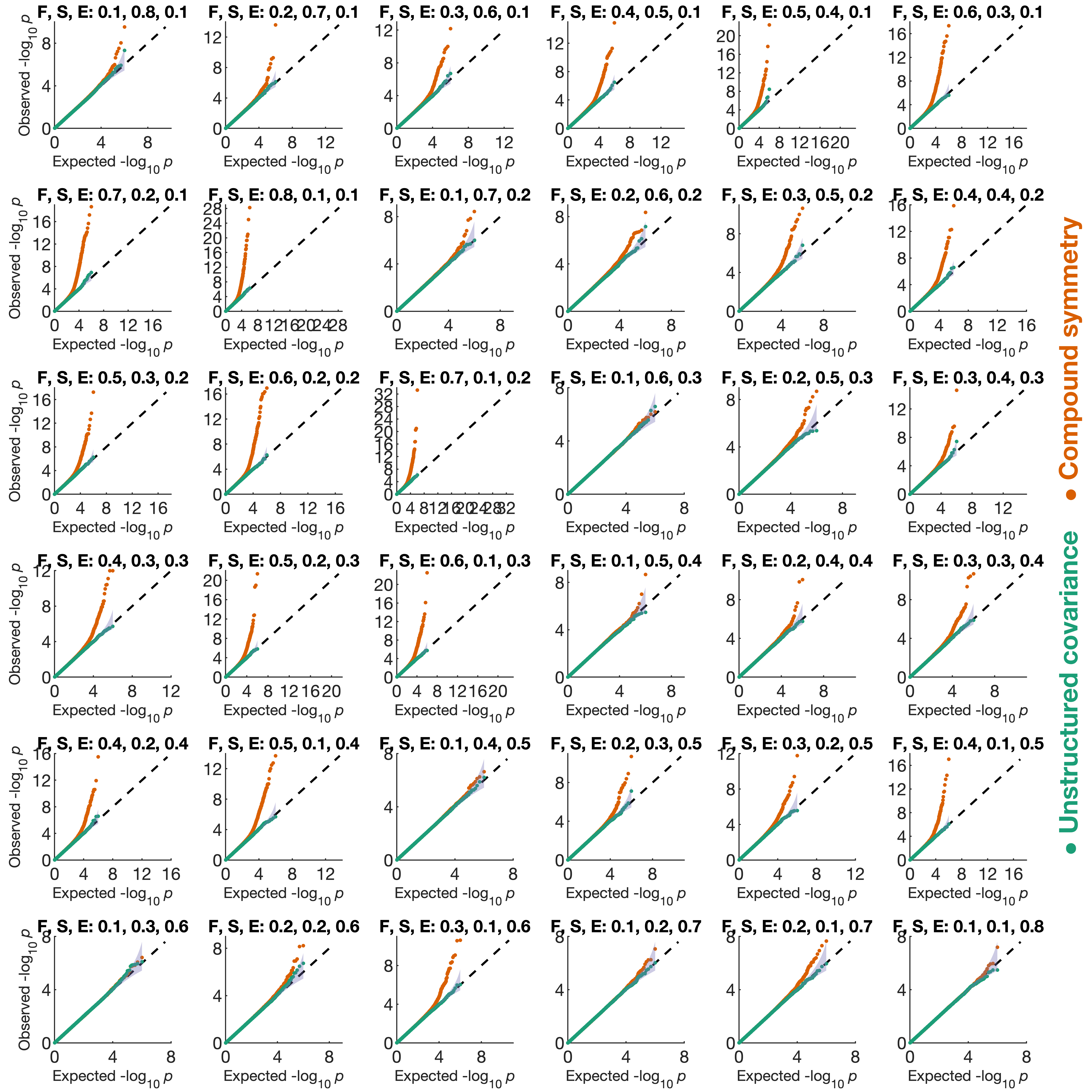

Supplement: S9 Fig — The simulation setting is indicated on the top of each Q-Q plot indicating the amounts of variances (in the phenotype) explained by family (F), subject (S), and noise (E); the x-axes indicate the expected −log10(p) values under the null hypothesis while the y-axes show the observed −log10(p) values across 1000 repeats, 100 X variables, and 10 y variables. The purple filled area indicates the 95% confidence interval based on inverse beta distribution. (TIFF) [file pgen.1012184.s021.tiff]

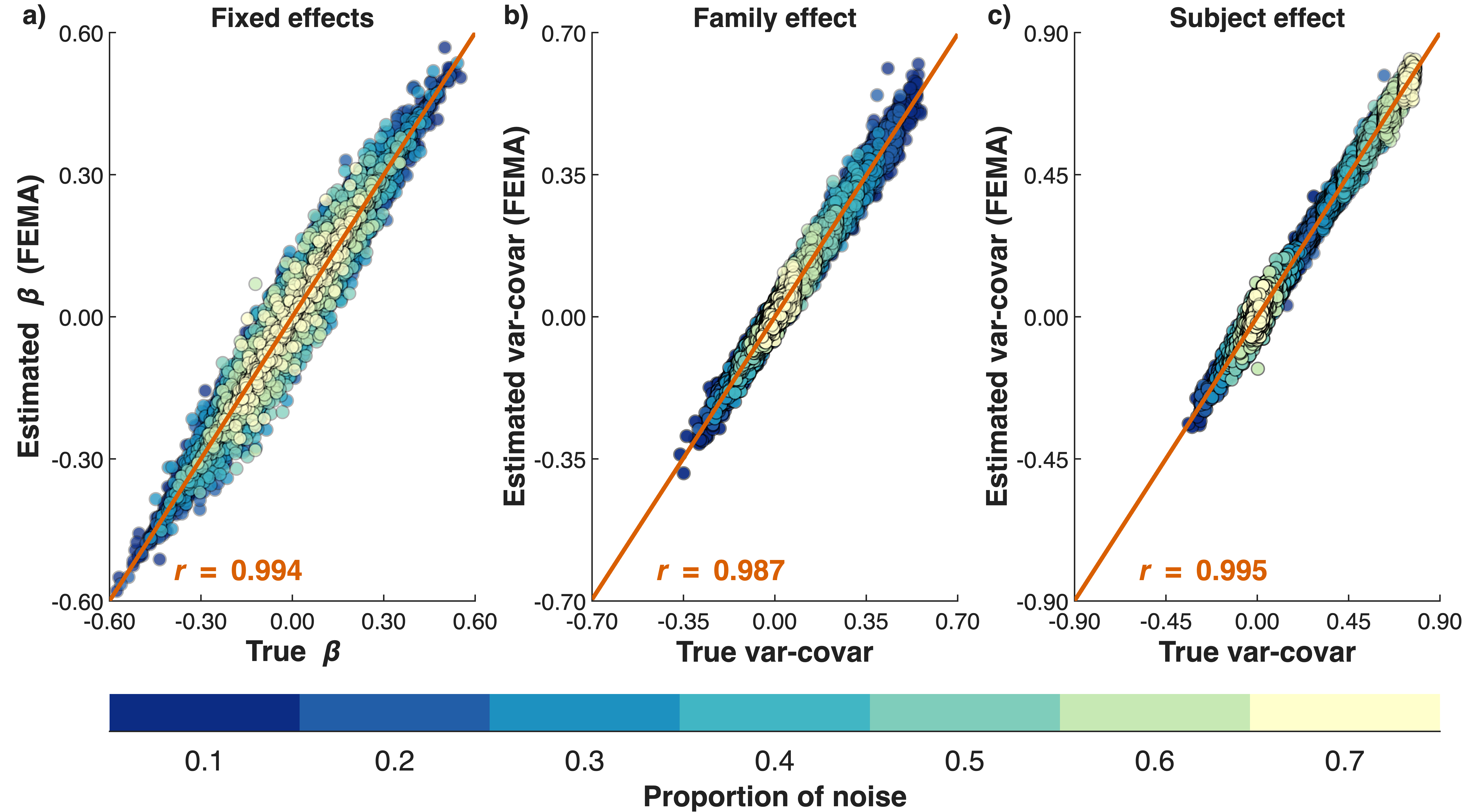

Supplement: S10 Fig — Scatterplots of estimated parameters against ground truth across 50 iterations and 84 simulation settings (nobs=12,000; minnumObs=500). (TIFF) [file pgen.1012184.s022.tiff]

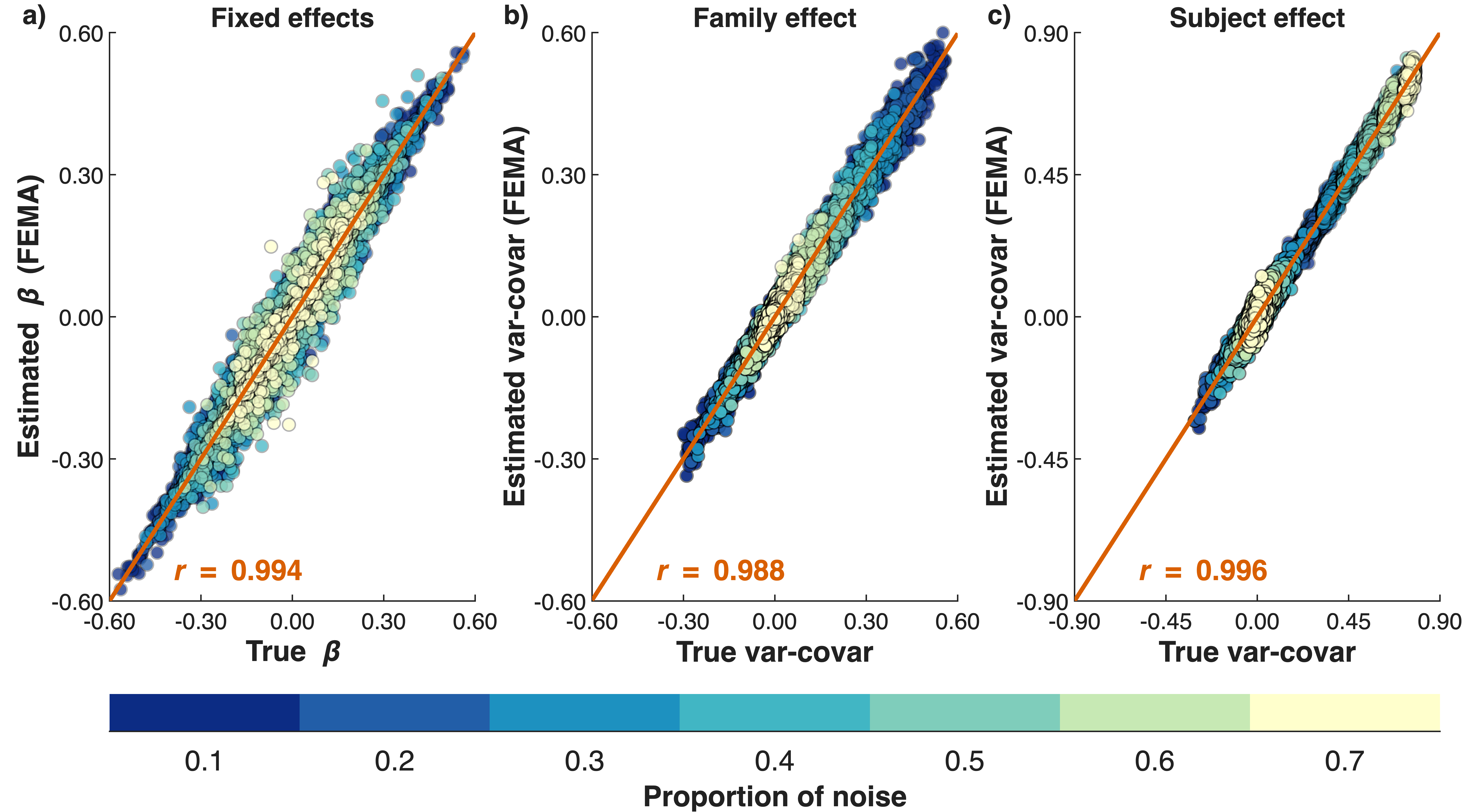

Supplement: S11 Fig — Scatterplots of estimated parameters against ground truth across 50 iterations and 84 simulation settings (nobs=12,000; minnumObs=600). (TIFF) [file pgen.1012184.s023.tiff]

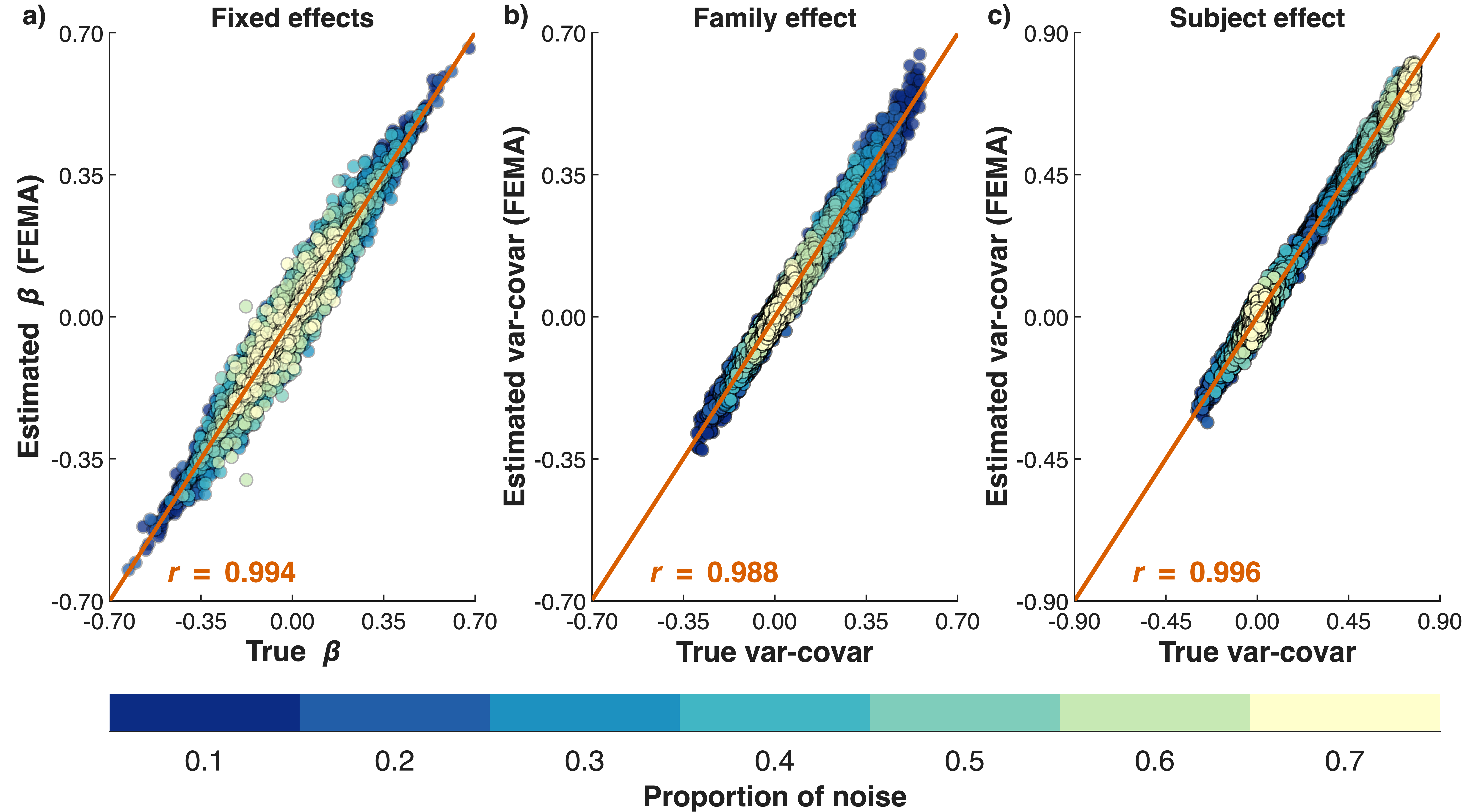

Supplement: S12 Fig — Scatterplots of estimated parameters against ground truth across 50 iterations and 84 simulation settings (nobs=12,000; minnumObs=700). (TIFF) [file pgen.1012184.s024.tiff]

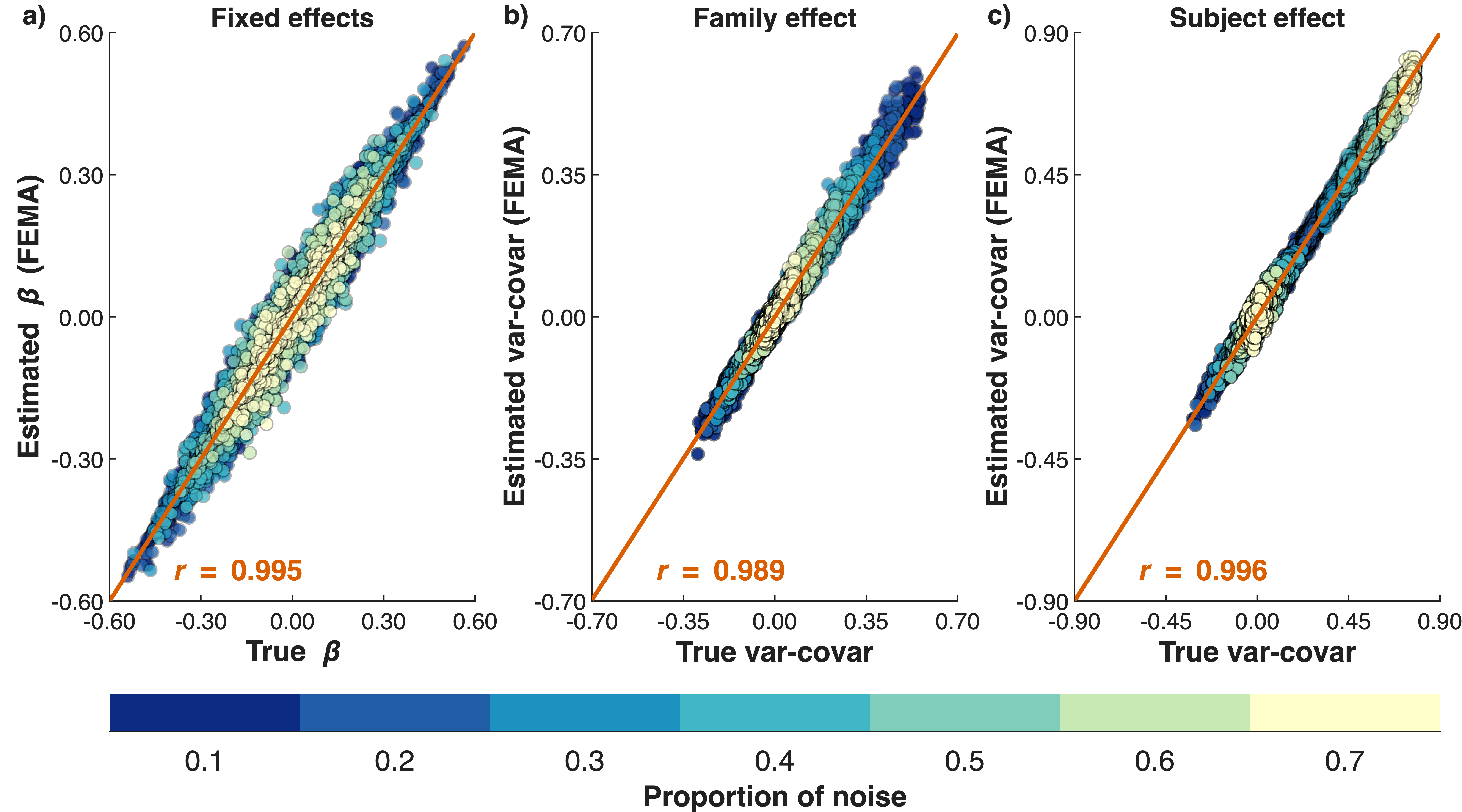

Supplement: S13 Fig — Scatterplots of estimated parameters against ground truth across 50 iterations and 84 simulation settings (nobs=12,000; minnumObs=800). (TIFF) [file pgen.1012184.s025.tiff]

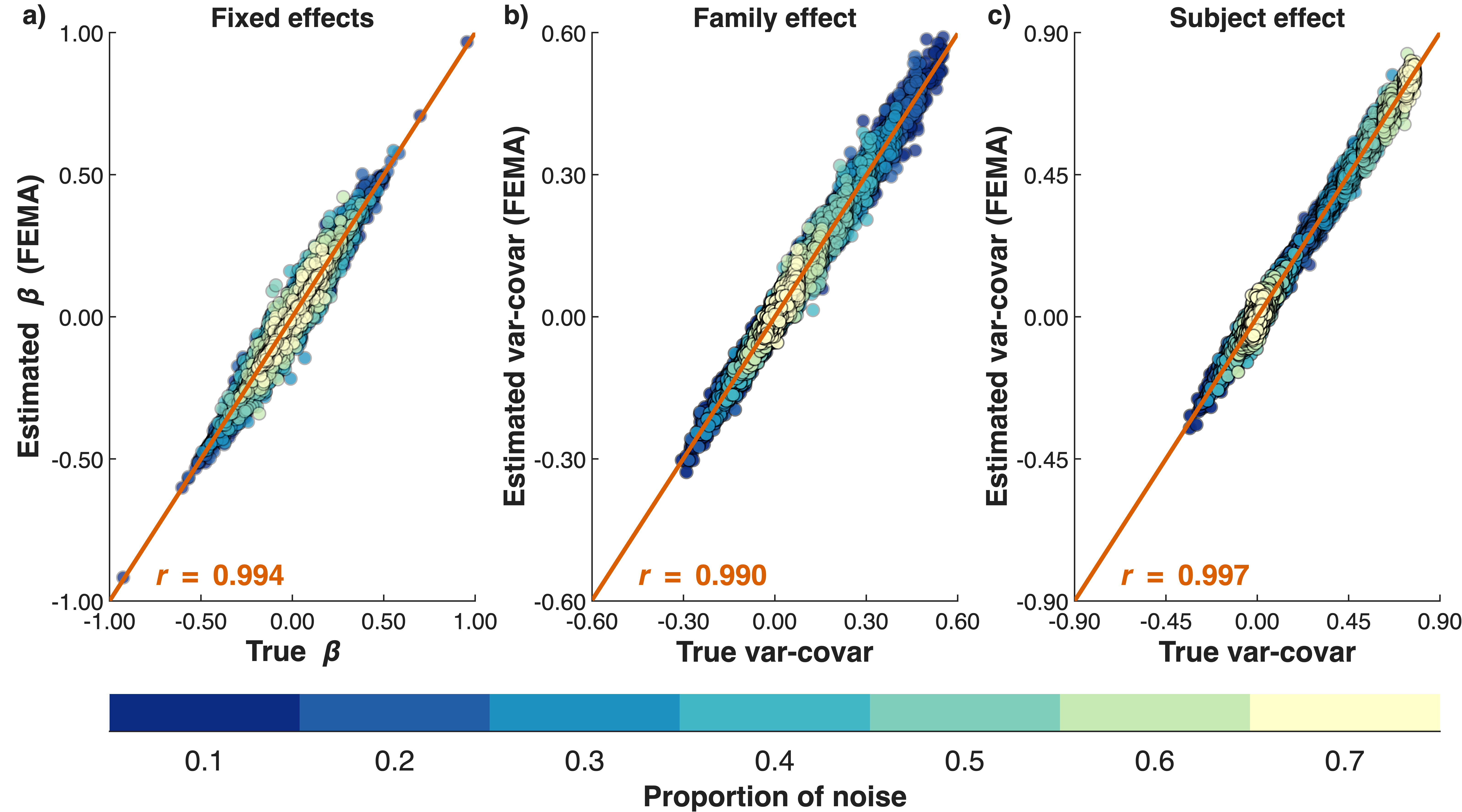

Supplement: S14 Fig — Scatterplots of estimated parameters against ground truth across 50 iterations and 84 simulation settings (nobs=15,000; minnumObs=500). (TIFF) [file pgen.1012184.s026.tiff]

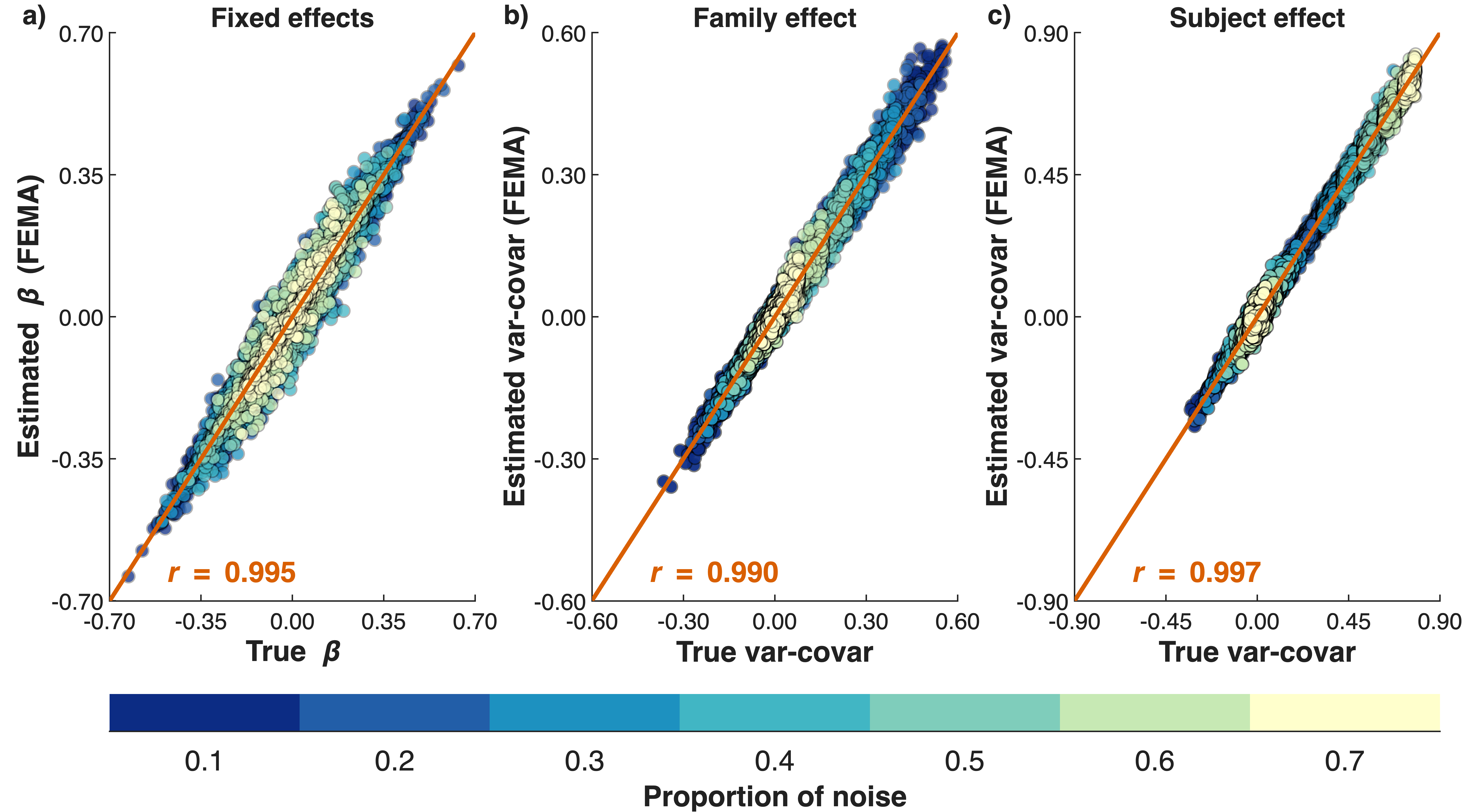

Supplement: S15 Fig — Scatterplots of estimated parameters against ground truth across 50 iterations and 84 simulation settings (nobs=15,000; minnumObs=600). (TIFF) [file pgen.1012184.s027.tiff]

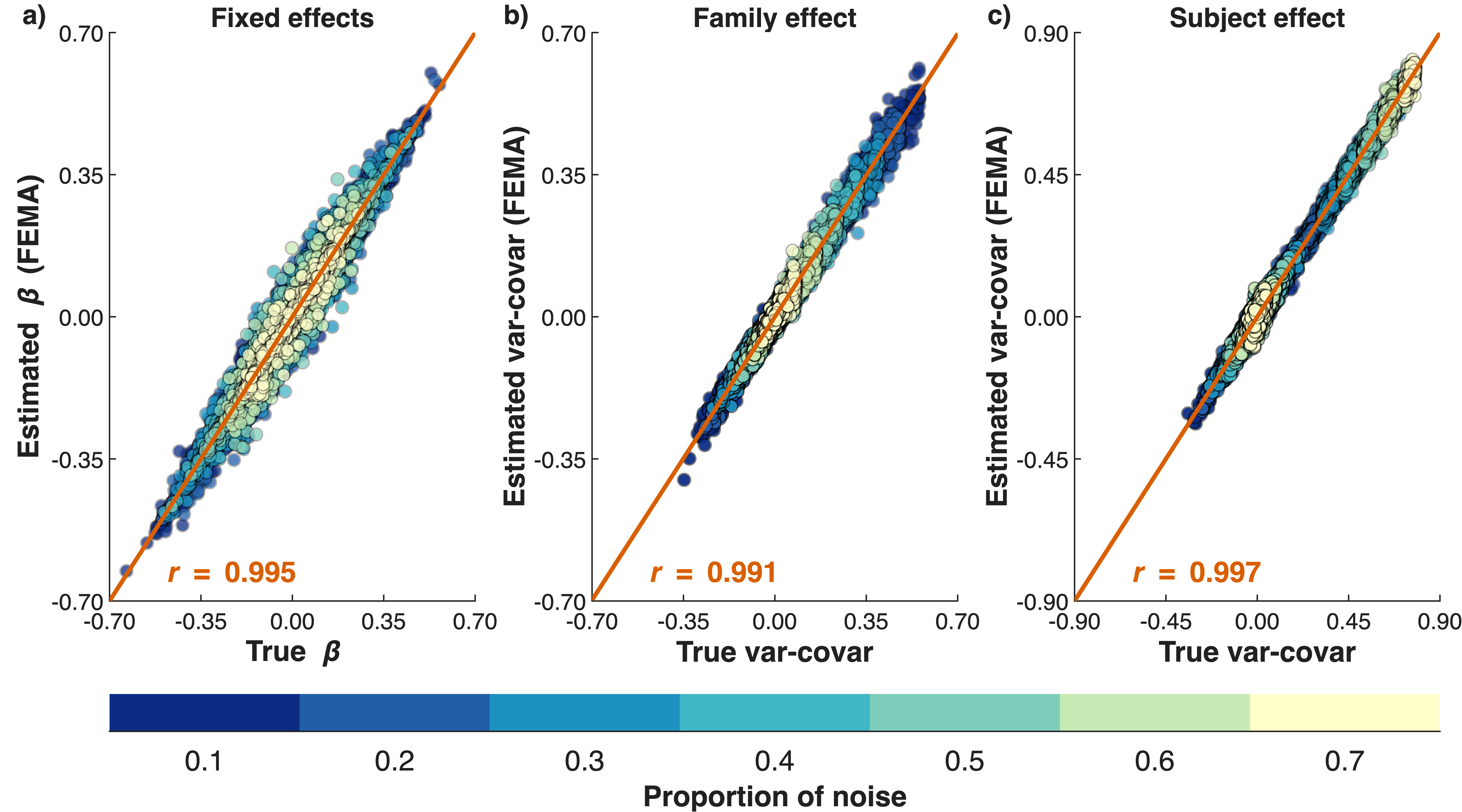

Supplement: S16 Fig — Scatterplots of estimated parameters against ground truth across 50 iterations and 84 simulation settings (nobs=15,000; minnumObs=700). (TIFF) [file pgen.1012184.s028.tiff]

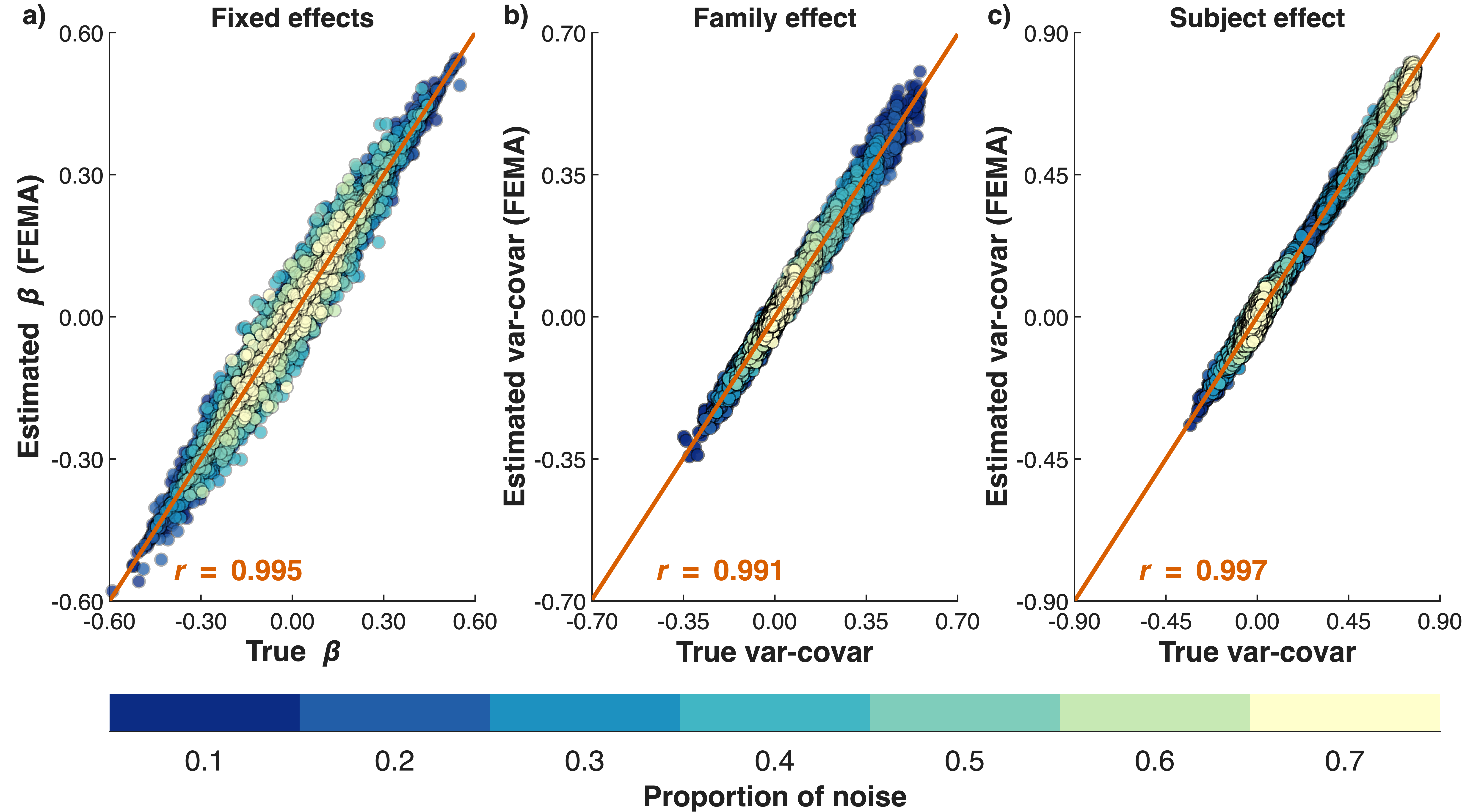

Supplement: S17 Fig — Scatterplots of estimated parameters against ground truth across 50 iterations and 84 simulation settings (nobs=15,000; minnumObs=800). (TIFF) [file pgen.1012184.s029.tiff]

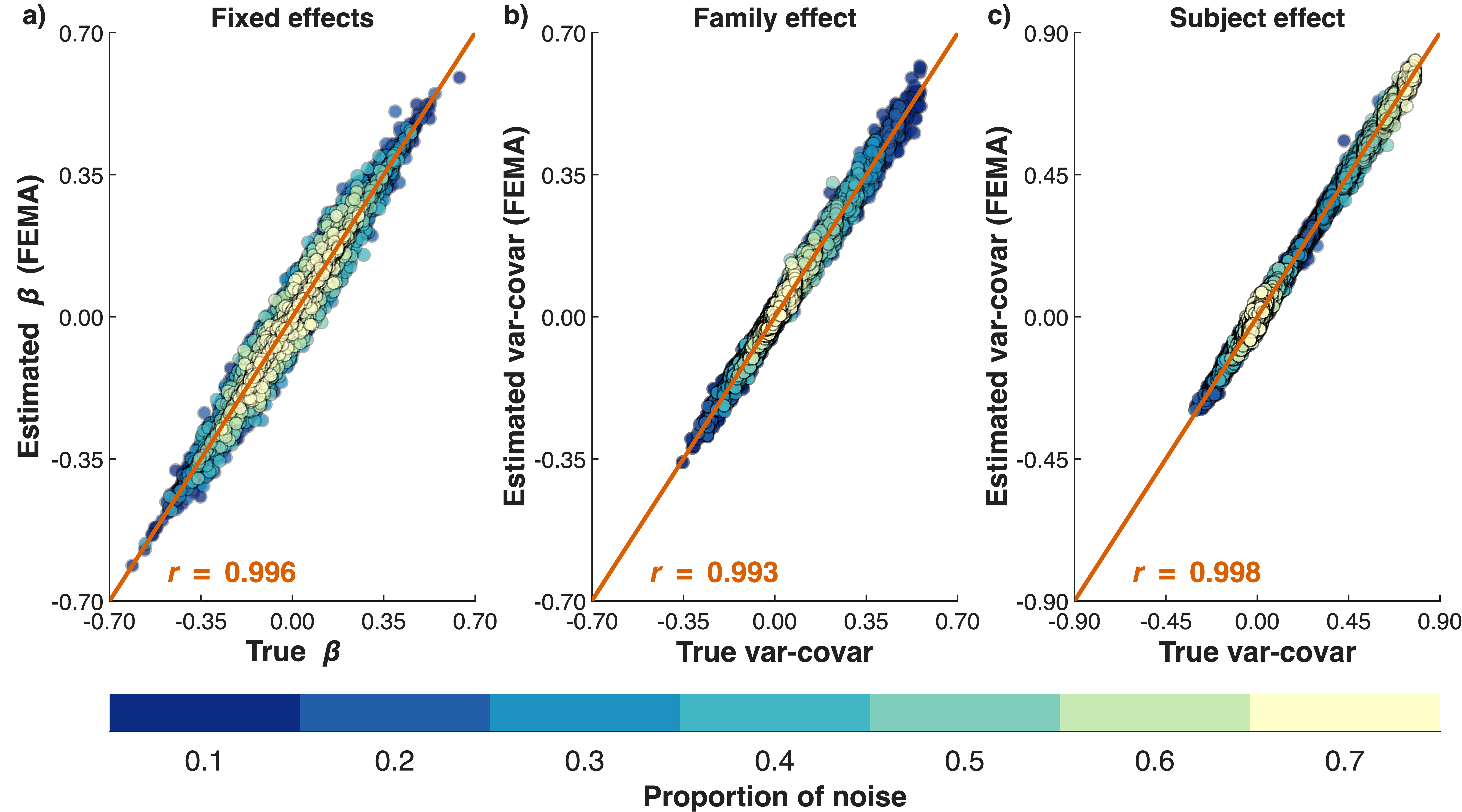

Supplement: S18 Fig — Scatterplots of estimated parameters against ground truth across 50 iterations and 84 simulation settings (nobs=18,000; minnumObs=500). (TIFF) [file pgen.1012184.s030.tiff]

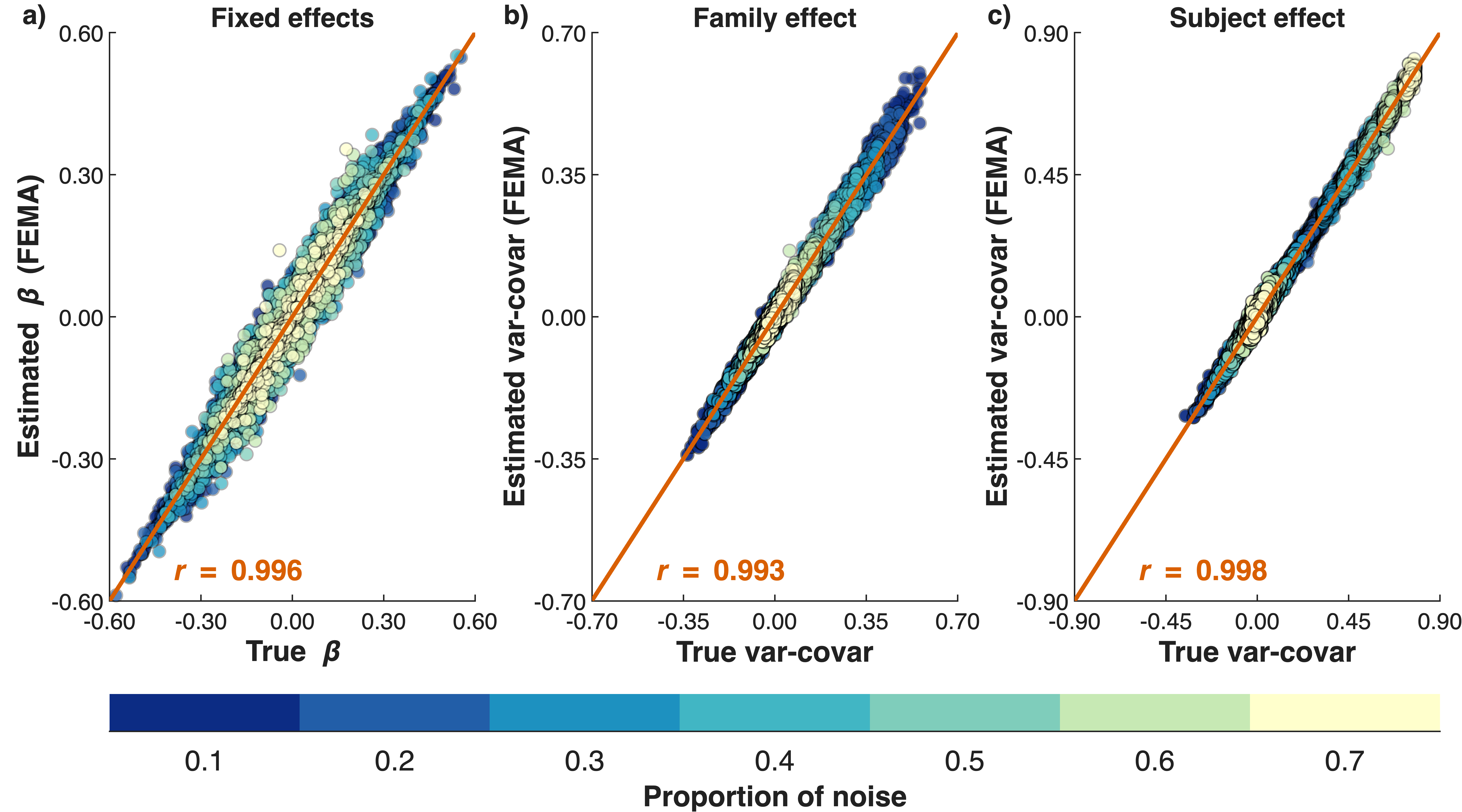

Supplement: S19 Fig — Scatterplots of estimated parameters against ground truth across 50 iterations and 84 simulation settings (nobs=18,000; minnumObs=600). (TIFF) [file pgen.1012184.s031.tiff]

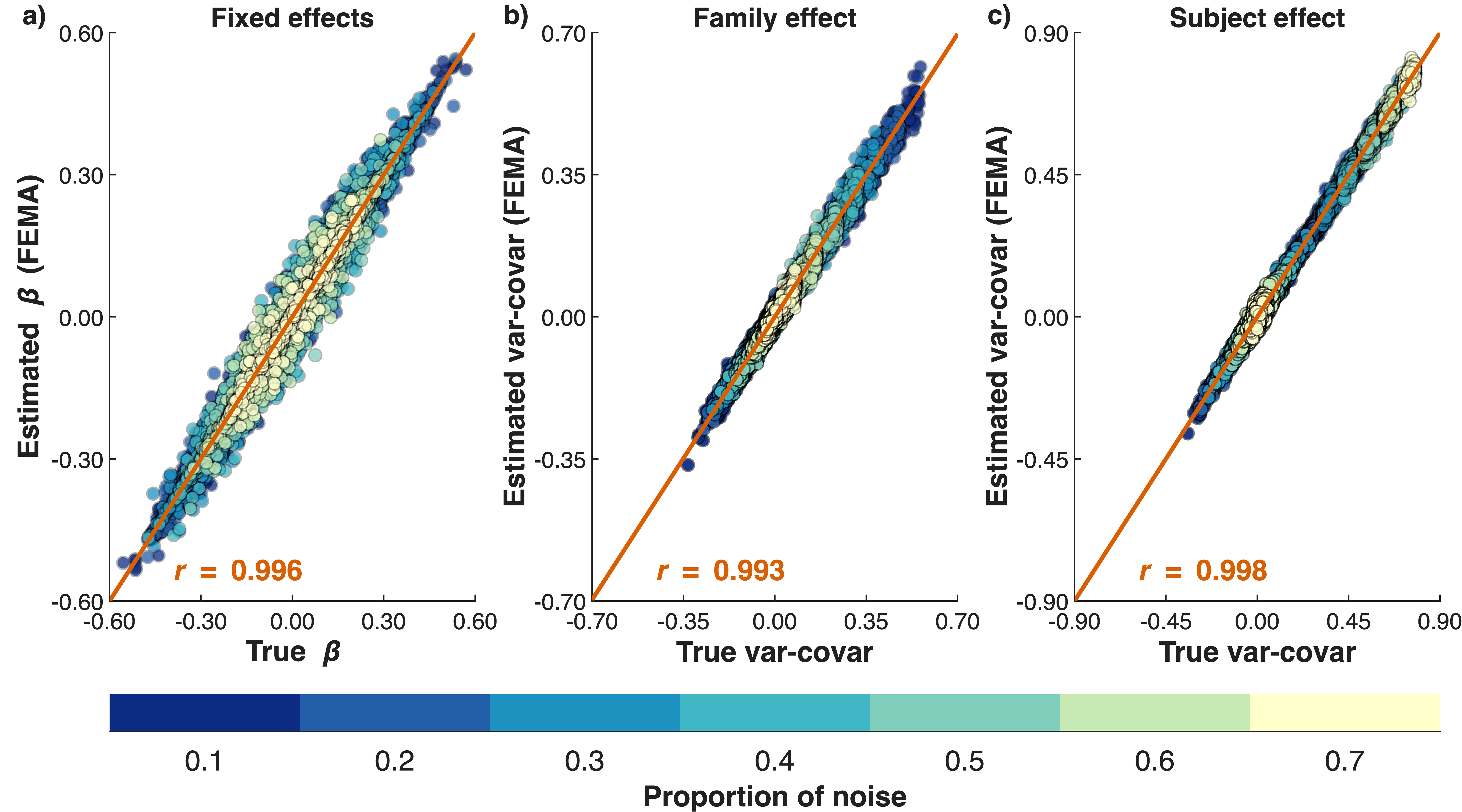

Supplement: S20 Fig — Scatterplots of estimated parameters against ground truth across 50 iterations and 84 simulation settings (nobs=18,000; minnumObs=700). (TIFF) [file pgen.1012184.s032.tiff]

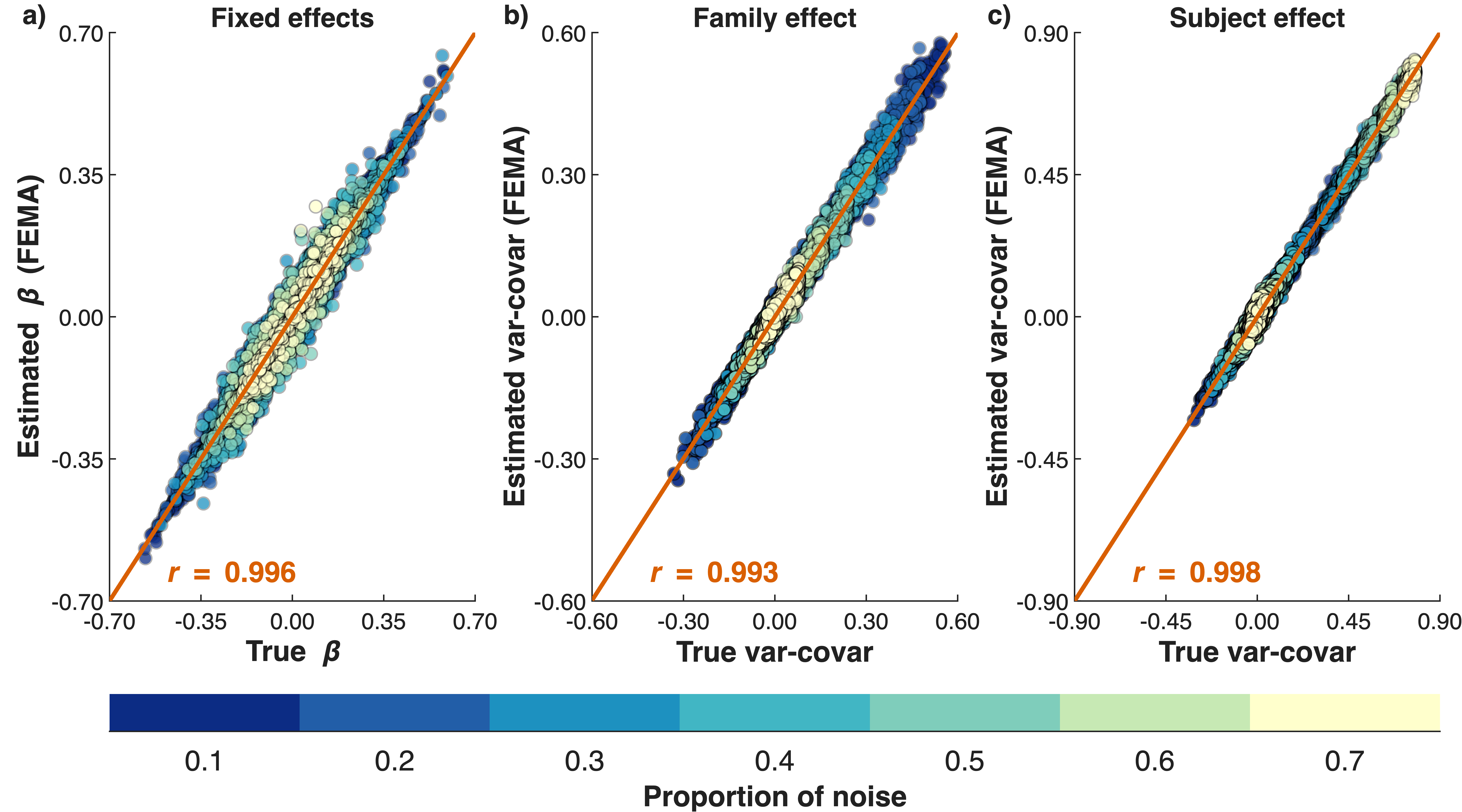

Supplement: S21 Fig — Scatterplots of estimated parameters against ground truth across 50 iterations and 84 simulation settings (nobs=18,000; minnumObs=800). (TIFF) [file pgen.1012184.s033.tiff]

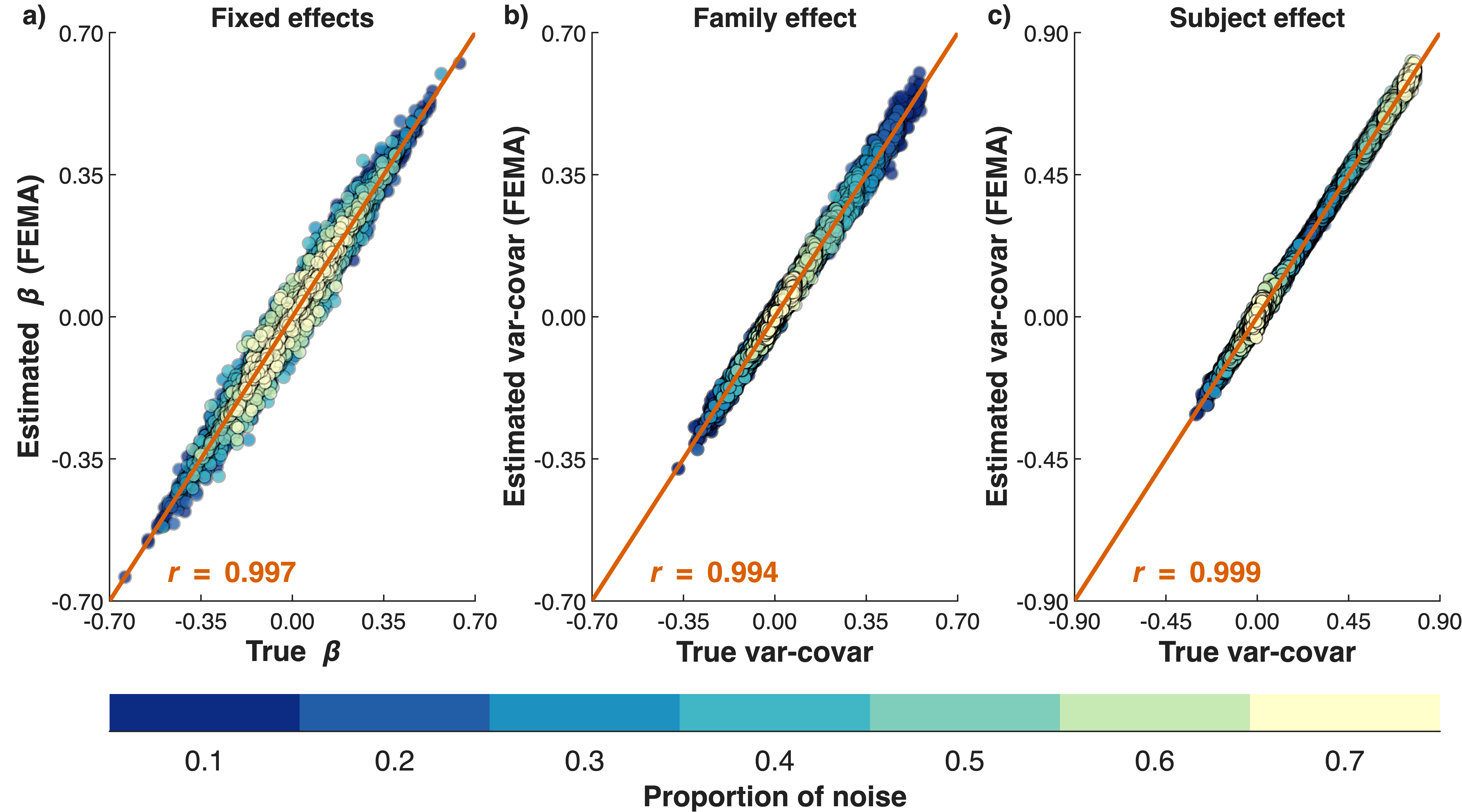

Supplement: S22 Fig — Scatterplots of estimated parameters against ground truth across 50 iterations and 84 simulation settings (nobs=20,000; minnumObs=500). (TIFF) [file pgen.1012184.s034.tiff]

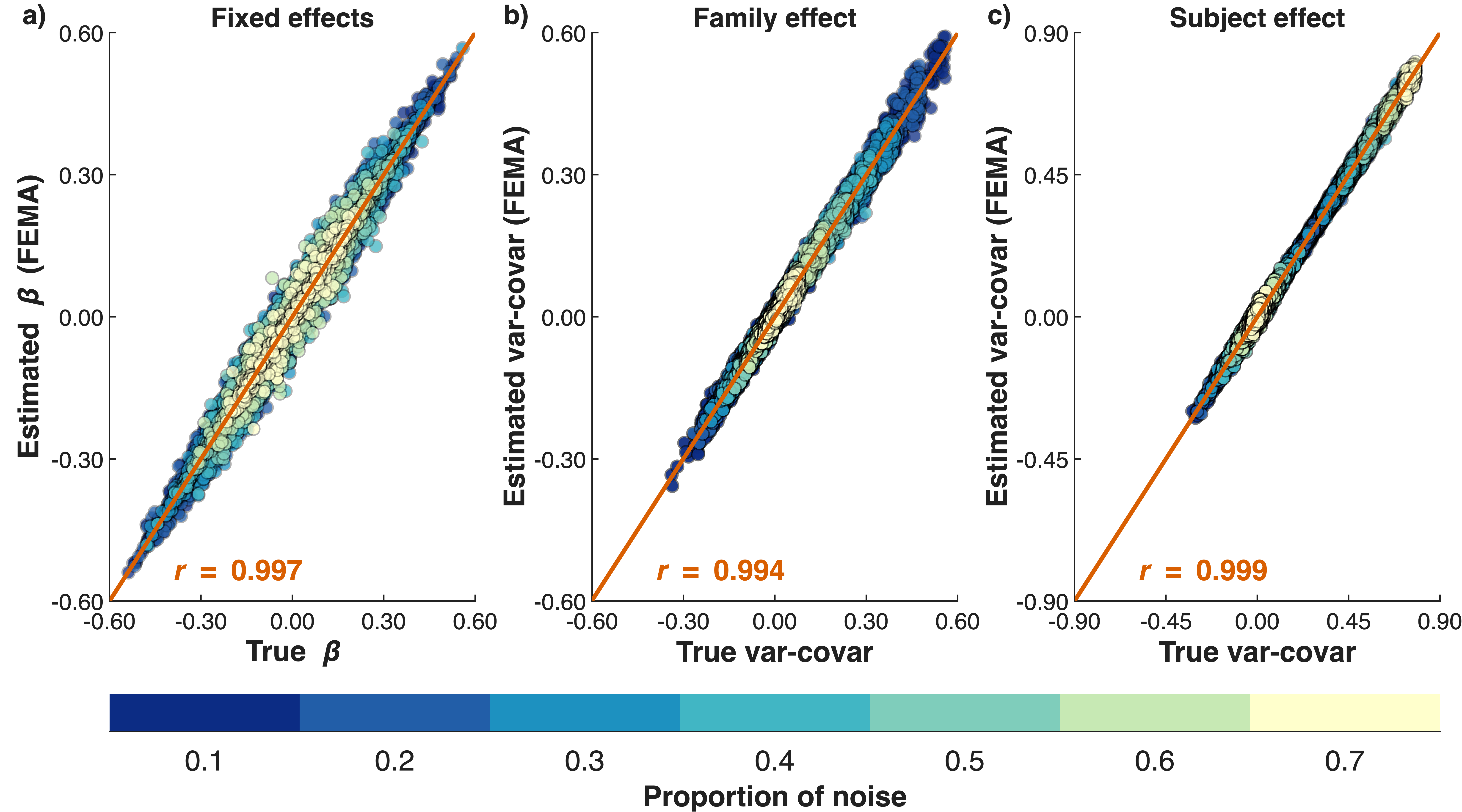

Supplement: S23 Fig — Scatterplots of estimated parameters against ground truth across 50 iterations and 84 simulation settings (nobs=20,000; minnumObs=600). (TIFF) [file pgen.1012184.s035.tiff]

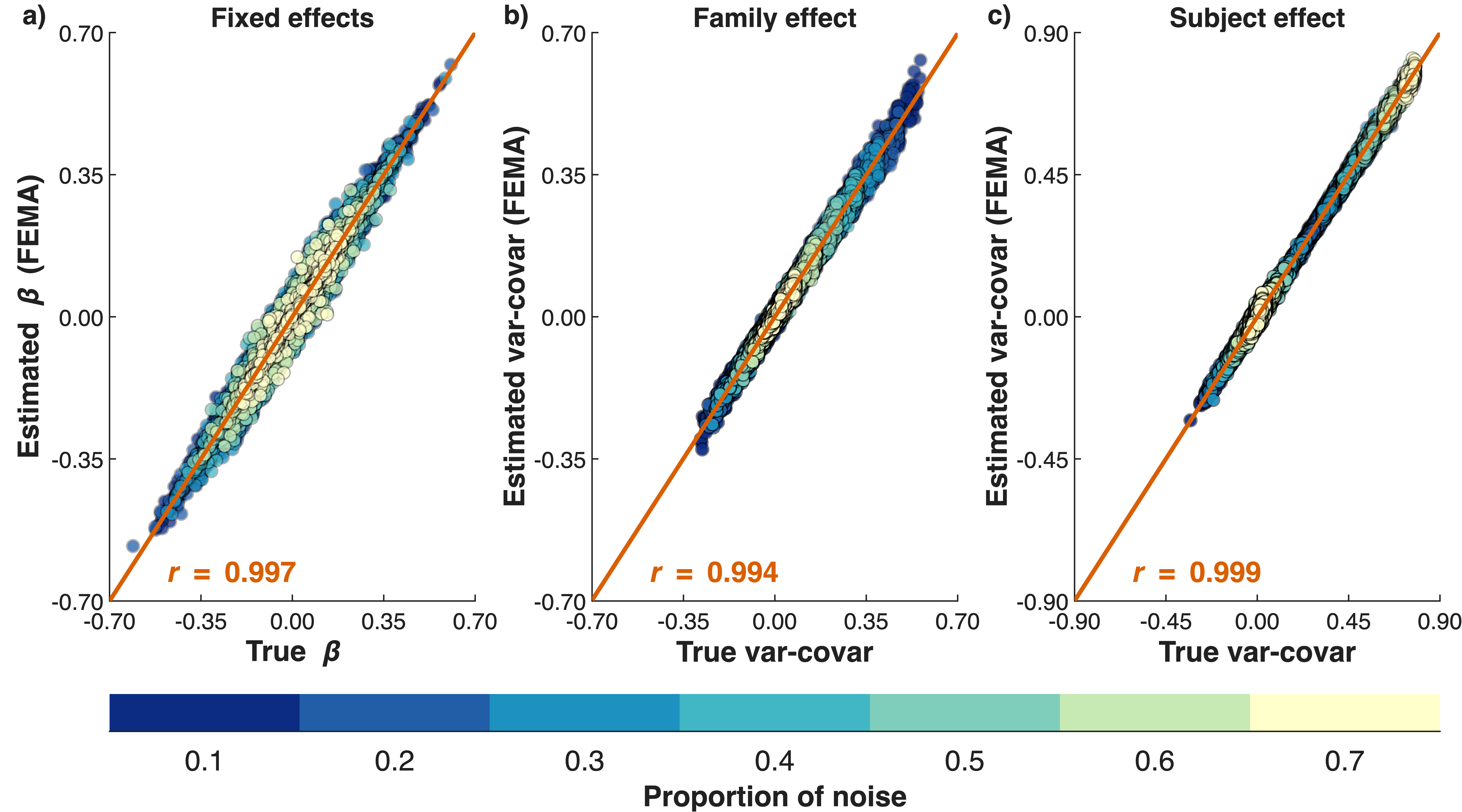

Supplement: S24 Fig — Scatterplots of estimated parameters against ground truth across 50 iterations and 84 simulation settings (nobs=20,000; minnumObs=700). (TIFF) [file pgen.1012184.s036.tiff]

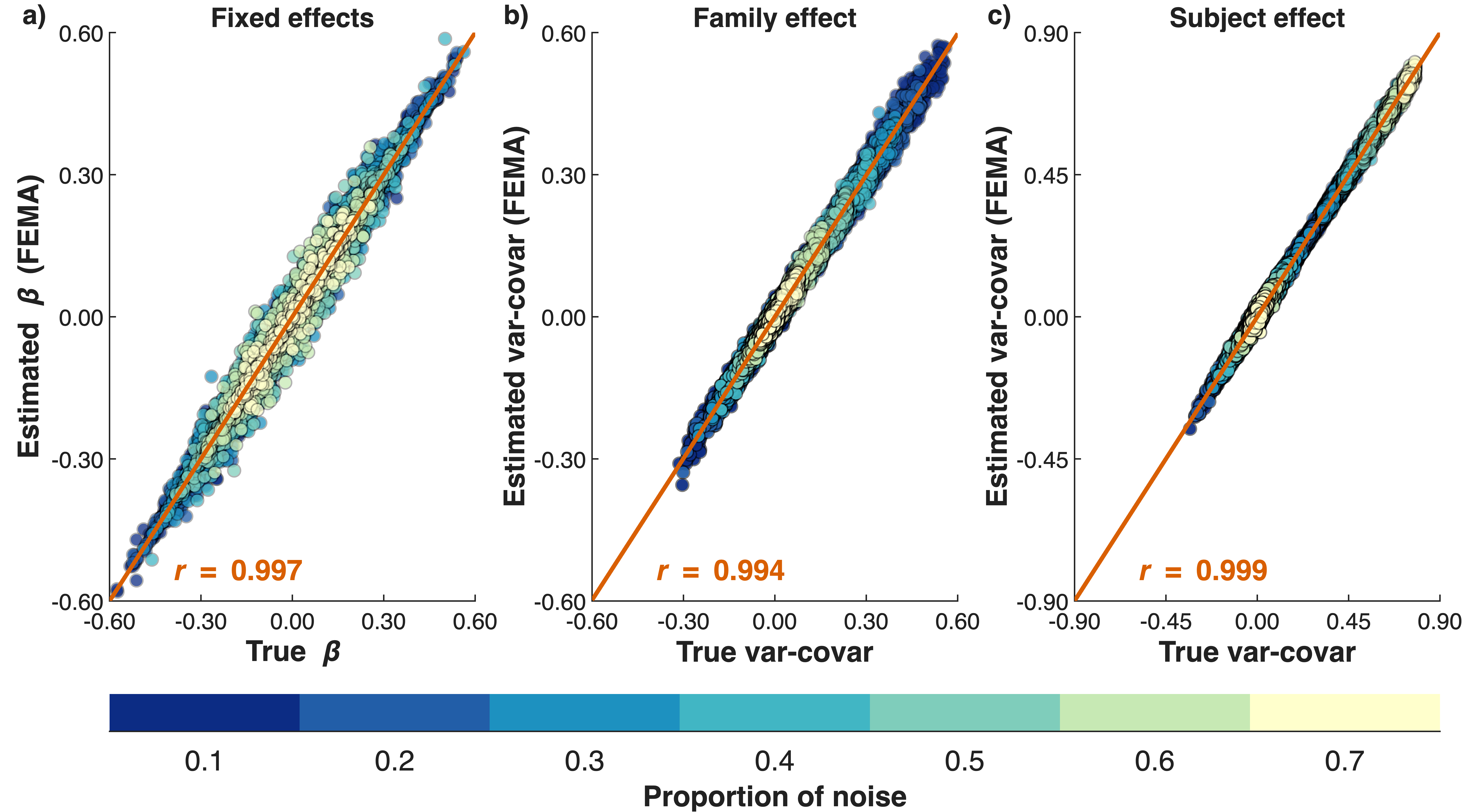

Supplement: S25 Fig — Scatterplots of estimated parameters against ground truth across 50 iterations and 84 simulation settings (nobs=20,000; minnumObs=800). (TIFF) [file pgen.1012184.s037.tiff]

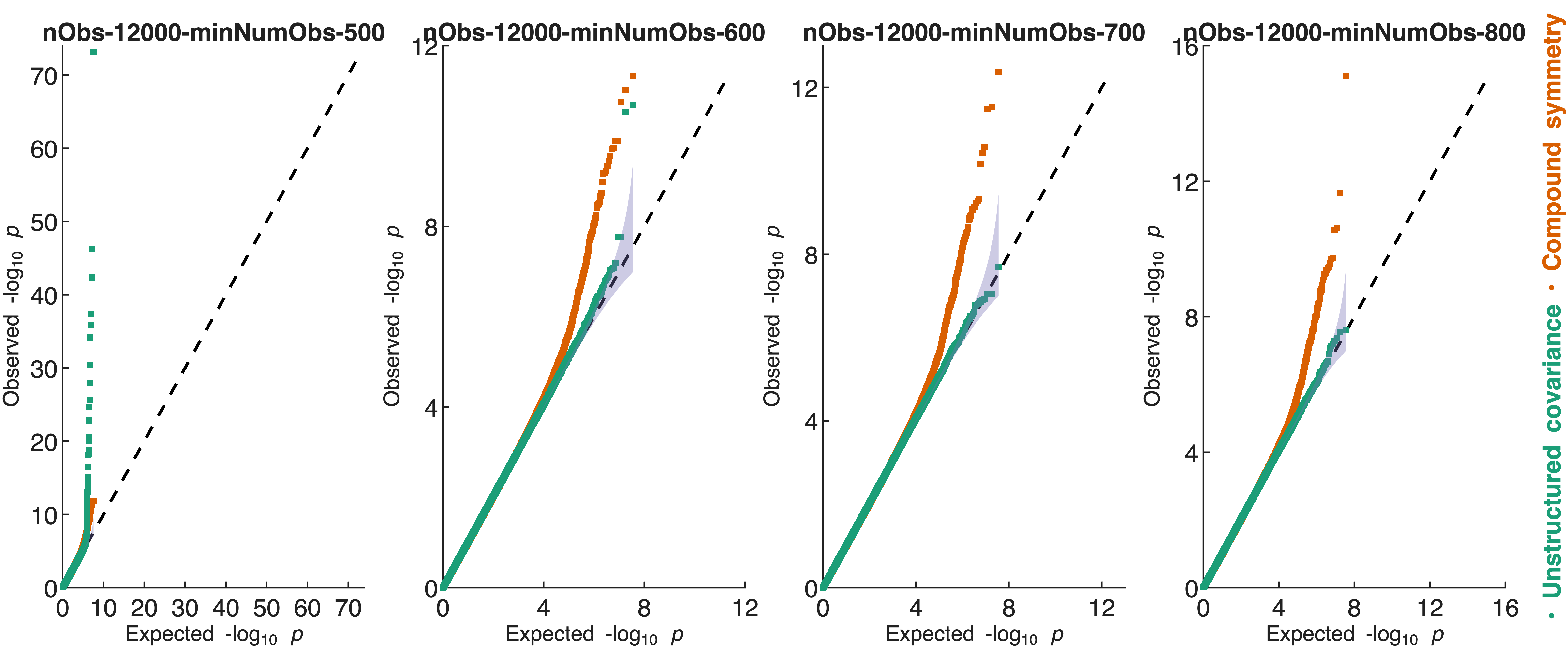

Supplement: S26 Fig — Each panel shows the distribution of −log10p-values across 1000 iterations of 36 simulation settings for unstructured covariance (green) and compound symmetry (orange); each iteration consisted of 100 X variables and 10 outcome variables; the purple filled area indicates the 95% confidence interval based on inverse beta distribution. (TIFF) [file pgen.1012184.s038.tiff]

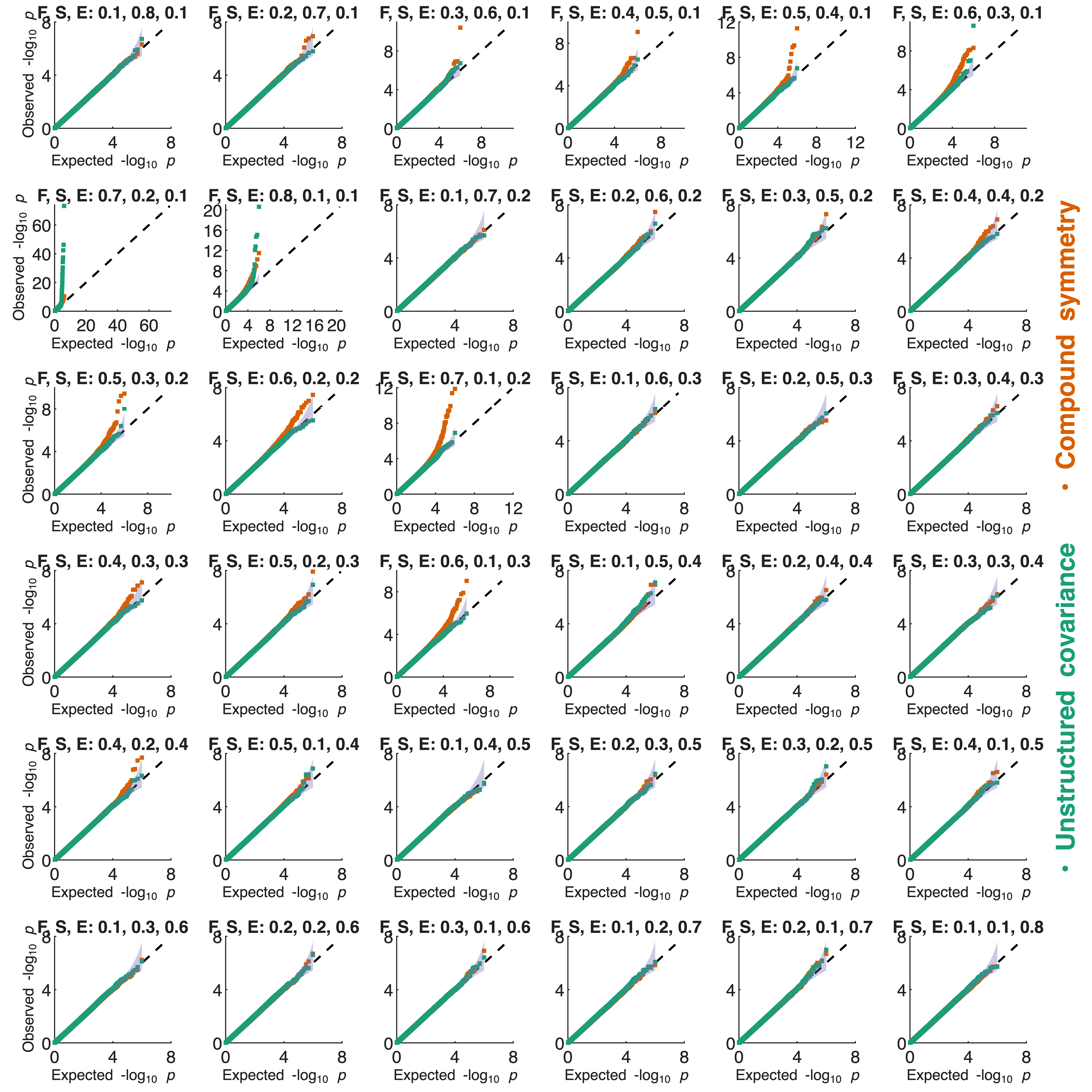

Supplement: S27 Fig — The simulation setting is indicated on the top of each Q-Q plot indicating the amounts of variances (in the phenotype) explained by family (F), subject (S), and noise (E); the x-axes indicate the expected −log10(p) values under the null hypothesis while the y-axes show the observed −log10(p) values across 1000 repeats, 100 X variables, and 10 y variables. The purple filled area indicates the 95% confidence interval based on inverse beta distribution. (TIFF) [file pgen.1012184.s039.tiff]

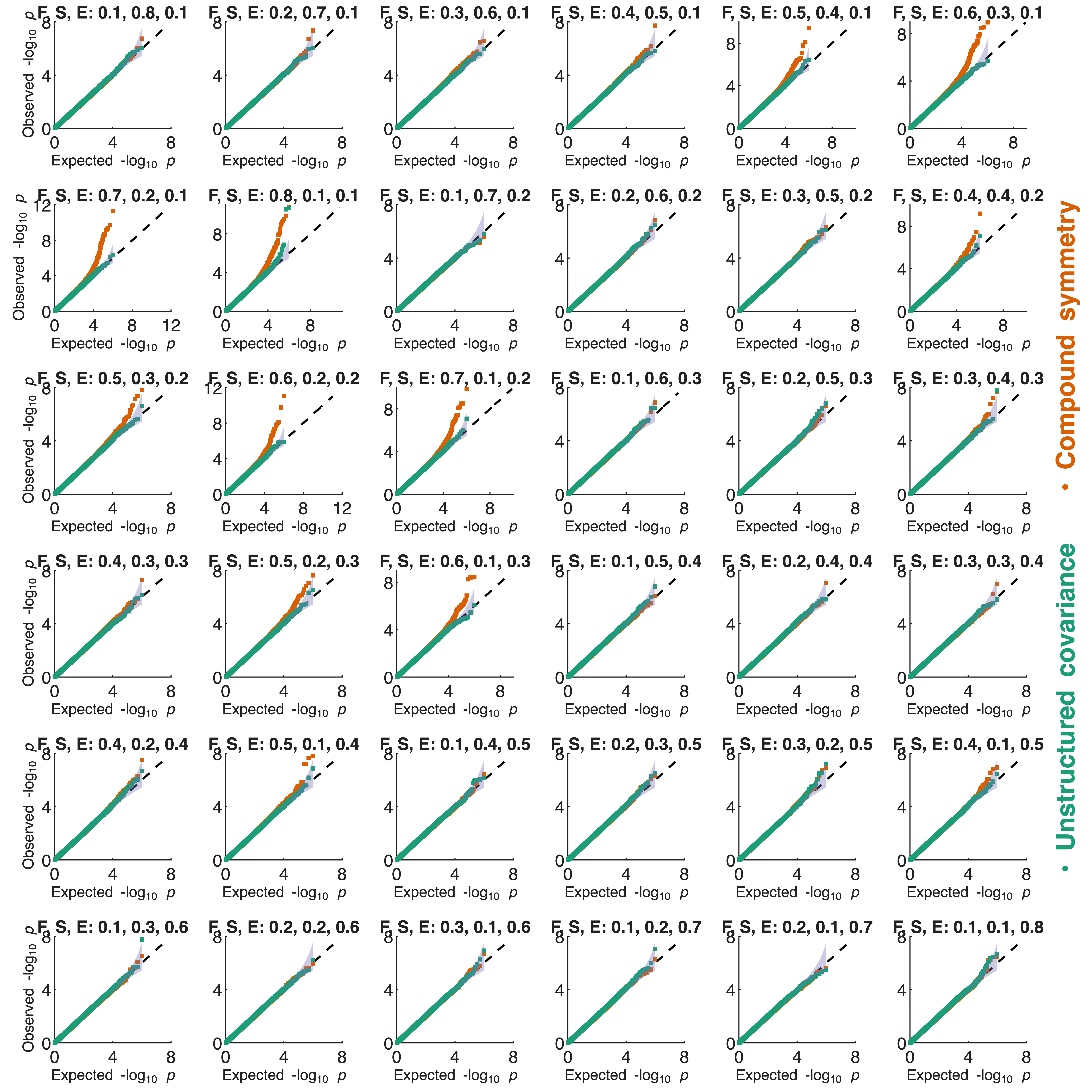

Supplement: S28 Fig — The simulation setting is indicated on the top of each Q-Q plot indicating the amounts of variances (in the phenotype) explained by family (F), subject (S), and noise (E); the x-axes indicate the expected −log10(p) values under the null hypothesis while the y-axes show the observed −log10(p) values across 1000 repeats, 100 X variables, and 10 y variables. The purple filled area indicates the 95% confidence interval based on inverse beta distribution. (TIFF) [file pgen.1012184.s040.tiff]

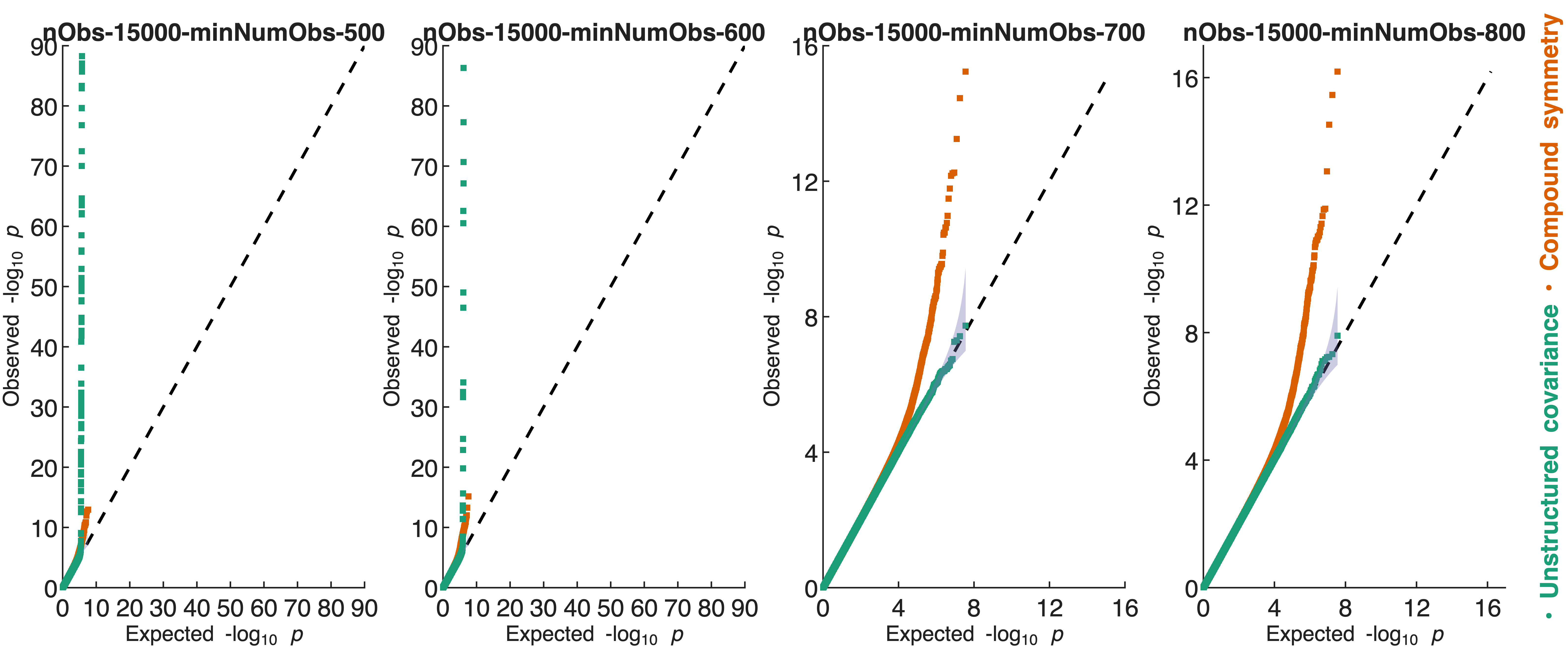

Supplement: S29 Fig — Each panel shows the distribution of −log10p-values across 1000 iterations of 36 simulation settings for unstructured covariance (green) and compound symmetry (orange); each iteration consisted of 100 X variables and 10 outcome variables; the purple filled area indicates the 95% confidence interval based on inverse beta distribution. Note that the y-axis is truncated in the first two panels. (TIFF) [file pgen.1012184.s041.tiff]

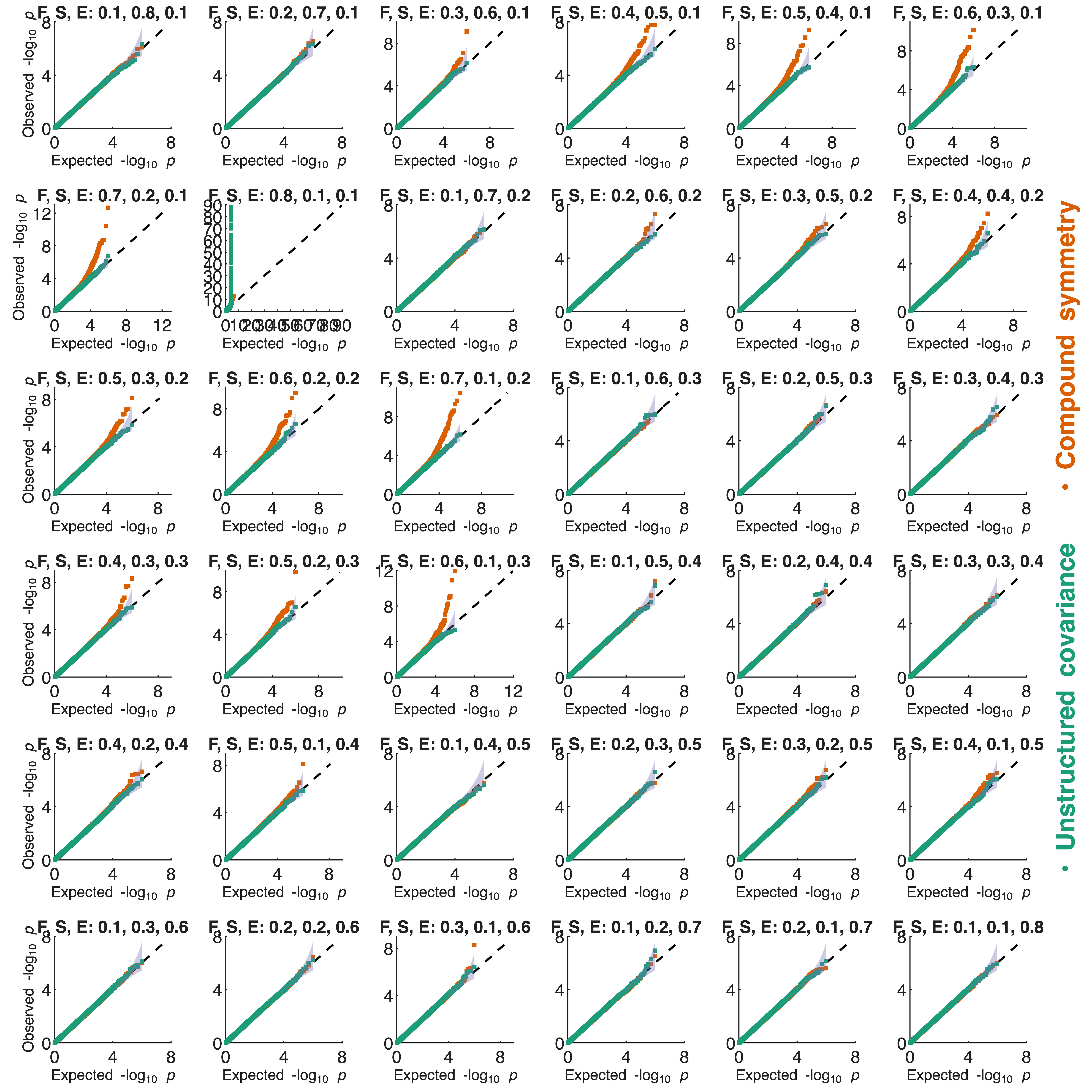

Supplement: S30 Fig — The simulation setting is indicated on the top of each Q-Q plot indicating the amounts of variances (in the phenotype) explained by family (F), subject (S), and noise (E); the x-axes indicate the expected −log10(p) values under the null hypothesis while the y-axes show the observed −log10(p) values across 1000 repeats, 100 X variables, and 10 y variables. The purple filled area indicates the 95% confidence interval based on inverse beta distribution. Note that the y-axis is truncated for the plot in second row, second column. (TIFF) [file pgen.1012184.s042.tiff]

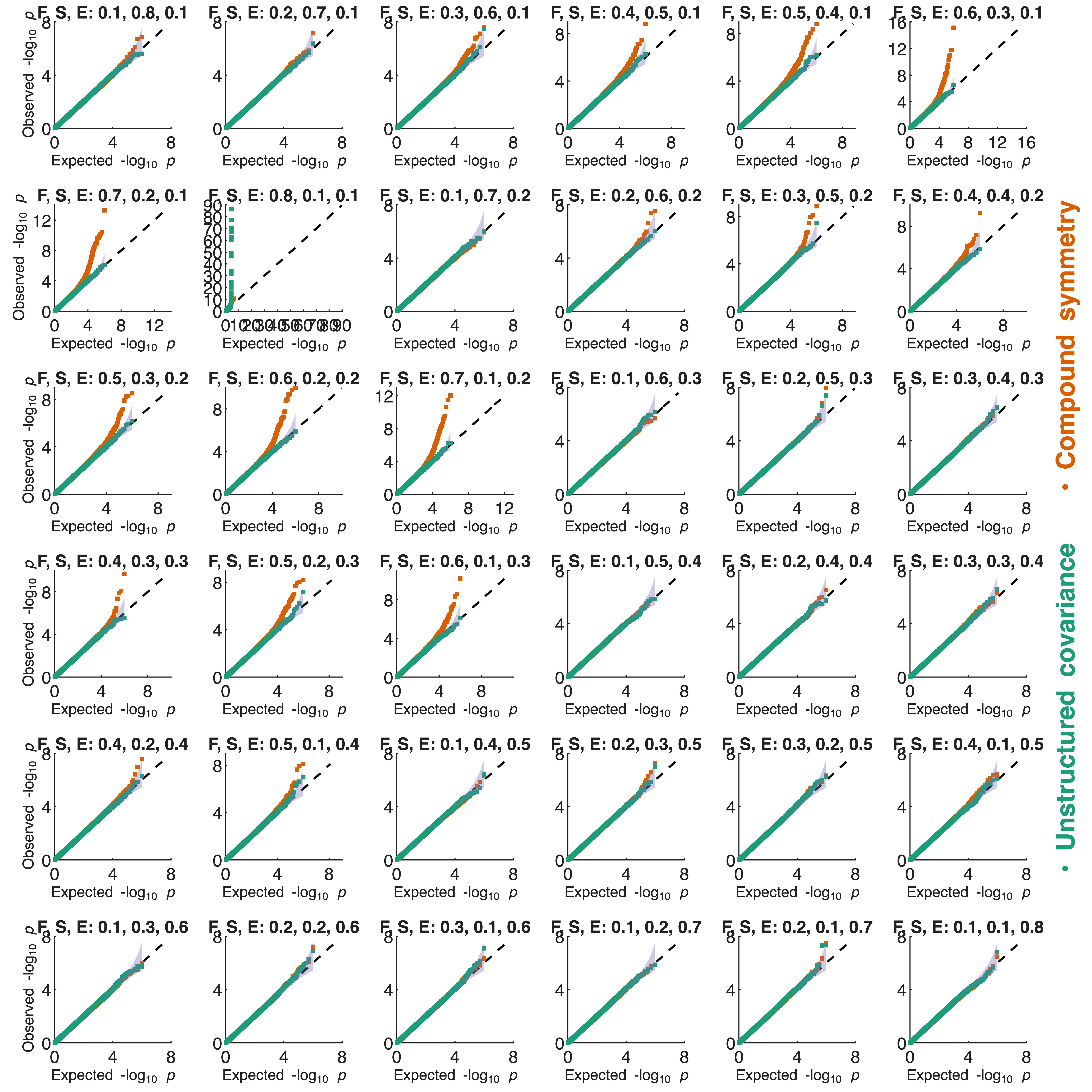

Supplement: S31 Fig — The simulation setting is indicated on the top of each Q-Q plot indicating the amounts of variances (in the phenotype) explained by family (F), subject (S), and noise (E); the x-axes indicate the expected −log10(p) values under the null hypothesis while the y-axes show the observed −log10(p) values across 1000 repeats, 100 X variables, and 10 y variables. The purple filled area indicates the 95% confidence interval based on inverse beta distribution. Note that the y-axis is truncated for the plot in second row, second column. (TIFF) [file pgen.1012184.s043.tiff]

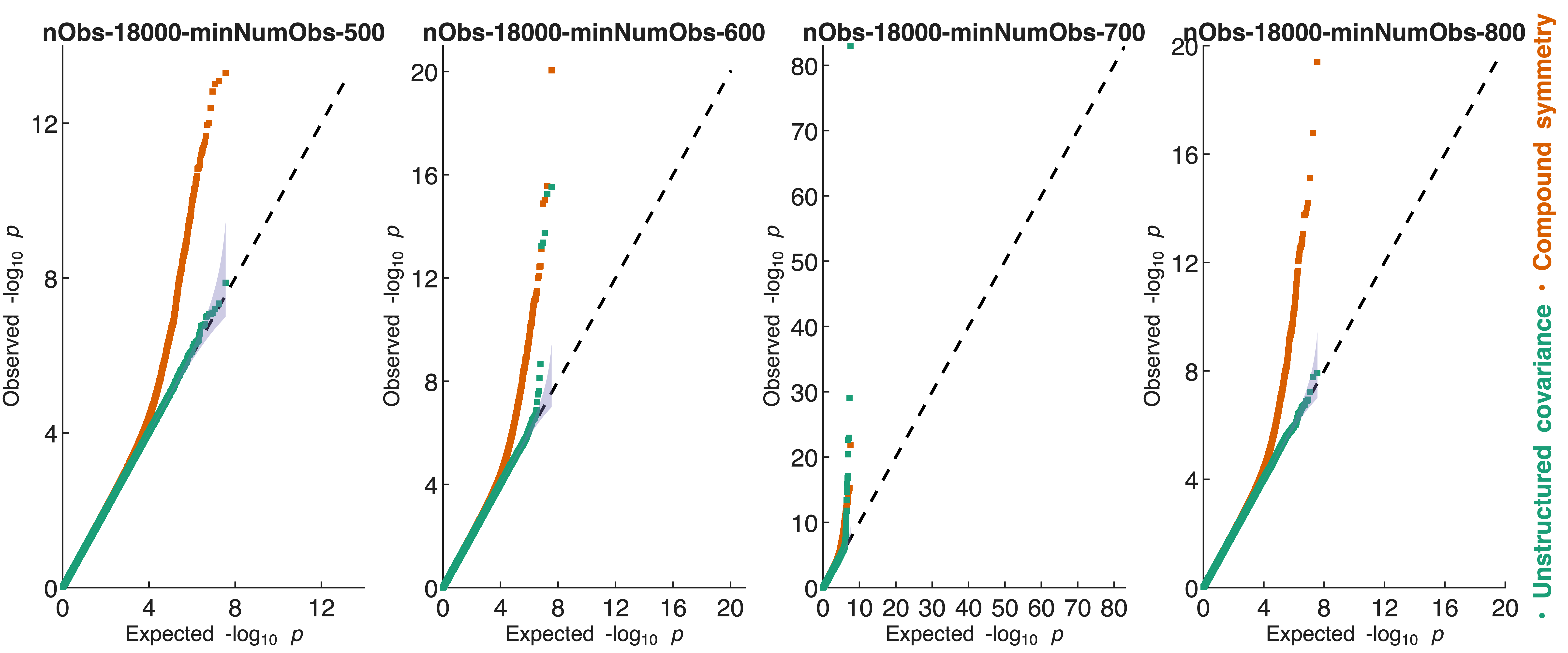

Supplement: S32 Fig — Each panel shows the distribution of −log10p-values across 1000 iterations of 36 simulation settings for unstructured covariance (green) and compound symmetry (orange); each iteration consisted of 100 X variables and 10 outcome variables; the purple filled area indicates the 95% confidence interval based on inverse beta distribution. (TIFF) [file pgen.1012184.s044.tiff]

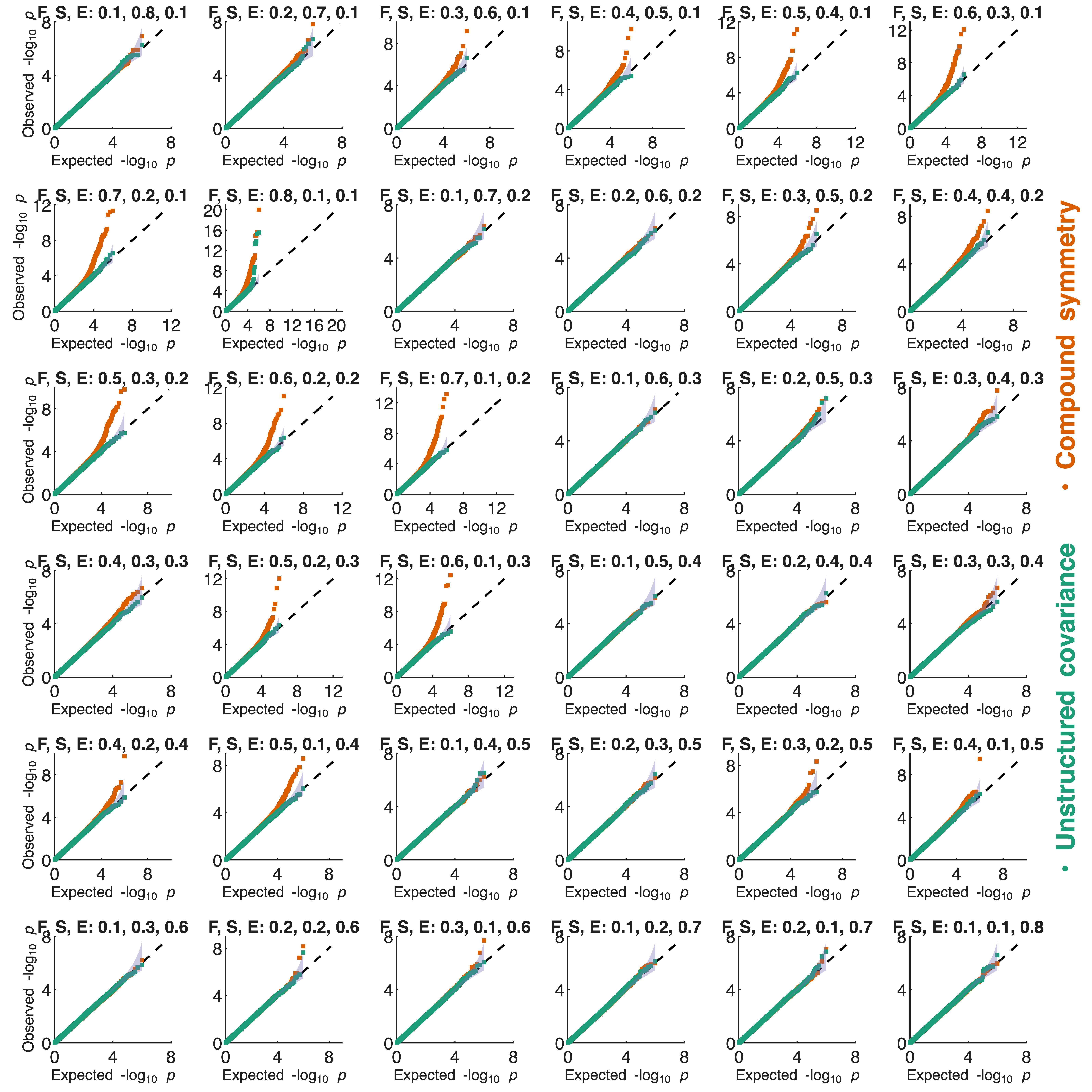

Supplement: S33 Fig — The simulation setting is indicated on the top of each Q-Q plot indicating the amounts of variances (in the phenotype) explained by family (F), subject (S), and noise (E); the x-axes indicate the expected −log10(p) values under the null hypothesis while the y-axes show the observed −log10(p) values across 1000 repeats, 100 X variables, and 10 y variables. The purple filled area indicates the 95% confidence interval based on inverse beta distribution. (TIFF) [file pgen.1012184.s045.tiff]

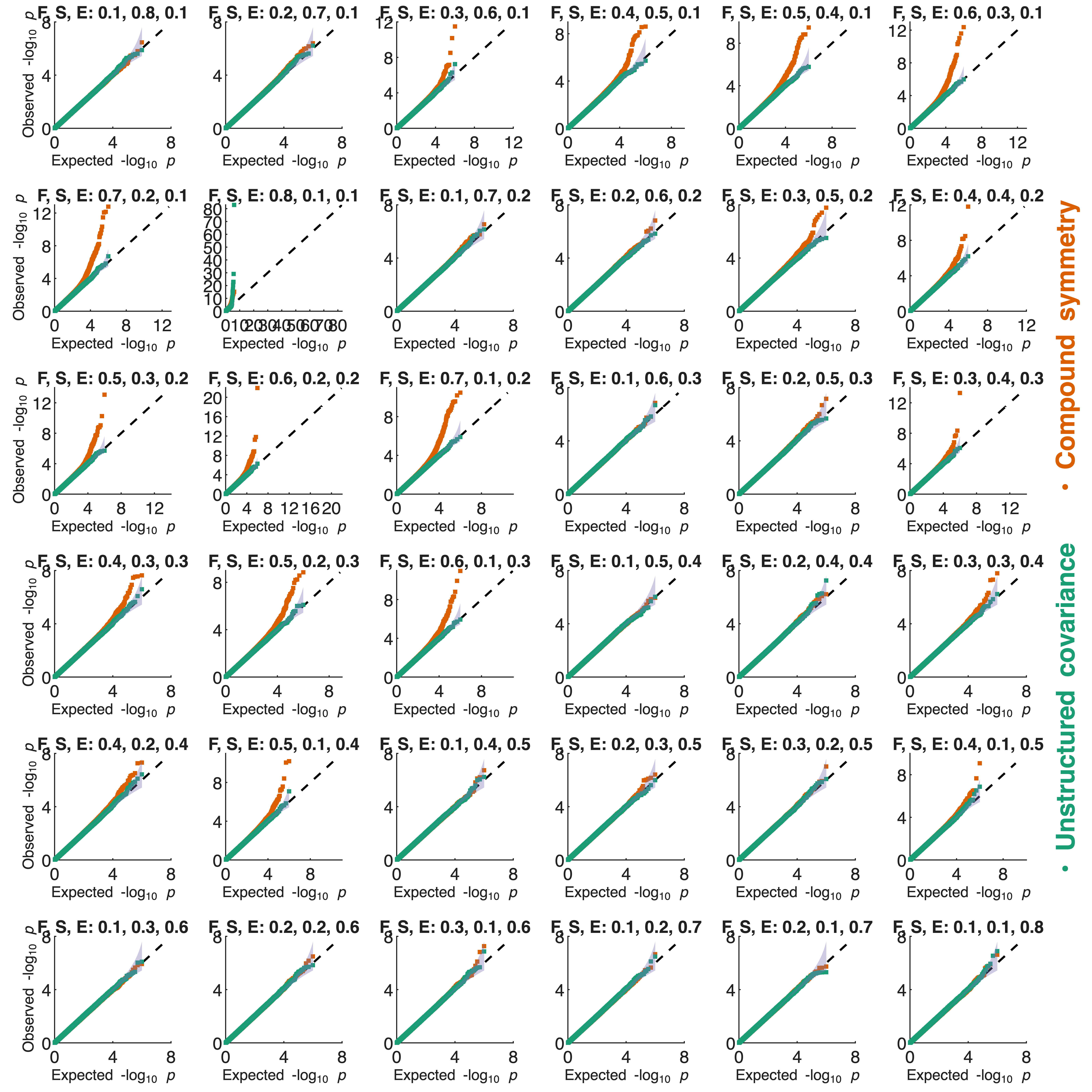

Supplement: S34 Fig — The simulation setting is indicated on the top of each Q-Q plot indicating the amounts of variances (in the phenotype) explained by family (F), subject (S), and noise (E); the x-axes indicate the expected −log10(p) values under the null hypothesis while the y-axes show the observed −log10(p) values across 1000 repeats, 100 X variables, and 10 y variables. The purple filled area indicates the 95% confidence interval based on inverse beta distribution. (TIFF) [file pgen.1012184.s046.tiff]

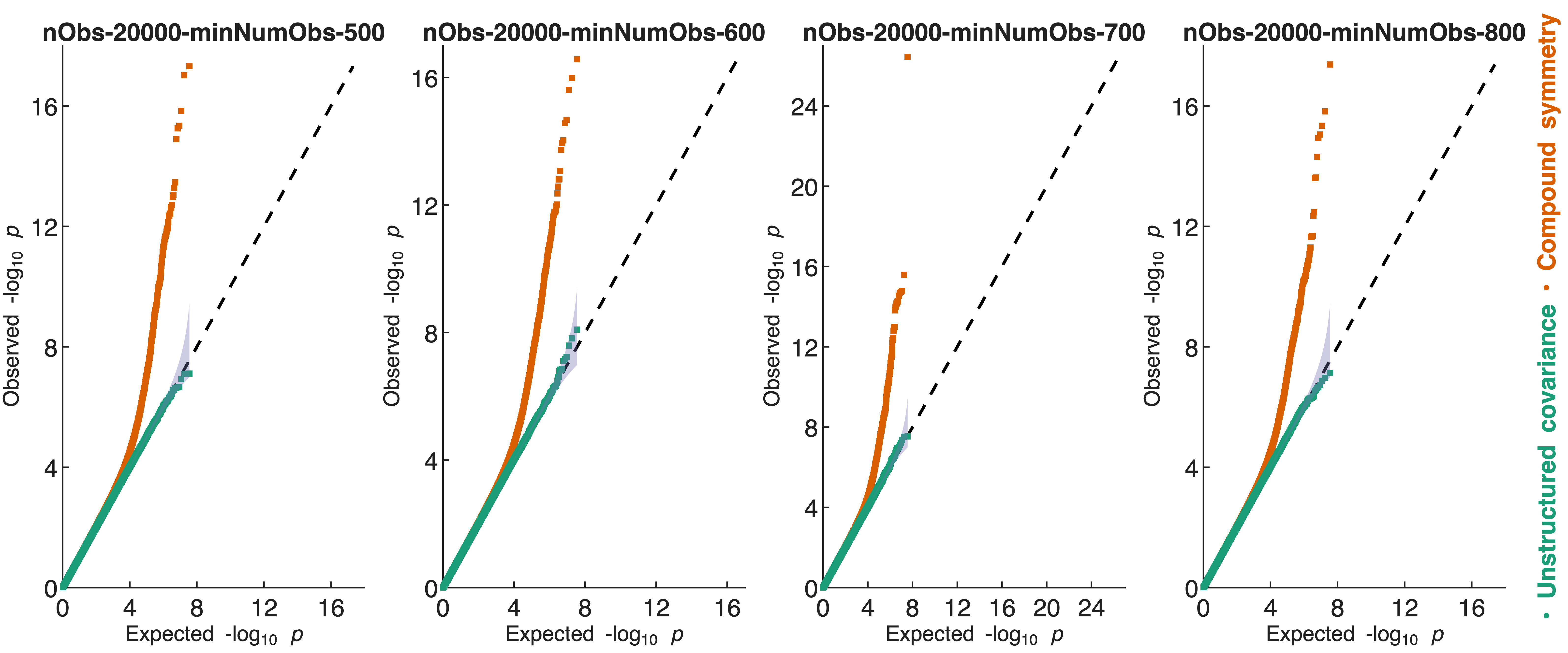

Supplement: S35 Fig — Each panel shows the distribution of −log10p-values across 1000 iterations of 36 simulation settings for unstructured covariance (green) and compound symmetry (orange); each iteration consisted of 100 X variables and 10 outcome variables; the purple filled area indicates the 95% confidence interval based on inverse beta distribution. (TIFF) [file pgen.1012184.s047.tiff]

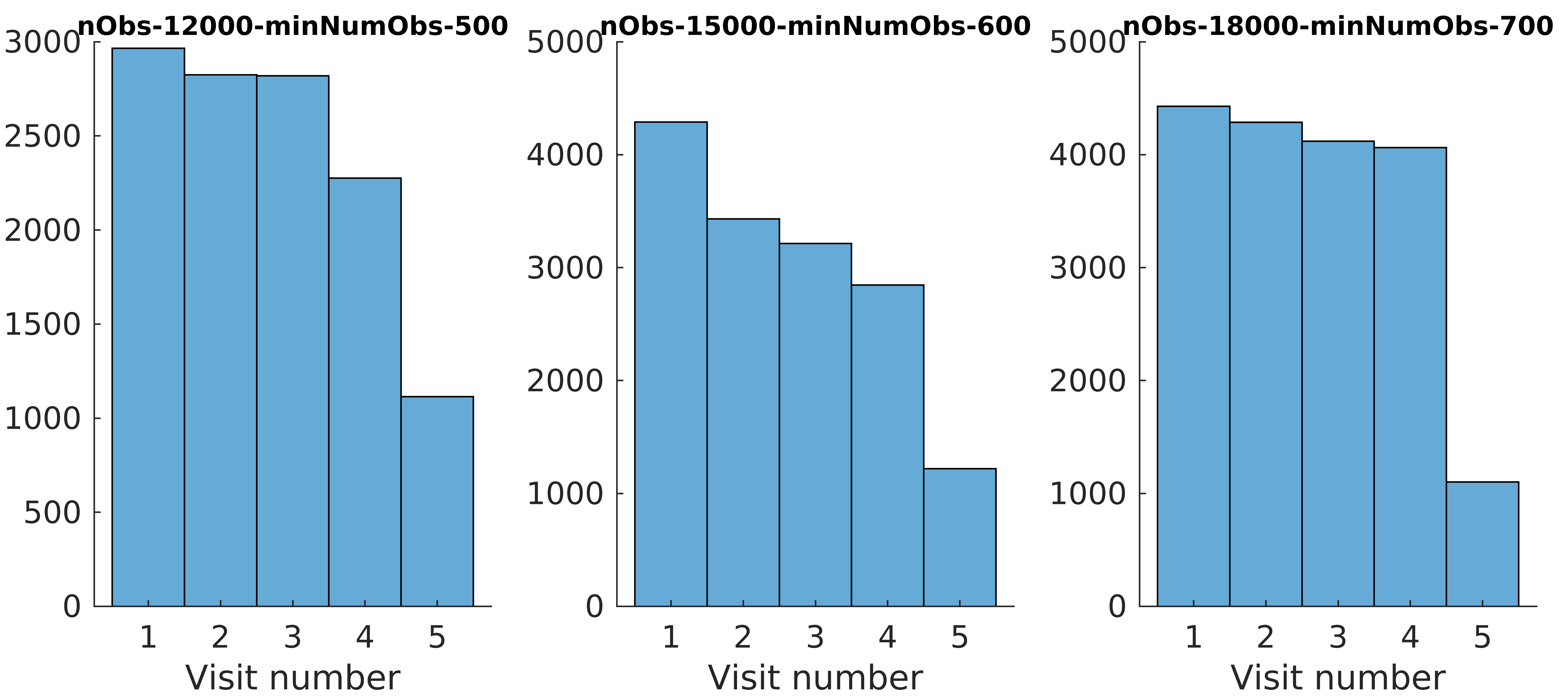

Supplement: S36 Fig — In these three examples, by chance, the simulation setting created a situation where the last visit had limited sample size; therefore, when estimating the covariance for the last pair of visits, the overlapping sample size would be close to (or equal to) minnumObs, likely leading to unstable covariance estimation, leading to inflated p-values. (TIFF) [file pgen.1012184.s048.tiff]

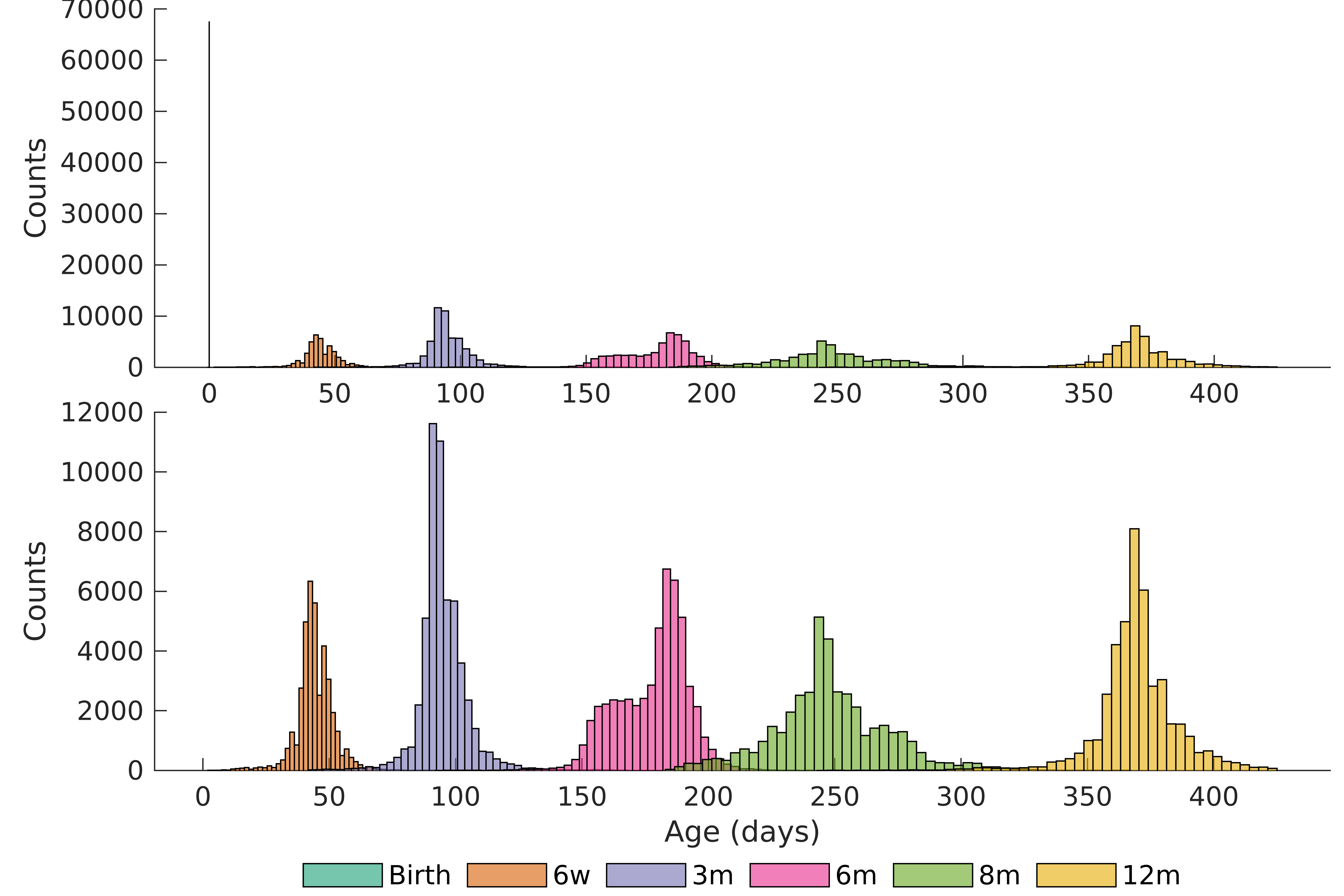

Supplement: S37 Fig — The top panel shows the histogram of age across the six time points while the lower panel shows a zoomed-in version of the same (excluding the birth time point). (TIFF) [file pgen.1012184.s049.tiff]

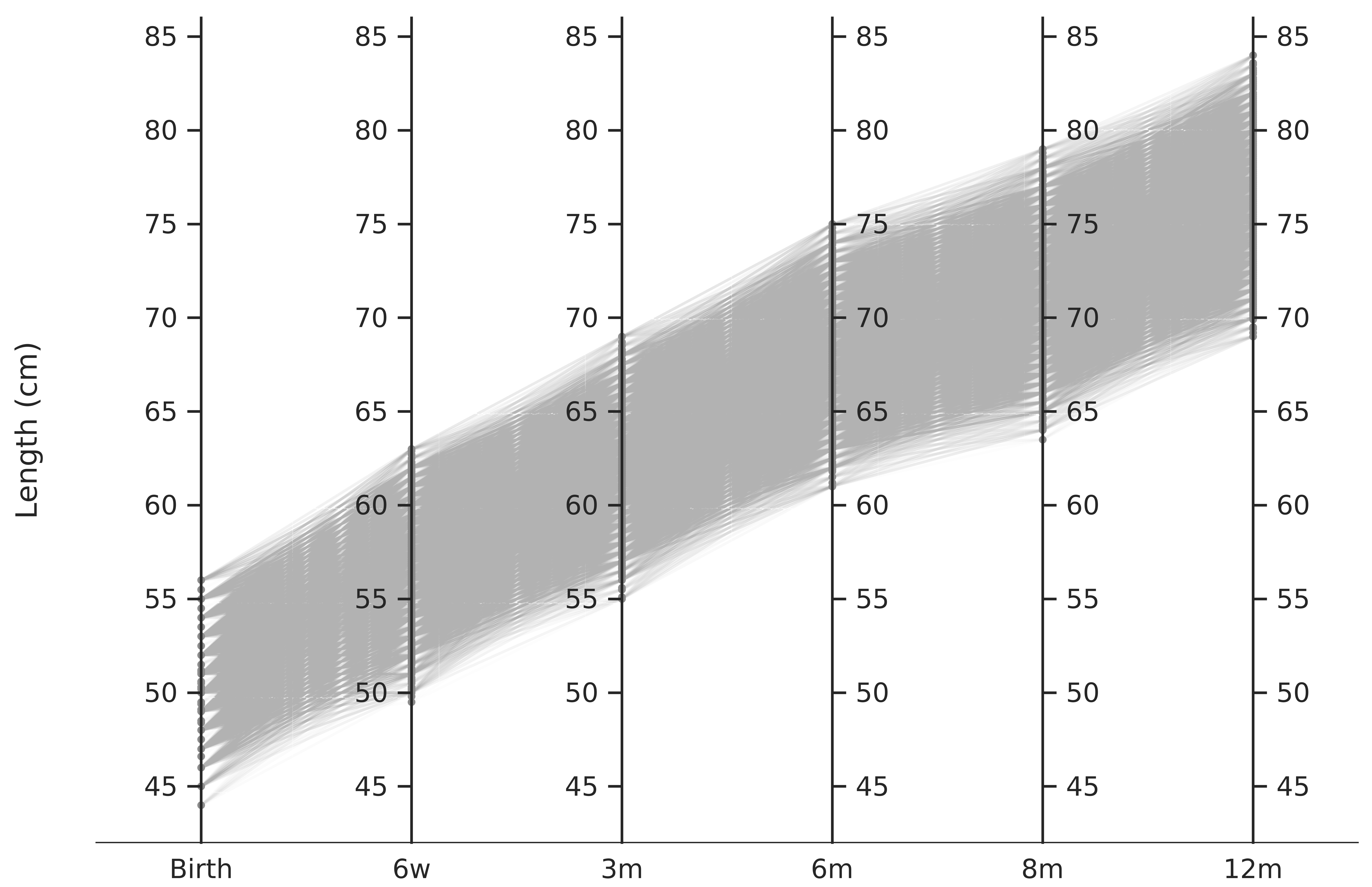

Supplement: S38 Fig — Each line shows the trajectory of length (in centimeters) from birth to the first year of life for each MoBa participant (n = 68,273 infants; 299,447 observations). (TIFF) [file pgen.1012184.s050.tiff]

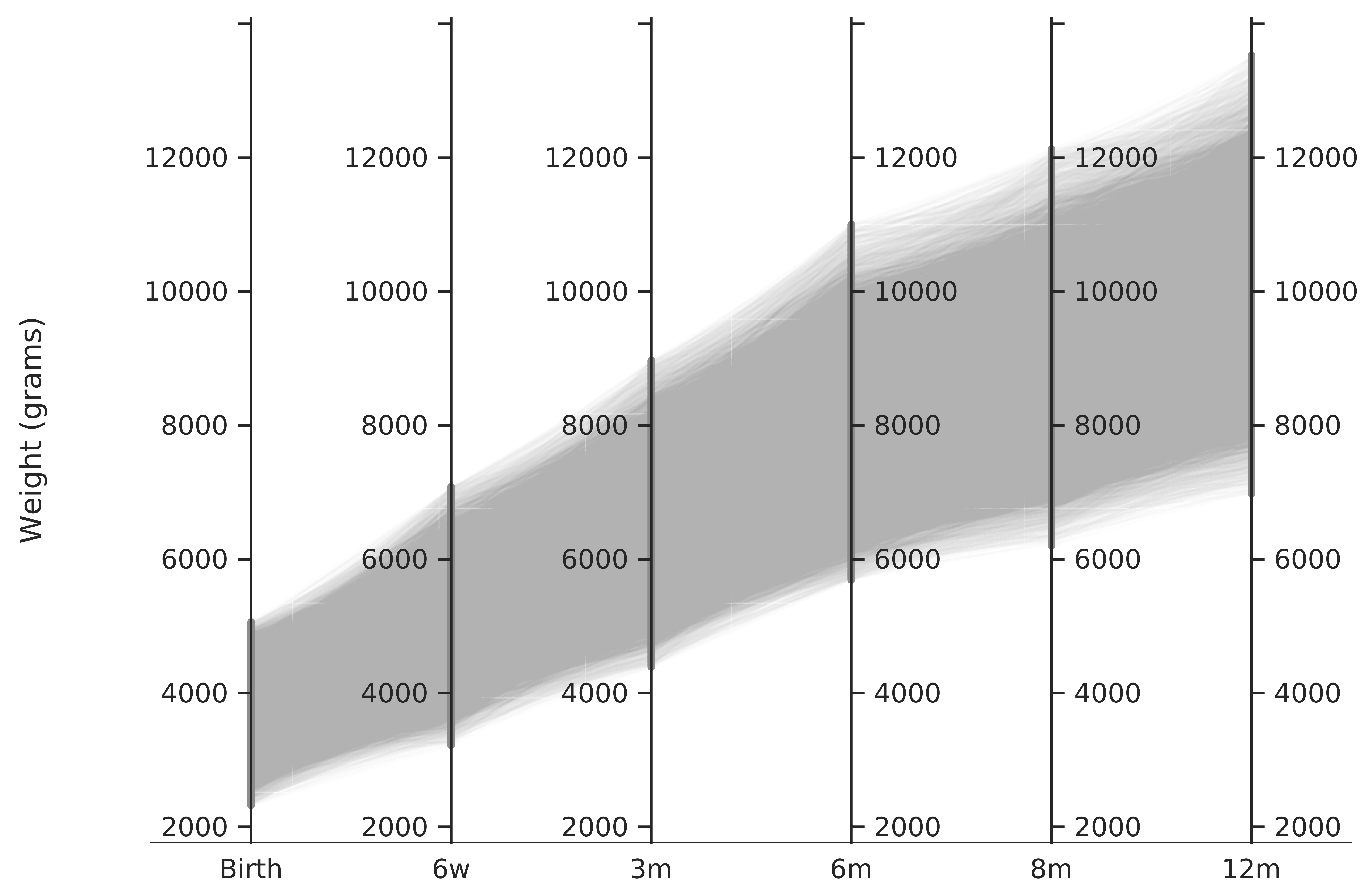

Supplement: S39 Fig — Each line shows the trajectory of weight (in grams) from birth to the first year of life for each MoBa participant (n = 68,273 infants; 299,447 observations). (TIFF) [file pgen.1012184.s051.tiff]

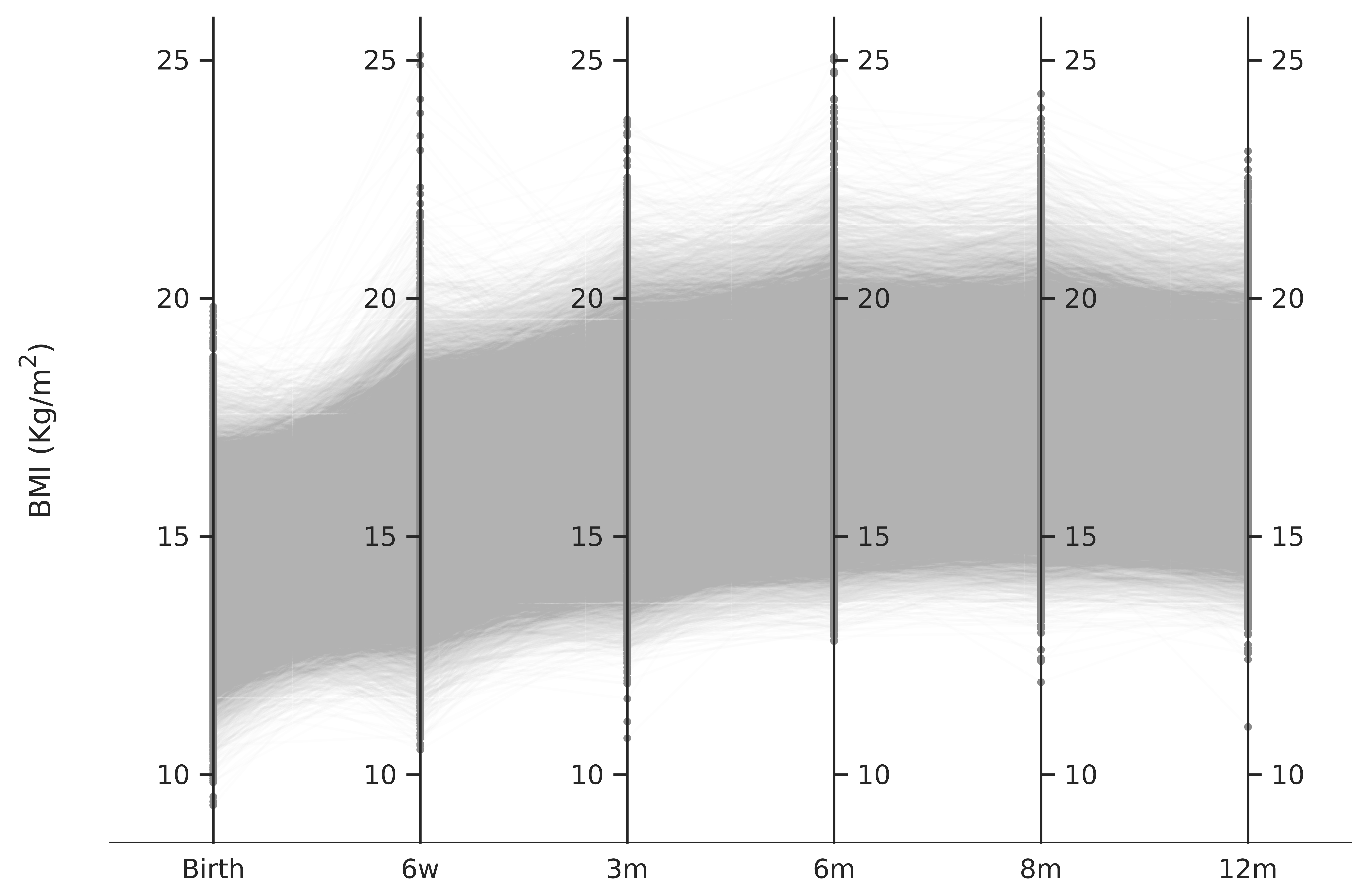

Supplement: S40 Fig — Each line shows the trajectory of BMI (kilograms per square meters) from birth to the first year of life for each MoBa participant (n = 68,273 infants; 299,447 observations). (TIFF) [file pgen.1012184.s052.tiff]

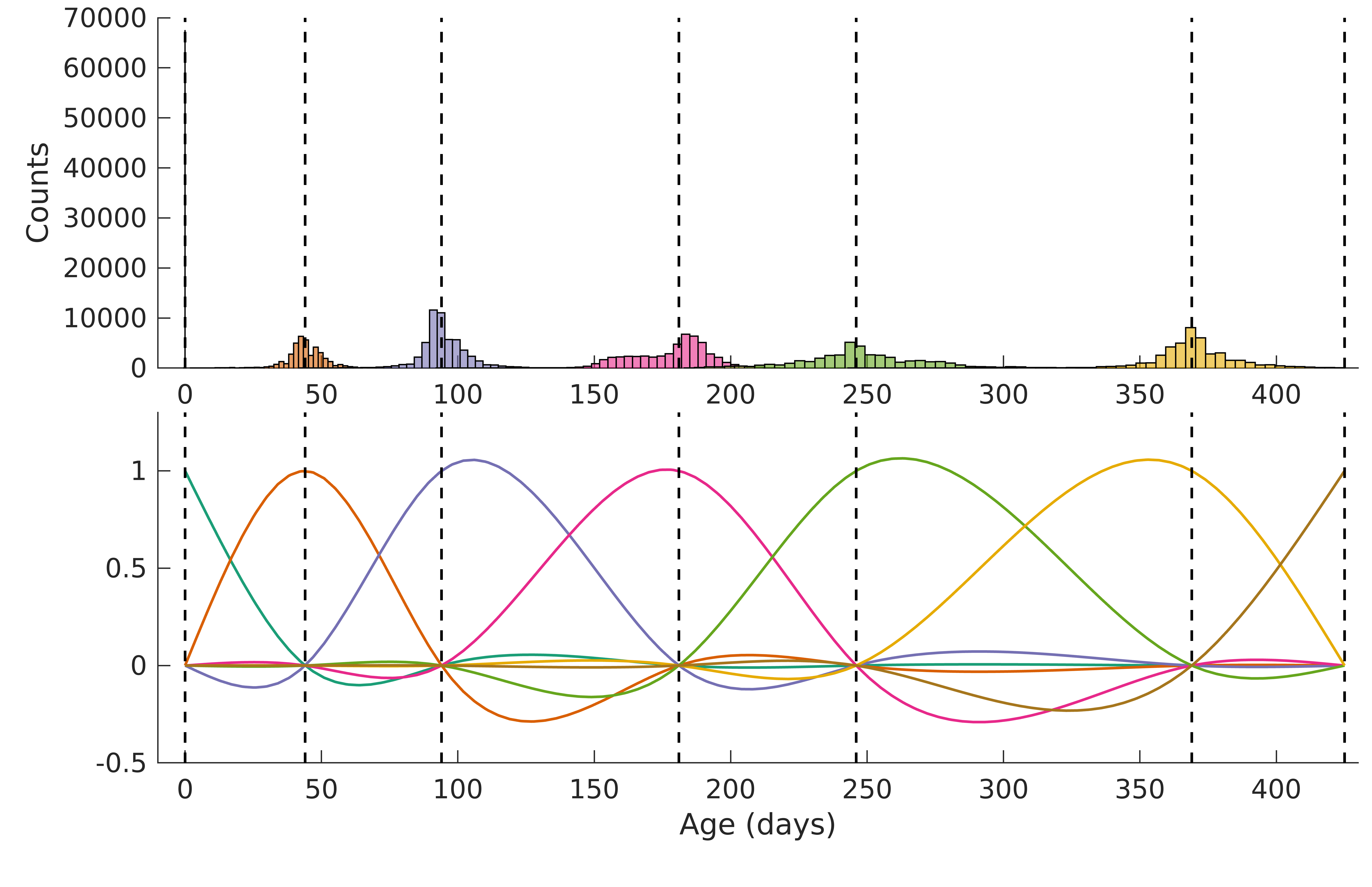

Supplement: S41 Fig — The top panel shows the histogram of age (in days) while the lower panel shows the created spline basis functions of age; the dashed vertical black lines indicate the placement of knots. (TIFF) [file pgen.1012184.s053.tiff]

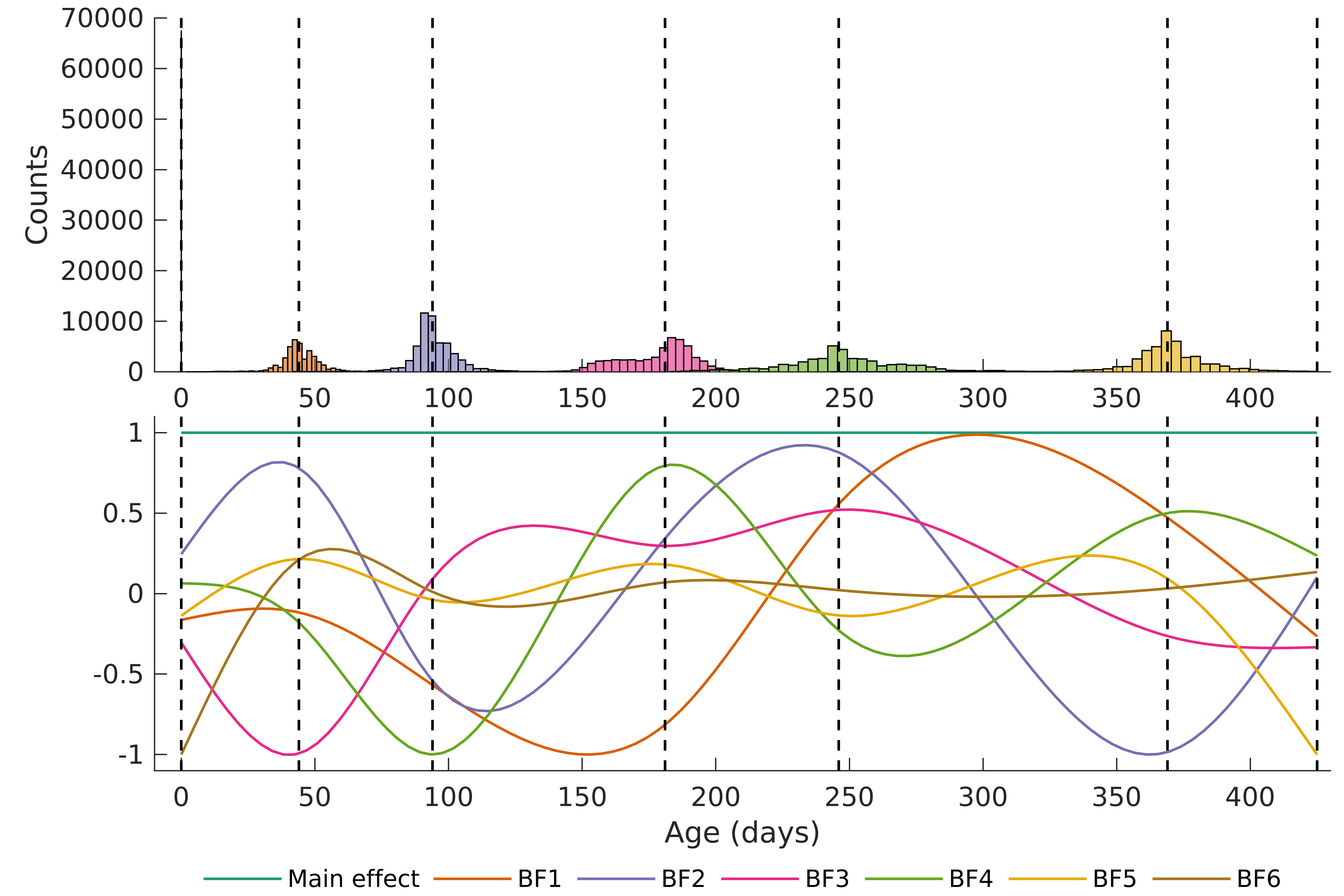

Supplement: S42 Fig — The top panel shows the histogram of age (in days) while the lower panel shows the created spline basis functions of age, followed by a singular value decomposition, and addition of the constant term; the dashed vertical black lines indicate the placement of knots. (TIFF) [file pgen.1012184.s054.tiff]

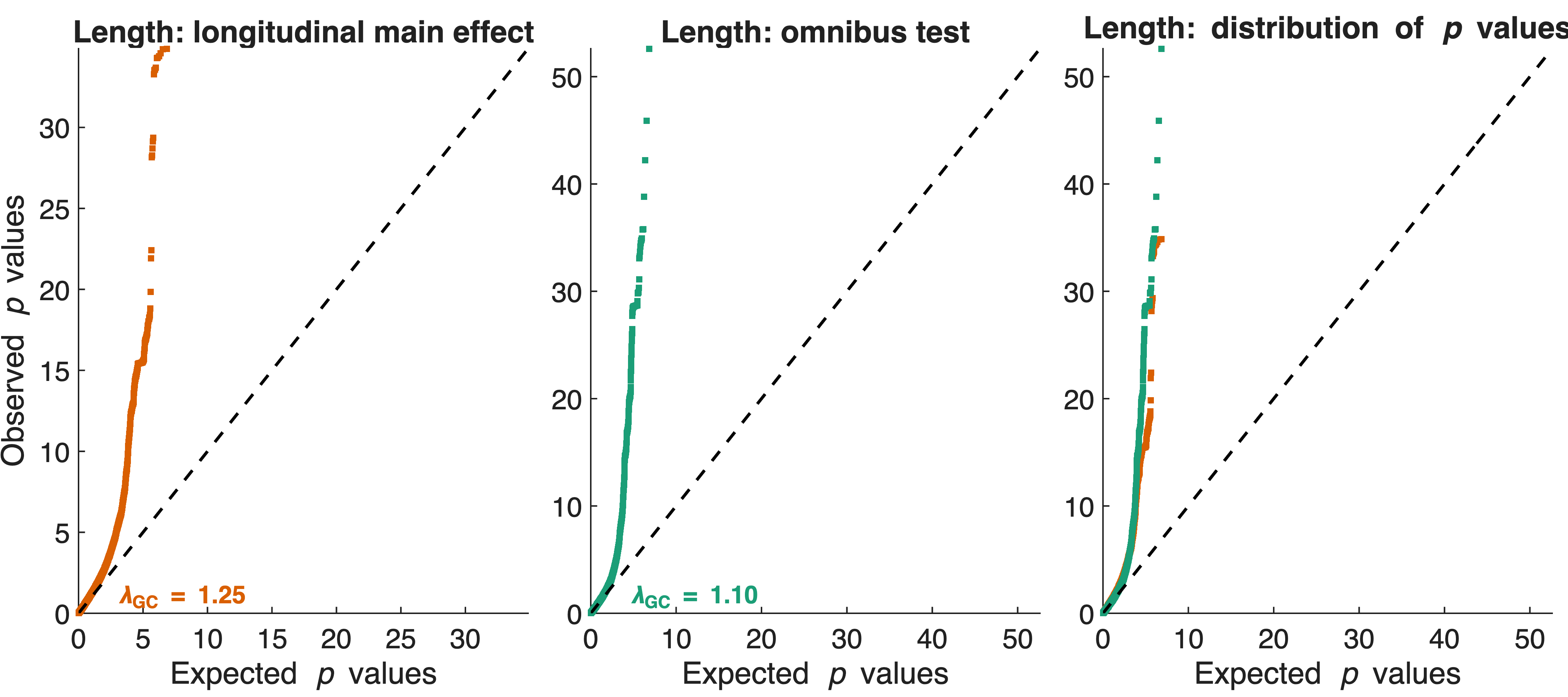

Supplement: S43 Fig — (TIFF) [file pgen.1012184.s055.tiff]

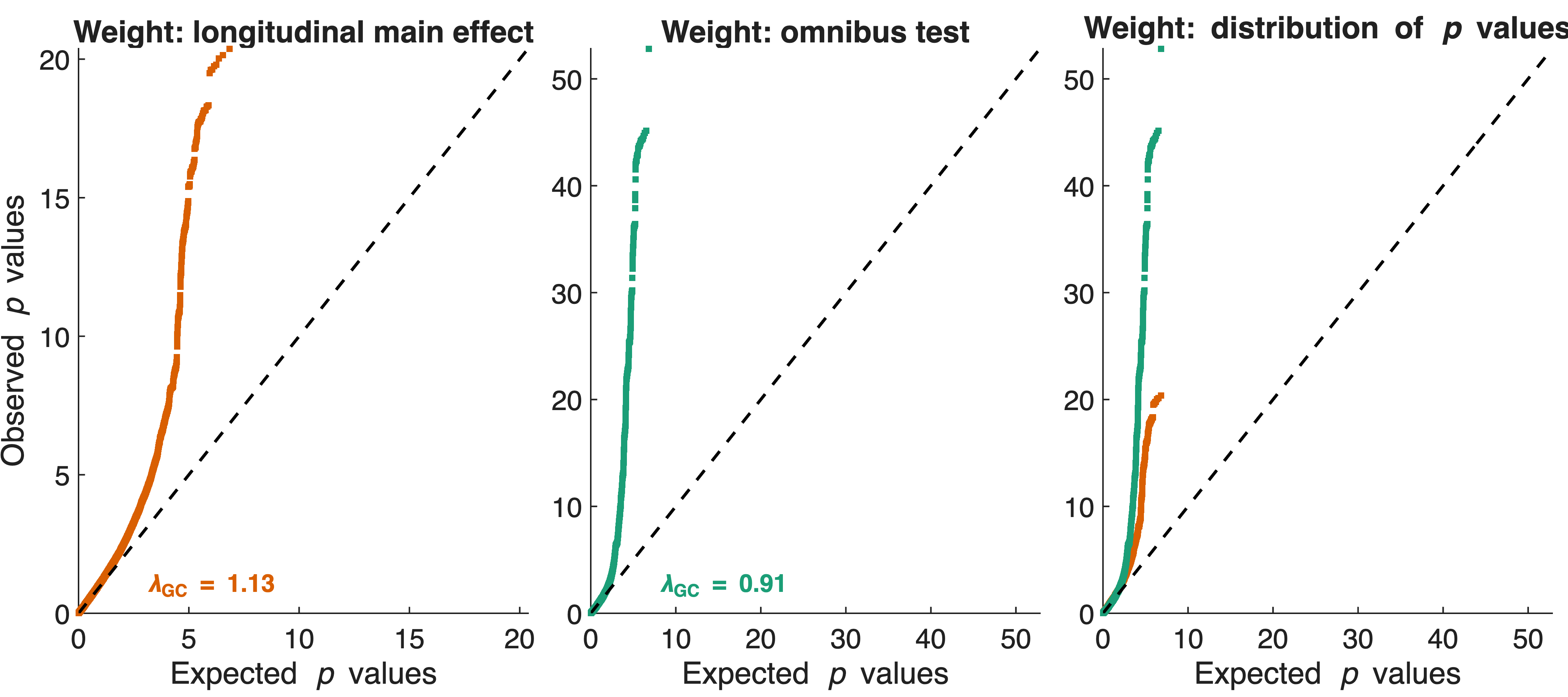

Supplement: S44 Fig — (TIFF) [file pgen.1012184.s056.tiff]

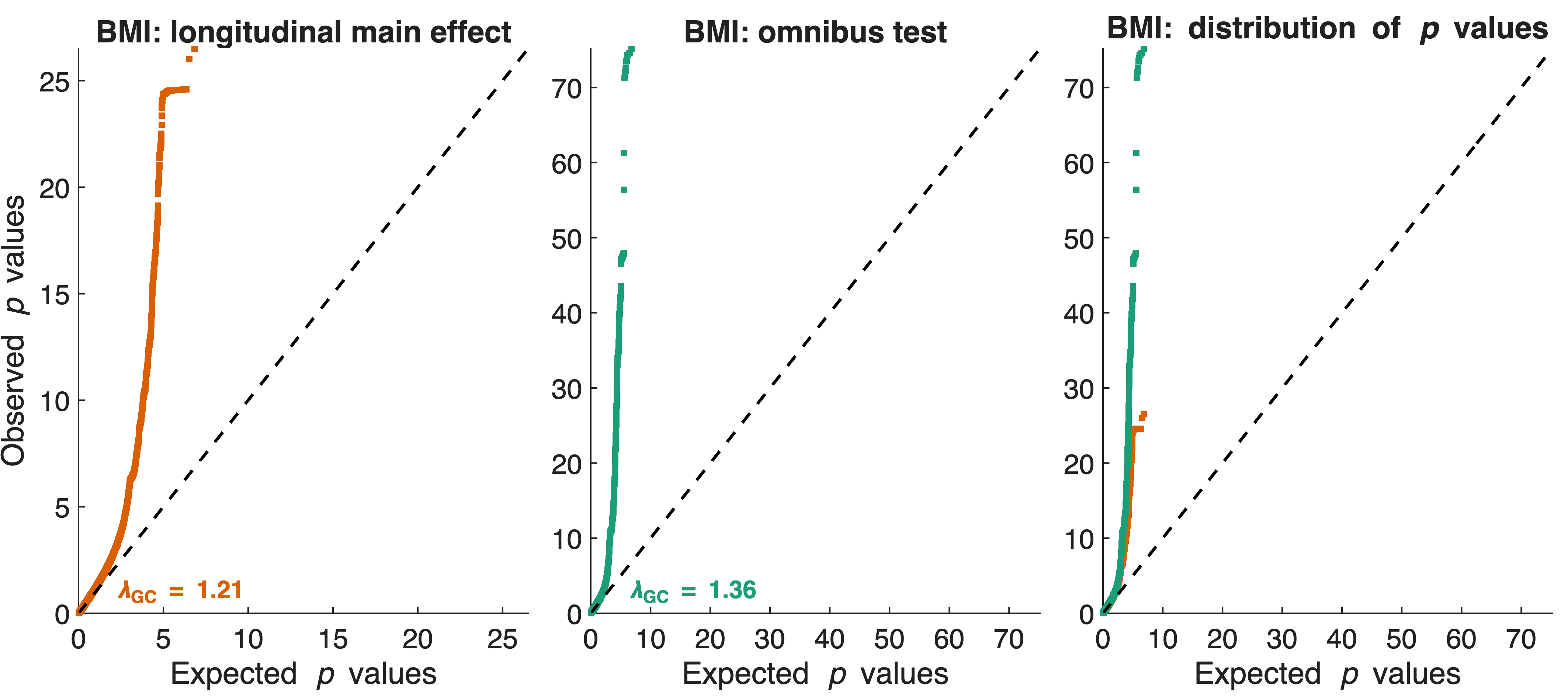

Supplement: S45 Fig — (TIFF) [file pgen.1012184.s057.tiff]

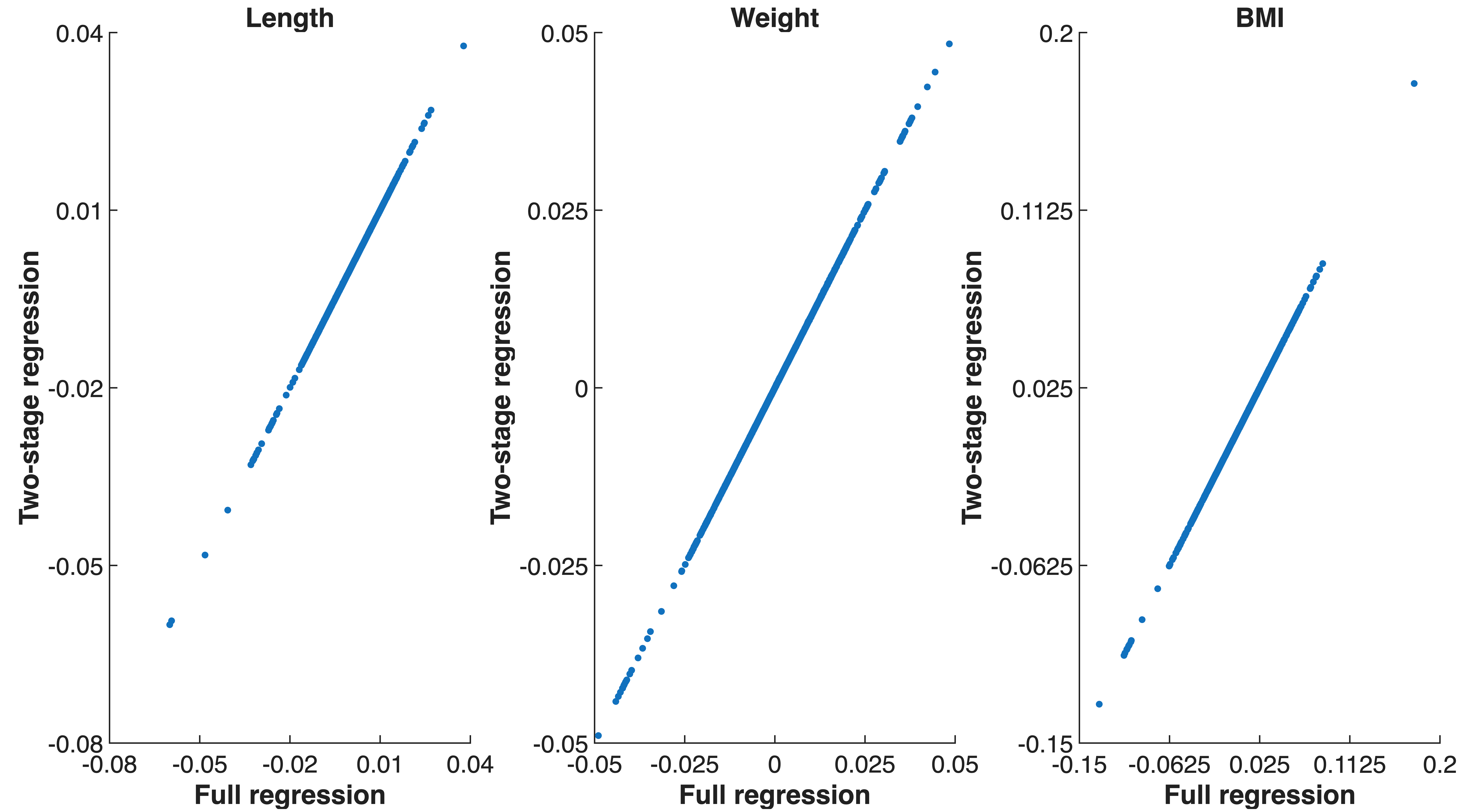

Supplement: S46 Fig — (TIFF) [file pgen.1012184.s058.tiff]

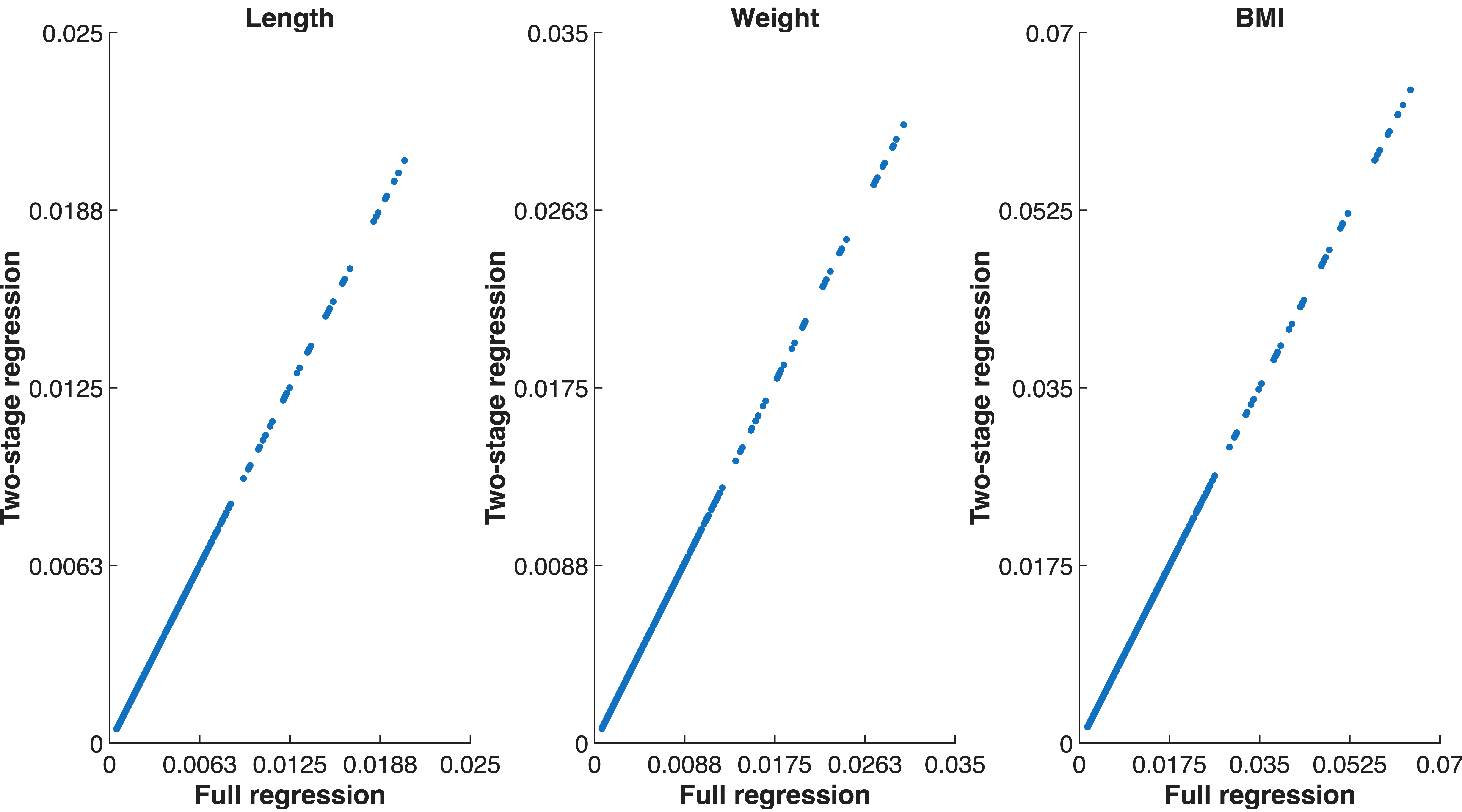

Supplement: S47 Fig — (TIFF) [file pgen.1012184.s059.tiff]

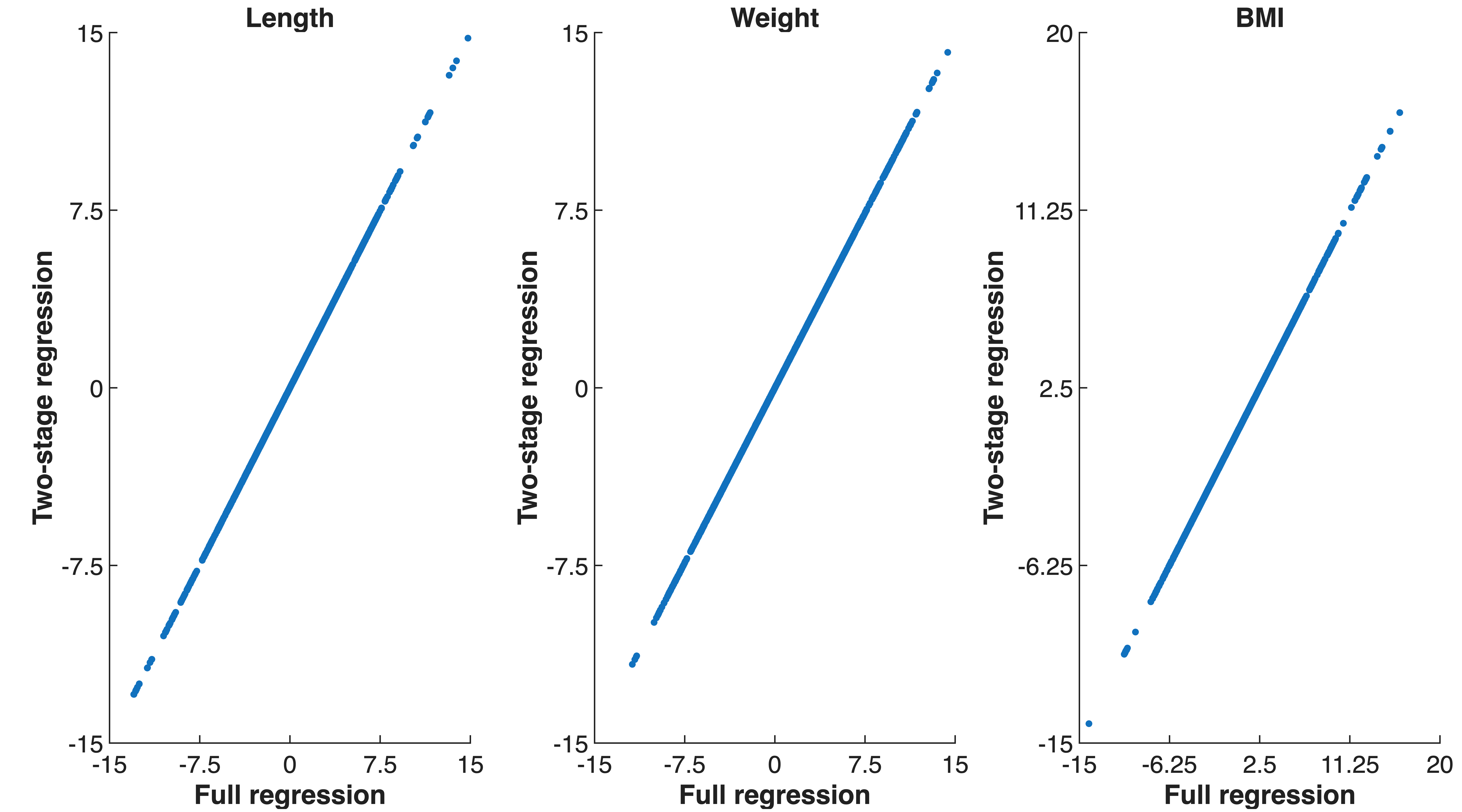

Supplement: S48 Fig — (TIFF) [file pgen.1012184.s060.tiff]

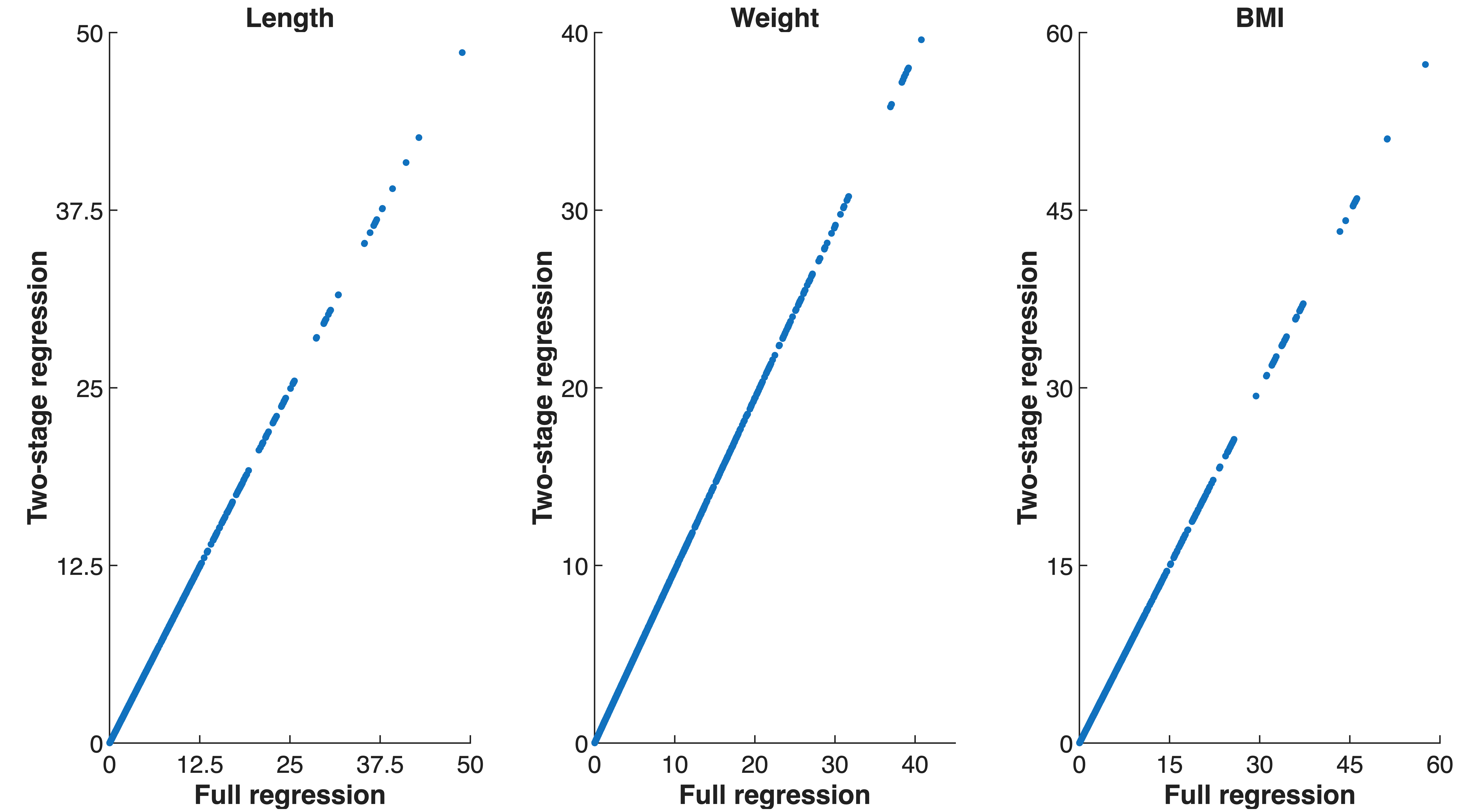

Supplement: S49 Fig — (TIFF) [file pgen.1012184.s061.tiff]

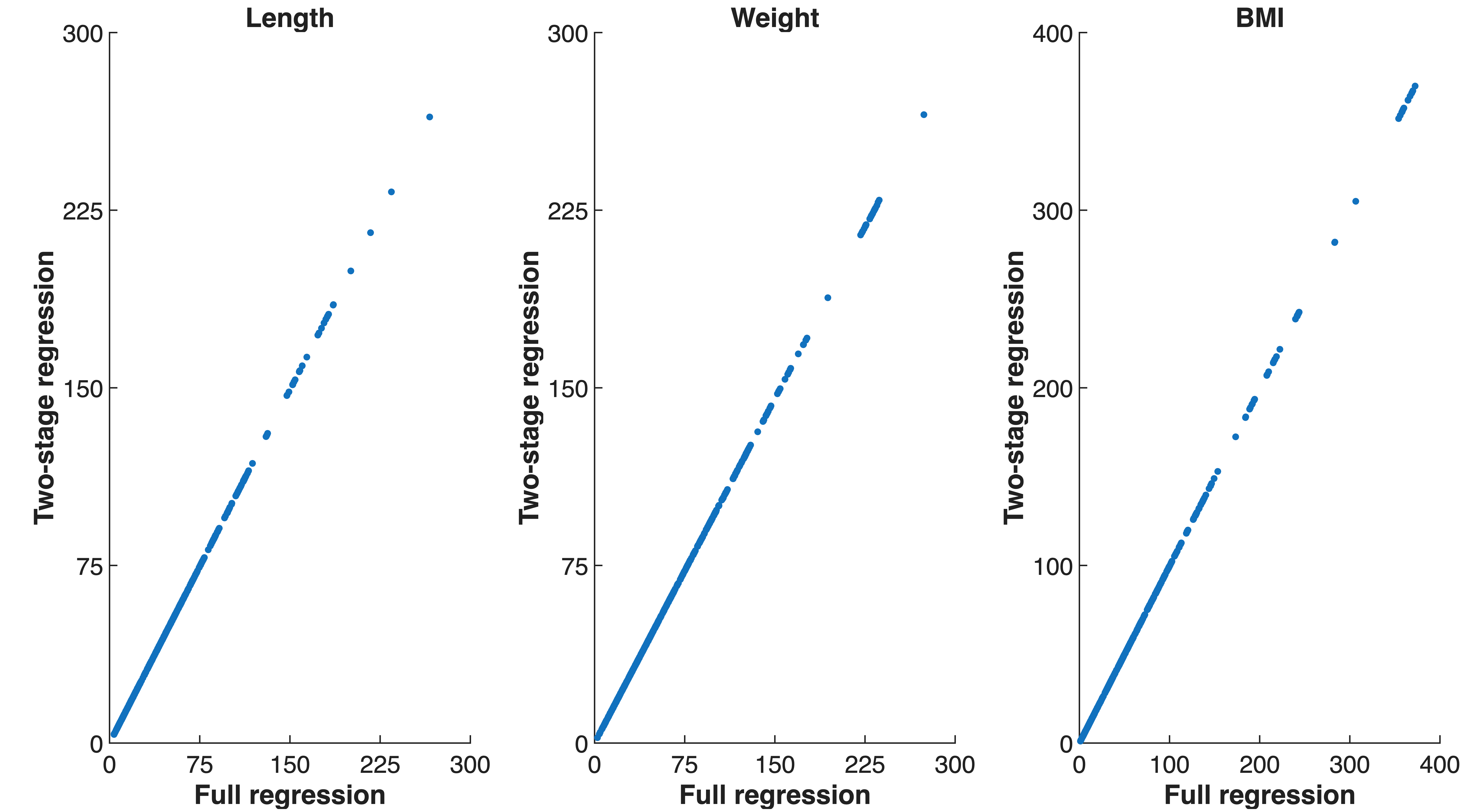

Supplement: S50 Fig — (TIFF) [file pgen.1012184.s062.tiff]

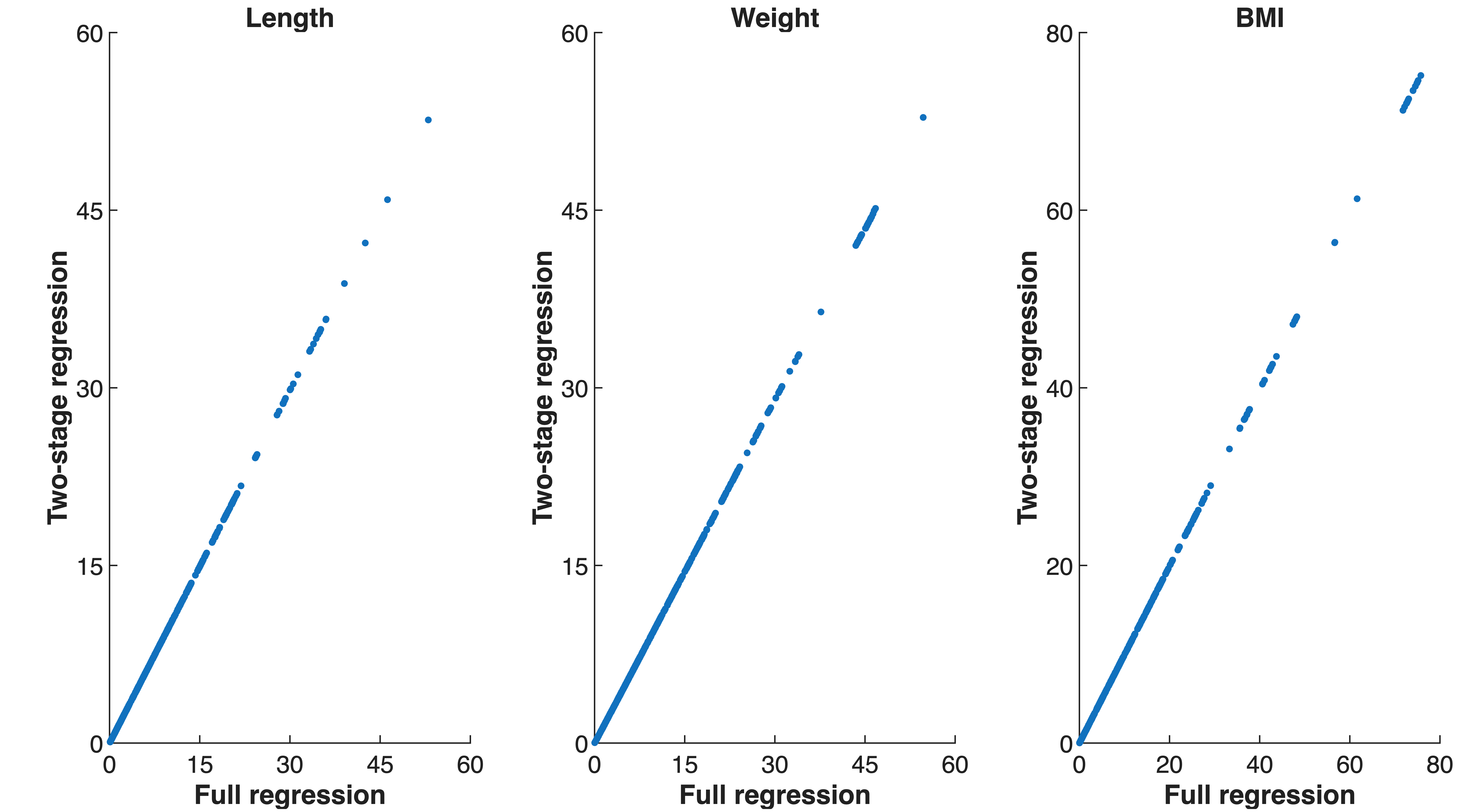

Supplement: S51 Fig — (TIFF) [file pgen.1012184.s063.tiff]

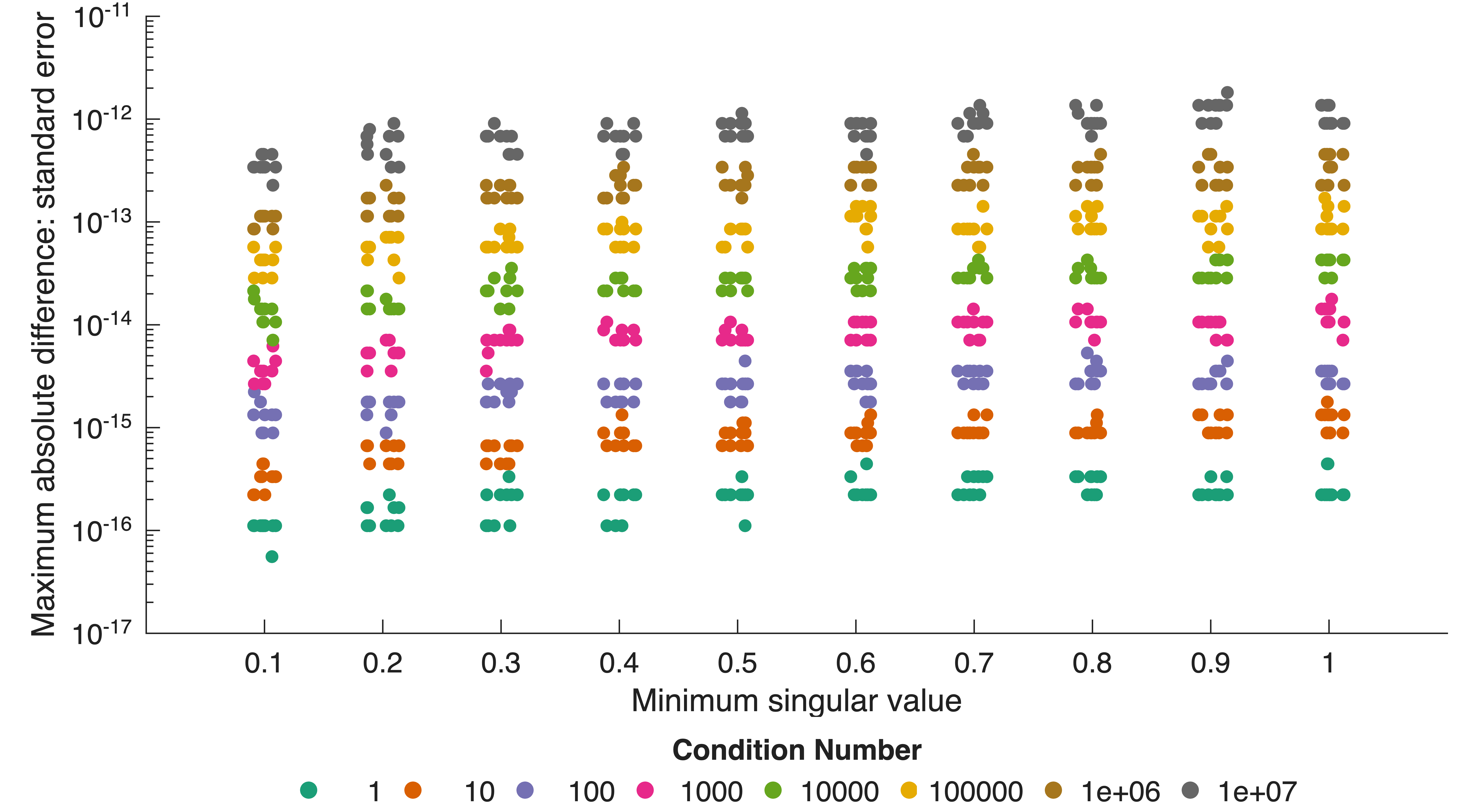

Supplement: S52 Fig — (TIFF) [file pgen.1012184.s064.tiff]
