# Supplementary material for: FEMA-Long: Modeling unstructured covariances for discovery of time-dependent effects in large-scale longitudinal datasets
Source: PLoS Genet. 2026 Jun 11;22(6):e1012184. doi: 10.1371/journal.pgen.1012184 (PMC13286282; doi:10.1371/journal.pgen.1012184)
Supplement: Attachment — Submitted filename: Reviews.pdf [file pgen.1012184.s065.pdf]

Review for "*FEMA-Long: Modeling unstructured covariances for discovery of time-dependent effects in large-scale longitudinal datasets*"

Summary of the paper

This manuscript introduces *FEMA-Long*, a scalable linear mixed model (LMM) framework for large-scale longitudinal genetic analyses with repeated measurements per individual. A central methodological contribution is to model the residual covariance as a linear combination of positive semidefinite matrices capturing multiple dependence sources and to estimate the associated variance components via a moment-based (MoM) procedure. Importantly, FEMA-Long further extends the modeling flexibility by incorporating spline basis expansions to capture non-linear fixed effects over continuous time. This approach avoids iterative likelihood-based fitting and yields a highly computationally efficient pipeline that is particularly appealing for analyzing large longitudinal datasets.

Questions for the authors

1. In Equation (3), the residual covariance is decomposed as a linear combination of known PSD matrices (e.g., $Z_F Z_F^\top$, $Z_A Z_A^\top$, $Z_S Z_S^\top$, and $\mathbf{I}$). When would the parameters be identifiable? For example, if $Z_F Z_F^\top = \mathbf{I}$, then σ_F^2 and σ_ε^2 would not be identifiable. More discussion on the identifiability conditions for the identifiability of parameters would be helpful.

2. The manuscript notes that, when estimating covariances within each pair of visits, the subject and error variances cannot be uniquely identified and are therefore estimated jointly. Could the authors clarify the implications of this non-identifiability for the interpretation of corresponding variance components?

3. Could the authors further explain the source of the approximation "$\approx$" in Equation (7) more explicitly? In particular, since $\hat{y}_{\text{res}}^{\text{OLS}} = (I - P_X)y$, the residual covariance is generally $(I - P_X)V(I - P_X)$ rather than V. Could the authors clarify that under what assumptions it is valid to approximate $(I - P_X)V(I - P_X)$ by V in Equation (7)?

4. In Equation (11), the unstructured covariance is estimated entrywise. However, there seems no constraint on the off-diagonal entries. From this perspective, would the estimated unstructured covariance be not non-negative definite? Do the authors apply any approach to guarantee validity and numerical stability for the solution?

5. Are there scenarios where the local system in Equation (11) becomes ill-conditioned due to limited or highly unbalanced numbers of observations for certain visit-pairs? For example, late-term visits often have high attrition. How does the implementation handle visit-pairs where the number of overlapping samples is insufficient to reliably solve the moment equations?

6. In Simulations 1–2, the data-generating design appears to be essentially fixed (e.g., Simulation 1 and Simulation 2 uses 5,000 unique individuals with up to 5 repeated measurements and 20,000 observations). Could the authors please add a sensitivity analysis that varies the sample size in Simulations 1–2 to assess how the statistical performance of the proposed estimation depends on sample size?
