# Supplementary material for: FEMA-Long: Modeling unstructured covariances for discovery of time-dependent effects in large-scale longitudinal datasets
Source: PLoS Genet. 2026 Jun 11;22(6):e1012184. doi: 10.1371/journal.pgen.1012184 (PMC13286282; doi:10.1371/journal.pgen.1012184)
Supplement: Attachment — Submitted filename: Review_R2.pdf [file pgen.1012184.s067.pdf]

Review for "*FEMA-Long: Modeling unstructured covariances for discovery of time-dependent effects in large-scale longitudinal datasets*"

I thank the authors for the substantial revision and the detailed response. The revision has addressed my major concerns. I only have a few comments remaining.

Questions for the authors

1. After re-reading Equations (3)–(8), I still have some questions about the approximation underlying Equation (7). If $i \neq i'$, i.e., for off-diagonal entries, it seems that the σ_ε^2 term should not appear on the right-hand side, since the noise component is proportional to the identity matrix (please correct me if I am misunderstanding this point).

   More generally, it is still unclear to me how the normalization factor $\hat{\sigma}^2$ should be interpreted relative to $\sigma^2 = \mathrm{tr}(V)/n$ since the covariance matrix of OLS residuals seems to be $(I - P_X)V(I - P_X)$, where $P_X = X(X^\top X)^{-1}X^\top$ (again please correct me if I am misunderstanding this point). Relatedly, one might expect that $\mathbb{E}(\hat{\sigma}^2) = \frac{1}{n-p} \mathrm{tr}\left[(I - P_X)V(I - P_X)\right]$, which is associated with a projected residual-scale quantity rather than directly with $\mathrm{tr}(V)/n$.

   I also wonder whether this step could be clarified by writing the expectation explicitly on the residual product in the numerator, with normalization by σ^2 or the estimator $\hat{\sigma}^2$ applied afterward. Additional technical details would make this part of the model much easier to follow.

2. The clarification that a nearest symmetric positive semidefinite correction is applied before computing standard errors is helpful. My remaining question is about its inferential impact. It is unclear whether this is usually a minor numerical adjustment or whether it can sometimes materially affect downstream inference. If feasible, it would be helpful to include a brief sensitivity analysis, for example reporting the size of the perturbation induced by the correction and whether it meaningfully changes standard errors, test statistics, or significance conclusions. Since the simulation setting provides access to the true

covariance structure, it may also be informative to compare covariance estimation error before and after the nearest-PSD correction.

3. The added simulations varying overall sample size and minimum overlap between visit pairs are useful. One point that may be worth stating more explicitly is what is meant here by “large sample.” In the present visit-wise covariance-estimation setting, the practically relevant notion of sample size seems to be the number of overlapping observations for each visit pair, rather than only the total number of observations. The revised simulations appear to support this interpretation, since both RMSE and calibration depend strongly on the minimum overlap across visit pairs. Making this point explicit would strengthen the practical guidance for readers.
