# Supplementary material for: FEMA-Long: Modeling unstructured covariances for discovery of time-dependent effects in large-scale longitudinal datasets
Source: PLoS Genet. 2026 Jun 11;22(6):e1012184. doi: 10.1371/journal.pgen.1012184 (PMC13286282; doi:10.1371/journal.pgen.1012184)
Supplement: Attachment — Submitted filename: FEMA-Long_R1-ResponseLetter.docx [file pgen.1012184.s066.docx]

Response to Reviewer’s Comments

We thank the Reviewers and the Editors for reading our manuscript, providing insightful comments, as well as giving us an opportunity to revise our work. Below, we provide a point-by-point response to each of the comments raised by the Reviewers. The comments by the Reviewers are in fixed-width blue text while our responses are in regular serif font in black. Additionally, when possible, we provide a comparison between the old and new manuscript with the changed content highlighted; the corresponding page and line numbers reflect the location of the change in the tracked changes version of the revised manuscript.

# Reviewer 1

In this manuscript, the authors extended their previous FEMA method to allow unstructured longitudinal covariance, multiple random effects, spline interactions, and GWAS-scale testing. The proposed algorithm is computationally efficient and addresses an important gap in longitudinal GWAS methodology. I suggest the acceptance for publication after resolving the following issues:

## Major comments:

1. Notations. I suggest using boldface consistently for vector and matrices to avoid confusion and avoid reusing the same symbols for different objects. For example, in equation 12, the coefficient of fixed effects in the first term is denoted by beta, which is a vector, and the coefficient of the effect on spline basis functions is by denoted beta_s, which is a scalar. This creates ambiguity, because beta_1 can be interpreted as either the effect of the first fixed effect or the first spline basis. In the following Omnibus Wald test (equation 13), beta is used again. But here it appears to refer only to coefficients for the spline basis functions, that is, beta_s in equation 12, because the degree of freedom is S.

Response: We thank the Reviewer for this suggestion. We have now updated all the equations and notations so that matrices and vectors are indicated with boldface font and scalar items are indicated with a non-boldface font weight.

We have updated equation 12 to clearly demarcate between the estimated coefficients for the covariates and the estimated coefficients for the basis functions; similarly, we have updated equations 21 and 22 to avoid ambiguity.

| Original Manuscript | Revised Manuscript |
| --- | --- |
| $\begin{aligned} y_{i}=X_{i}\beta+\sum_{s=1}^{S} \beta_{s}f_{s}\left( t \right)+\varepsilon_{i} \#\left( 12 \right) \end{aligned}$ | $\begin{aligned} y_{i}=\boldsymbol{X}_{i}\boldsymbol{\beta}+\sum_{s=1}^{S} {\alpha\beta}_{s}f_{s}\left( t \right)+\varepsilon_{i} \#\left( 12 \right) \end{aligned}$[Page 8, line 20] |
| Then, the set of coefficients, $\beta_{s}$, define the non-linear function $\mathcal{F}\left( t \right)=\sum_{s=1}^{S} \beta_{s}f_{s}(t)$, […] | Then, the set of coefficients, ${\alpha\beta}_{s}$, define the non-linear function $\mathcal{F}\left( t \right)=\sum_{s=1}^{S} {\alpha\beta}_{s}f_{s}(t)$, […] [Page 8, lines 21-22] |

For the Wald test, we use the beta symbol generically – the Wald test allows for hypothesis testing using a weighted combination of any set of estimated coefficients and is not confined to just the coefficients associated with the smooth terms. To clarify this, we have now added a statement:

Note that equation $\left( 13 \right)$ can be used to perform Wald tests on any combination of coefficients estimated in $\left( 12 \right)$; i.e., not just limited to the estimated spline coefficients. [Page 9, lines 15-17]

2. In the GWAS-like scaling, Vhat is estimated from a genotype-free stage-1 model and then reused for genotype residualization and SNP testing (equations 9, 15, 17). The authors are implicitly assuming that omitting g when estimating variance components introduces negligible error in Vhat, and that uncertainty in Vhat is ignored in stage-2 inference. Please clarify this assumption explicitly. It would help to add a brief justification and a sensitivity check for a few large-effect loci showing that refitting Vhat does not change inference much.

Response: Yes, the two-stage estimation assumes that excluding SNPs from the covariance estimation in stage-1 will not make a large difference. We have now made this assumption more explicit:

Note, that in this framework, we assume that the individual contribution of each SNP is small and unlikely to impact the estimation of the covariance components in stage-1 (see Supplementary Material and **Supplementary Figures S46-S51** for a comparison of the estimates between the two approaches). [Page 11, lines 7-10]

Additionally, this assumption is also mentioned in the limitations section in the Discussion:

Third, for GWAS, we assume that the individual contribution of SNPs is small and unlikely to affect the covariance estimation of the random effect. [Page 30, lines 8-9]

To examine whether the inclusion of SNPs in the estimation of the covariance parameters would impact the estimation of the SNP effect, we have now performed a supplementary analysis: for the time-varying GWAS on length, weight, and BMI of infants, we extracted the top 10 SNPs from each chromosome for each phenotype; then, for the resulting 1,110 SNPs, we fit a FEMA-Long model that included the SNP and its interaction with the smooth basis functions of age in the main model (i.e., instead of two-stage estimation, we performed a single estimation for each SNP). Then, we compared the estimated parameters with the parameters from the two-stage regression approach. The results show that the model parameters are comparable across the two approaches. The results from this supplementary analysis is included in the Supplementary Materials (**Figures S46** – **Figure S51**); two representative figures from this analysis is reproduced below for your reference (the first figure shows the scatter plots of *T*-statistics (beta/SE) for the main effect and the SNP interaction terms across the 1,110 SNPs for length, weight, and BMI; the second figure shows the omnibus $-\log_{10} \text{Wald }p$ values for the same:

**Figure S48**: Comparison of *T* statistics (main effect and the interaction effects of SNPs with smooth basis functions of age) between full regression (where all terms were estimated together) vs. two-stage regression (where the stage-1 regression and the estimation of random effects covariance terms did not include genetics).

**Figure S51**: Comparison of $-\log_{10} \text{Wald }p$ (across the main effect and the interaction effects of SNPs with smooth basis functions of age) between full regression (where all terms were estimated together) vs. two-stage regression (where the stage-1 regression and the estimation of random effects covariance terms did not include genetics).

The above analyses suggest that the two approaches yield comparable estimates. Note that we have not reported a comparison of the total residual variance and the covariance parameters since these are only estimated once in case of two-stage regression.

3. The proposed algorithm estimates unstructured visit-by-visit covariance matrices using pairwise method-of-moments estimates (equation 11), with each covariance term estimated independently. This approach does not guarantee that the assembled covariance matrix is positive semidefinite. Since the GLS inference requires inversion of Vhat, please clarify whether any regularization, eigenvalue correction, or nearest positive-definite projection is applied in practice. If not, a short discussion of numerical stability and why this issue does not materially affect the simulations or GWAS results would be helpful.

Response: Yes, the pairwise method-of-moments estimates do not guarantee that the resulting variance-covariance matrix is positive semidefinite. Following the variance-covariance matrix estimation, when computing the standard errors, we use the nearest symmetric positive semidefinite algorithm to ensure that the inverse of $\hat{\boldsymbol{V}}$ is positive semidefinite. On rare occasions, it is possible that the positive semidefinite step does not converge (i.e., the algorithm is caught in an infinite loop); therefore, we limit the number of iterations of trying the nearest positive semidefinite algorithm to 100; if the result is not a positive semidefinite matrix, we generate a convergence warning for that SNP. We have now added a statement to clarify this in the manuscript:

When computing the standard errors using equation $(10)$, we use a nearest symmetric positive semidefinite algorithm (based on (Higham, 1988) and implemented in (D’Errico, n.d.)) to ensure that the covariance matrix is positive semidefinite; if this process fails, we generate a convergence warning message. [Page 8, lines 13-16]

There are other ways we could potentially improve this estimation process. For example, we could consider performing a joint estimation of all components of the covariance matrix such as by following a log Cholesky decomposition approach; we have mentioned this as a future direction:

Fifth, when performing the visit-wise estimation of the covariance pattern, for certain scenarios where the number of shared observations between the visits are too low, the method of moments estimation could be unstable. While FEMA and FEMA-Long are designed for large samples, we envision improving the stability of the covariance estimation by updating our method to perform a joint estimation of all parameters instead of visit-wise estimation; other possibilities include introducing a shrinkage or regularization on the estimation of the covariance parameters and/or enforcing smoothness during the estimation; additionally, we could interpolate the visit-wise estimated covariances, resulting in smoothly changing covariances and further extending the scope of time-varying random effects. [Page 30, lines 10-19]

References:

D’Errico, J., n.d. nearestSPD.

Higham, N.J., 1988. Computing a nearest symmetric positive semidefinite matrix. Linear Algebra and its Applications 103, 103–118. <https://doi.org/https://doi.org/10.1016/0024-3795(88)90223-6>

4. Pairwise estimation uses only observations that have both visits v and v'. Real longitudinal cohorts usually have irregular visit attendance (later visits may have fewer subjects), leading to substantially different pairwise sample sizes. As a result, some Omega_vv' are estimated from many subjects and others from very few. This may lead to unstable inverse of V_hat in GLS. Please provide justification for this approach and consider a sensitivity check in simulations that explicitly reflects uneven visit overlap.

Response: The pairwise estimation of the covariance components relies on the subset of observations which are shared between the two visits. It is, therefore, highly likely that different entries of the covariance matrices will have different sample sizes, and in many situations, the sample sizes can be substantially different as well. FEMA-Long is designed for large samples; in such cases, we expect that even if there is irregular visit attendance, there would be adequate sample size between any pair of visits to robustly estimate the covariances. In small sample situations (or in cases where the shared individuals between pairs of visits are too small), it is likely that the method of moments solution would not converge to the true value, which would lead to instability in the estimates. Based on the Reviewer’s comments, as well as two related comments from Reviewer 2 (comments 5 and 6), we have performed additional simulation analyses where we have explicitly varied the sample sizes as well as the minimum number of shared samples between pairs of visits – these are now reported in the manuscript as simulations 4 and 5 and the results are documented in the supplementary materials.

We observed that with reasonably large sample sizes, the estimates from FEMA-Long were stable (**Supplementary Figures S10** to **S25**; **Supplementary Tables S7** and **S8**); as the sample sizes (and the minimum number of shared observations) increased, the RMSE between ground truth and the estimates from FEMA-Long reduced. When we examined the false positive calibration for varied sample sizes and differing shared sample sizes (**Supplementary Figures S26** to **S35**), we found that in a few rare scenarios where the overlapping samples between pairs of visits became quite low, the false positive calibration was slightly off (the tails of the *p*-value distribution) in the cases where the proportion of variance explained by the random effects was large (90% of phenotypic variance attributed to random effects); examining these cases, we found that the overlapping samples between the last two visits was quite low, leading to unstable random effects estimates (**Supplementary Figures S36**):

**Figure S36**: **Three examples where the** $\boldsymbol{p}$**-values for unstructured covariance were not well calibrated**. In these three examples, by chance, the simulation setting created a situation where the last visit had limited sample size; therefore, when estimating the covariance for the last pair of visits, the overlapping sample size would be close to (or equal to) $\text{min}_{\text{numObs}}$, likely leading to unstable covariance estimation, leading to inflated $p$-values.

Taken together, these findings show that the FEMA-Long estimates for large samples are accurate and well calibrated; for smaller sample sizes (which is not the use case for FEMA-Long), we expect some instability in the estimates depending on the data (the number of clusters, the minimum overlapping sample size, the overall sample size, and how strong the random effects are).

5. Please make it clear that the proposed method is designed for only continuous, approximately Gaussian outcomes, and does not apply to binary traits.

Response: We have added this clarification in the following places:

| Original Manuscript | Revised Manuscript |
| --- | --- |
| The term *phenotype* refers to outcome variable or the dependent variable; […] | The term *phenotype* refers to a continuous outcome variable (or dependent variable) with the error in the phenotype approximately following a Gaussian distribution; […] [Page 4, lines 12-13] |
| Given a set of phenotype(s), fixed effects, and random effects, […] | Given a set of continuous phenotype(s), fixed effects, and random effects, […] [Page 4, line 23] |
| Second, currently FEMA-Long only supports continuous outcome variables, assuming normally distributed errors. | Second, currently FEMA-Long only supports continuous outcome variables, assuming approximately normally distributed errors. [Page 30, lines 6-8] |

6. In simulation 1, the description "Var ∈ [0.1, 0.9]" is ambiguous and suggests a continuous interval. The authors appear to use a discrete grid with step size 0.1, which obtains in total 84 combinations. Please clarify that variance components take values in {0.1, 0.2, …, 0.9}, or otherwise explicitly describe the discretization.

Response: Thank you for pointing out this ambiguity; we had indeed used discrete values going between 0.1 and 0.9 with increments of 0.1. We have fixed this in the revised manuscript:

| Original Manuscript | Revised Manuscript |
| --- | --- |
| We created a grid of known sources of variances: fixed effects $\text{Var}_{\text{FFX}}\in[0.1,0.9]$, family effect (i.e., variance attributed to common environment) $\text{Var}_{\text{Fam}}\in[0.1,0.9]$, subject effect (i.e., variance attributed to repeated measurements) $\text{Var}_{\text{Sub}}\in[0.1,0.9]$, and noise $\text{Var}_{\text{Noise}}\in[0.1,0.9]$. | We created a grid of known sources of variances: fixed effects $\text{Var}_{\text{FFX}}\in\left\{ 0.1, 0.2, \ldots, 0.9 \right\}[0.1,0.9]$, family effect (i.e., variance attributed to common environment) $\text{Var}_{\text{Fam}}\in\left\{ 0.1, 0.2, \ldots, 0.9 \right\}[0.1,0.9]$, subject effect (i.e., variance attributed to repeated measurements) $\text{Var}_{\text{Sub}}\in\left\{ 0.1, 0.2, \ldots, 0.9 \right\}[0.1,0.9]$, and noise $\text{Var}_{\text{Noise}}\in\left\{ 0.1, 0.2, \ldots, 0.9 \right\}[0.1,0.9]$. [Page 12, lines 21-24] |

7. Simulation 1 is an unbalanced longitudinal design (5,000 subjects with up to five repeated measurements but only 20,000 total observations). However, the manuscript does not specify how observations are allocated across visits. Similarly, the procedure for assigning family membership and the resulting family size distribution are not mentioned. Please clarify these aspects of the data-generating mechanism.

Response: We apologize for this lack of clarity in the simulation description. All simulations had unbalanced longitudinal design, where we started with a complete dataset (i.e., all individuals had all five repeated measurements). Then, we assigned these individuals into families, ensuring that number of family members did not exceed five. Then, we randomly removed measurements to achieve a desired overall sample size (20,000), simulating missingness as well as ensuring differing number of individuals in each family. We have edited the manuscript text to reflect these details:

| Original Manuscript | Revised Manuscript |
| --- | --- |
| […] we simulated data for 5,000 unique individuals having up to five repeated measurements and up to five members within a family, with a total of 20,000 observations. | […] we simulated data for 5,000 unique individuals with five repeated measurements; these individuals were grouped in families such that a family had no more than five members; then, we randomly subset the data to result in a total of 20,000 observations, reflecting repeated and missing measurements, as well as differing number of individuals across families.[Page 13, lines 3-7] |

8. In Figure 2, the boxplots show small absolute differences in the average number of false positives between covariance models. The QQ plot suggests separation in the extreme tail (around p < 1e-4), but the boxplots of counts have limited resolution in that regime given only 1000 tests per iteration. Consider increasing the number of null tests per iteration and reporting false-positive counts at smaller alpha. Alternatively, providing a tail summary statistics such as extreme quantiles. This will better reflect GWAS-scale settings.

Response: In Figure 2, the Q-Q plots show $36\times1000\times100\times10=36,000,000$ *p*-values (36 simulations settings, 1000 iterations/repetitions, 100 *X* variables, 10 *y* variables); the boxplots in the panel b of the same show the average false positive rate at three different false positive threshold values $\alpha=0.05, 0.01, 0.001$. These box plots are an overall summary of the 36 million *p*-values – a detailed breakdown of these across all simulation settings is shown in **Supplementary Figure S9**, where each of the 36 Q-Q plots show the distribution of 1,000,000 *p*-values indicating that the unstructured covariance false positive rates are well-calibrated (which is not the case for compound symmetry). Since all the information about the false positive calibration is present in the Q-Q plots and because we do not expect these results to change by increasing the number of null tests per iteration, we have left simulation 2 unchanged. In addition, in response to Reviewer 2 comment 6, we have done further analyses including false positive comparisons (simulation 5). The Q-Q plots from simulation 5 (**Supplementary Figures S26-S35**) show $16\times36\times1000\times100\times10=576,000,000$ *p*-values (16 different combinations of sample sizes, 36 simulations settings, 1000 iterations/repetitions, 100 *X* variables, 10 *y* variables) which further show similar trends to the results from simulation 2.

9. The reported runtimes and carbon footprints are informative. Reproducibility will be enhanced by providing additional computational details, including hardware configuration (number of cores, memory), the exact number of parallel workers used, and whether runtime was dominated by model fitting or genotype I/O.

Response: For benchmarking, we performed all computations on a compute cluster with AMD EPYC 7702 processor with 64 CPU cores. The *slurm* jobs were specified with 64 CPUs per task and 5 GB memory per CPU (i.e., $64\times5=320$ GB memory overall). For parallel computing, the same hardware allocation was used with 32 parallel jobs and 2 threads per parallel task specified (note that the parallelization is implemented using MATLAB’s parallel computing toolbox). Note that since the benchmarking was against glmmTMB (which is not a GWAS software), this set of results does not have genotype I/O as an overhead.

| Original Manuscript | Revised Manuscript |
| --- | --- |
| We also performed the time measurement using parallel computing with 24 parallel jobs. | We also performed the time measurement using parallel computing with 32 parallel jobs and 2 threads per job. [Page 14, lines 31-32] |
| We ran the timing measurements on a compute cluster with AMD EPYC 7702 processor with 64 cores, with the job specifications of 64 CPUs per task and 5GB memory per CPU. | We ran the timing measurements on a compute cluster with two AMD EPYC 7702 64-core processors, with the job specification of 64 CPUs per task and 5GB memory per CPU. [Page 13, lines 31-33] |
| To accelerate computing, we split the genetic data into chunks of 5,000 SNPs, parallelly processing twenty chunks. | To accelerate computing, we split the genetic data into chunks of 5,000 SNPs, processing eighteen chunks in parallel, with each parallel worker having seven threads, run on a compute cluster with two AMD EPYC 7702 64-core processers; the job specification specified 128 CPUs per task and 30 GB RAM per task. [Page 22, lines 27-30] |
| Additionally, we also ran a standard longitudinal GWAS, without including $s\left( \text{age} \right)$ and $G_{\text{res}} ⨀ s\left( \text{age} \right)$ terms. | Additionally, we also ran a standard longitudinal GWAS, without including $s\left( \text{age} \right)$ and $G_{\text{res}} ⨀ s\left( \text{age} \right)$ terms (run on the same hardware with the same specification, but twenty parallel chunks with six threads each). [Page 22, lines 30-32] |

10. In real data application, the omnibus Wald tests identified substantially more genome-wide significant SNPs than the main-effect-only analysis. To clarify whether these represent genuinely new loci or additional LD-correlated variants within the same region, please report LD-clumped locus counts for each phenotype and summarize overlap between the two approaches.

Response: We thank the Reviewer for the suggestion. Using the GWAS summary statistics (omnibus Wald test *p*-values when modeling the time-varying SNP effect and the *p*-values when modeling just the main effect of the SNP), we have now performed functional mapping and annotation using the SNP2GENE module of FUMA (Watanabe *et al*., 2017). Among other outputs, FUMA also reports the number of independently significant SNPs as well as the number of genetic loci. Comparison between the reported number of independently significant SNPs and the number of genetic loci show that for the three traits (length, weight, and BMI), FEMA-Long identified several new loci and not just LD-correlated variants. We have now reported this in the manuscript:

We used the SNP2GENE module of FUMA (30) to examine the number of independently significant SNPs and the number of identified genetic loci. For length, FUMA identified 134 independent SNPs and 54 loci when allowing the SNP effect to vary over time, as compared to 126 independent SNPs and 49 loci when modeling only the longitudinal main effect of SNPs; for weight, FUMA identified 201 independent SNPs and 46 loci when allowing the SNP effect to vary over time, as compared to 45 independent SNPs and 23 loci when modeling only the longitudinal main effect of SNPs; for BMI, FUMA identified 242 independent SNPs and 59 loci when allowing the SNP effect to vary over time, as compared to 124 independent SNPs and 44 loci when modeling only the longitudinal main effect of SNPs. [Page 25, lines 2-10]

11. Although simulation results show good type-I error control, the manuscript can benefit from explicit calibration summaries in the real data, especially given that a stage-1 covariance estimate is reused across millions of SNP tests. Please report QQ plots and a genomic inflation factor for the omnibus Wald test and compare with the main-effect-only test.

Response: We have now reported genomic inflation factor ($\lambda_{\text{GC}}$) for each phenotype and the two different GWAS analyses (both the time-varying GWAS using Wald *p* values and the standard longitudinal GWAS without smooth effects of age or $\text{SNP}\boldsymbol{\odot}s\left( \text{age} \right)$ interaction. In addition, we have included the QQ plots in the supplementary materials.

To quantify genomic inflation factor ($\lambda_{\text{GC}})$, we used the Wald test $p$ values, converted them to chi-squared statistics (using $1-p$ values as input to the inverse of the chi-square cumulative distribution function with 1 degree of freedom); then, $\lambda_{\text{GC}}$ was calculated as:

$\begin{aligned} \lambda_{\text{GC}}=\frac{\text{median}\left( \chi_{\text{obs}}^{2} \right)}{\text{median}\left( \chi_{\text{exp}}^{2} \right)} \#\left( 27 \right) \end{aligned}$where $\chi_{\text{obs}}^{2}$ were the observed chi-squared statistics, while $\chi_{\text{exp}}^{2}$ were the expected chi-squared statistics under the null (this simplifies to using the chi-squared statistics for a value of 0.5, since the under the null, the $p$ values are uniformly distributed). We quantified $\lambda_{\text{GC}}$ for both the scenarios: when allowing SNP effects to vary as smooth function of age, and when performing a standard longitudinal GWAS without any interactions or smooth terms. [Page 23, lines 1-9]

| Original Manuscript | Revised Manuscript |
| --- | --- |
| The GWAS analysis, completed in ~16.88 hours, revealed 2,836, 4,367, and 6,707 SNPs showing a main effect or a time-dependent effect on length, weight, and BMI respectively at $\alpha=5\times{10}^{-8}$; in comparison, longitudinal GWAS (without including splines or interactions; analysis completed in ~3.13 hours) revealed 2,126, 660, and 2,797 significant SNPs for length, weight, and BMI respectively (**Figure 5a-c**). | The GWAS analysis, completed in ~16.88 hours, revealed 2,836, 4,367, and 6,707 SNPs showing a main effect or a time-dependent effect on length ($\lambda_{GC}=1.10$), weight ($\lambda_{GC}=0.91$), and BMI ($\lambda_{GC}=1.36$) respectively at $\alpha=5\times{10}^{-8}$; in comparison, longitudinal GWAS (without including splines or interactions; analysis completed in ~3.13 hours) revealed 2,126, 660, and 2,797 significant SNPs for length ($\lambda_{GC}=1.25$), weight ($\lambda_{GC}=1.13$), and BMI ($\lambda_{GC}=1.21$) respectively (**Figure 5a-c**). Here, the $\lambda_{GC}>1$ is in line with other recent large-scale GWAS analyses and likely reflects the high polygenicity of the traits (see **Supplementary Figures S43-S45** for Q-Q plots). [Page 24, lines 6-10; page 25, lines 1-2] |

The Q-Q plots from the supplementary material are shown below:

**Length:**

**Figure S43**: Q-Q plots for length from standard longitudinal GWAS without modeling smooth effects of age or interaction of SNPs with smooth functions of age (**left**); from omnibus test across the main effect of age and the interaction of SNPs with the smooth functions of age (**middle**); and both the sets of *p*-values overlaid (**right**).

**Weight:**

**Figure S44**: Q-Q plots for weight from standard longitudinal GWAS without modeling smooth effects of age or interaction of SNPs with smooth functions of age (**left**); from omnibus test across the main effect of age and the interaction of SNPs with the smooth functions of age (**middle**); and both the sets of *p*-values overlaid (**right**).

**BMI:**

**Figure S45**: Q-Q plots for BMI from standard longitudinal GWAS without modeling smooth effects of age or interaction of SNPs with smooth functions of age (**left**); from omnibus test across the main effect of age and the interaction of SNPs with the smooth functions of age (**middle**); and both the sets of *p*-values overlaid (**right**).

12. In Figure 5d, the curves represent age-dependent SNP effects, but it is unclear how these lines are constructed from the fitted model. In particular, the y-axis is labeled eta, yet eta is not defined elsewhere in the manuscript or introduced in the model notation. Please clarify how these trajectories are computed, explicitly define eta, and state the scale (whether centered or normalized) of the effect shown in the figure.

Response: We apologize for the lack of clarity; in Figure 5d, the curves represent the effect of the selected SNPs on length, weight, and BMI at different age values; in other words, this is the cross-sectional effect of the SNP on the phenotype at a given age (age-dependent genetic effect). The trajectories are computed as the sum of the age basis function specific estimated beta coefficients, weighted by the basis functions; this is defined in equation 23 in the manuscript:

The estimated cross-sectional effect of the genotype $\boldsymbol{g}$ on the outcome, at a specific time $\mathcal{t}_{0}$:

$$\begin{aligned} {\hat{\eta}\eta}_{\mathcal{t}_{0}}=\frac{dy}{dg}=\sum_{s=0}^{S} \hat{\gamma}_{s}f_{s}\left( \mathcal{t}_{0} \right) \#\left( 23 \right) \end{aligned}$$

The scale of the beta coefficients is the same as in any other GWAS setting – for every additional copy of the SNP, the beta is the change in the phenotype. In our GWAS analyses, we only standardized the phenotypes, but the covariates were in their original units.

## Minor comments:

1. There are repeated texts describing the features of the proposed algorithm in the Abstract, the last paragraph of the Introduction, and the Methods (Page 3, last paragraph). Consider reducing redundancy.

Response: In the manuscript, we have listed the novel features of FEMA-Long in three places: towards the end of the Introduction section, at the end of providing an overview of the algorithm, and at the beginning of the Discussion section. We think it is helpful to the readers to reiterate these novel features in easily accessible spots, and this repetition of the main features of FEMA-Long also caters to the readers who may not read all sections of the manuscript. We have generally revised the manuscript to overall reduce any other text redundancy.

2. Page 6, lines 19-20, equation 11, Omega_hat should be used instead of sigma_hat.

Response: We have corrected this in the revised text. [Page 7, line 24]

3. Page 9, lines 3-4. My understanding is that the residuals yhat-res,GLS_i,g from (18) are the residualized phenotype for (14), estimated using the residualized genotype. Please check.

Response: We apologize for the confusion. In equation 18:

$$\begin{aligned} \tilde{y}_{i,g}^{\text{res,GLS}}=\hat{y}_{i}^{\text{res,GLS}}-\hat{g}_{i}^{\text{res,GLS}}\hat{\gamma}_{g} \end{aligned}$$

the $\hat{y}_{i}^{\text{res,GLS}}$ term is the stage one residuals ($\hat{y}_{i}^{\text{res,GLS}}=y_{i}-\boldsymbol{X}_{i}\hat{\boldsymbol{\beta}}$) from equation 14 (i.e., residuals when only accounting for the covariates and not the genotype); the term $\hat{g}_{i}^{\text{res,GLS}}$ are the residualized genotype vector (residualized for the covariates $\boldsymbol{X}$, $\hat{g}_{i}^{\text{res,GLS}}=g_{i}-\boldsymbol{X}_{i}\hat{\boldsymbol{\zeta}}$) from equation 16; $\hat{\gamma}_{g}$ is the estimated SNP effect of $\hat{g}_{i}^{\text{res,GLS}}$ on $\hat{y}_{i}^{\text{res,GLS}}$; therefore, the $\hat{g}_{i}^{\text{res,GLS}}\hat{\gamma}_{g}$ term is the predicted value of the (residualized) phenotype while $\hat{y}_{i}^{\text{res,GLS}}$ is the observed (residualized) phenotype.

We believe that the confusion might have been caused by an erroneous cross-referencing of the equation in the text, which we have fixed in the revised manuscript:

| Original Manuscript | Revised Manuscript |
| --- | --- |
| In essence, the residuals ${\hat{\boldsymbol{y}}}^{\text{res,GLS}}$ from $\left( 16 \right)$ are the residualized phenotype for $\left( 17 \right)$ which are estimated using the residualized genotype ${\hat{\boldsymbol{g}}}^{\text{res,GLS}}$. | Therefore, , the residuals ${\hat{\boldsymbol{y}}}^{\text{res,GLS}}$ from $\left( 164 \right)$ are the residualized phenotype for $\left( 17 \right)$ which are estimated using the residualized genotype ${\hat{\boldsymbol{g}}}^{\text{res,GLS}}$ (from $\left( 16 \right))$. [Page 10, lines 25-26] |

4. In equations 21 and 22, use g_i instead of g.

Response: We have now corrected the notation in equations 21 and 22:

| Original Manuscript | Revised Manuscript |
| --- | --- |
| $\begin{aligned} y_{i}=X_{i}\beta+\sum_{s=0}^{S} \left( \beta_{s}+\gamma_{s}g \right)f_{s}\left( t \right) +\varepsilon_{i} \#\left( 21 \right) \end{aligned}$ | $\begin{aligned} y_{i}=\boldsymbol{X}_{i}\boldsymbol{\beta}+\sum_{s=0}^{S} \left( {\alpha\beta}_{s}+\gamma_{s}g_{i}g \right)f_{s}\left( t \right) +\varepsilon_{i} \#\left( 21 \right) \end{aligned}$  [Page 12, line 1] |
| $\begin{aligned} y_{i}=X_{i}\beta+\sum_{s=1}^{S} \beta_{s}f_{s}\left( t \right) +\gamma_{g}g+ \\ \sum_{s=1}^{S} \gamma_{s}gf_{s}\left( t \right)+\varepsilon_{i} \#\left( 22 \right) \end{aligned}$ | $\begin{aligned} y_{i}=\boldsymbol{X}_{i}\boldsymbol{\beta}+\sum_{s=1}^{S} {\alpha\beta}_{s}f_{s}\left( \boldsymbol{t} \right)+\gamma_{g}g_{i}g+ \\ \sum_{s=1}^{S} \gamma_{s}g_{i}gf_{s}\left( t \right)+\varepsilon_{i} \#\left( 22 \right) \end{aligned}$  [Page 12, line 4] |

# Reviewer 2

## Summary of the paper

This manuscript introduces FEMA-Long, a scalable linear mixed model (LMM) framework for large-scale longitudinal genetic analyses with repeated measurements per individual. A central methodological contribution is to model the residual covariance as a linear combination of positive semidefinite matrices capturing multiple dependence sources and to estimate the associated variance components via a moment-based (MoM) procedure. Importantly, FEMA-Long further extends the modeling flexibility by incorporating spline basis expansions to capture non-linear fixed effects over continuous time. This approach avoids iterative likelihood-based fitting and yields a highly computationally efficient pipeline that is particularly appealing for analyzing large longitudinal datasets.

## Questions for the authors

1. In Equation (3), the residual covariance is decomposed as a linear combination of known PSD matrices (e.g., ZFZ⊤F, ZAZ⊤A, ZSZ⊤S, and I). When would the parameters be identifiable? For example, if ZFZ⊤F = I, then σ2Fand σ2ε would not be identifiable. More discussion on the identifiability conditions for the identifiability of parameters would be helpful.

Response: In general, the identifiability of variance components in random-effects models requires that the covariance patterns generated by the different random effects are not collinear. This is the standard identifiability condition in linear mixed models and variance-component models. In the FEMA setting used in the original paper, the variance components correspond to shared family environment, subject-level repeated measures, genetic similarity, and independent noise. These components are identifiable under typical study designs provided that i) some families contain more than one individual, ii) some subjects have repeated measurements, and iii) genetic relatedness varies within families (i.e., not all siblings are genetically identical). Under these conditions the covariance structures generated by the different random effects are distinct, and the corresponding variance parameters are identifiable.

In case of unstructured covariance, in an $F$, $S$, and $E$ model, when estimating the variance within a visit the subject and error terms are collinear. In other words, the interpretation of the subject effect changes. Because the visit-specific variance is now estimated directly, the subject component no longer represents a single variance parameter shared across visits, and the covariance estimated per visit absorbs the measurement noise. Similarly, when estimating the covariance between a pair of visits, the error term becomes unidentifiable and is estimated jointly with the subject term. We have now added a paragraph clarifying this as well as added two supplementary figures illustrating when the parameters become unidentifiable:

Note that when estimating the variance components (i.e., the diagonal entries of the variance-covariance matrices), the subject and error terms are colinear, and therefore these variance parameters (${\hat{\text{Ω}}}_{S,vv}^{2}$ and $\hat{\Omega}_{\epsilon,vv}^{2}$) cannot be uniquely identified, and thus are estimated as one. Similarly, when estimating the covariance components (i.e., the off-diagonal entries of the variance-covariance matrices), the system of equations constructed from 11 makes the error term unidentifiable (see **Supplementary Figures S1** and **S2** for a demonstration of why these terms cannot be identified). [Page 7, lines 22-28]

Identifiability of random effects parameters: variance

**Figure S1**: Demonstration of random effects parameter identifiability for variance terms. a) consider a dataset with eight subjects across two families, each having two repeated measurements; b) dummy-coding families $Z_{F}$ and subjects$Z_{S}$, followed by creating the $Z_{F}Z_{F}^{\top}$, $Z_{S}Z_{S}^{\top}$ terms and the independent error term denoted by $I$; c) following equation 11 in the manuscript, create a system of equations to solve for visit 1: the terms $S$ and $E$ are colinear, and cannot be uniquely identified; therefore, these are estimated together. A similar set of equations can be constructed for visit 2.

Identifiability of random effects parameters: covariance

**Figure S2**: Demonstration of random effects parameter identifiability for covariance terms. a) consider a dataset with eight subjects across two families, each having two repeated measurements; b) dummy-coding families $Z_{F}$ and subjects$Z_{S}$, followed by creating the $Z_{F}Z_{F}^{\top}$, $Z_{S}Z_{S}^{\top}$ terms and the independent error term denoted by $I$; c) following equation 11 in the manuscript, create a system of equations to solve for visit 1 – visit 2: the term $E$ becomes unidentifiable in this case.

In the case where $Z_{F}=I$, (i.e., there is no family structure), an $F$, $S$, and $E$ model would simplify to a $S$, $E$ model (since $Z_{F}=Z_{S}$); in this case, only one covariance parameter would be estimated.

2. The manuscript notes that, when estimating covariances within each pair of visits, the subject and error variances cannot be uniquely identified and are therefore estimated jointly. Could the authors clarify the implications of this non-identifiability for the interpretation of corresponding variance components?

Response: When estimating separate covariances for each pair of visits, the model identifies the total within-subject covariance across visits, but the decomposition of this covariance into a time-invariant subject component and visit-specific measurement noise is no longer unique. The implication of this is that the estimated covariance matrix should be interpreted as describing the longitudinal stability of the phenotype within individuals, rather than as separate subject and noise variance components. The off-diagonal covariances between visits reflect the degree to which measurements remain stable within individuals over time, while diagonal elements capture the total variance at each visit, including both stable subject differences and visit-specific noise. If the covariance matrix were purely diagonal, this would indicate little longitudinal stability and variability dominated by visit-specific noise. Conversely, substantial off-diagonal covariance indicates that measurements are correlated across visits, consistent with persistent subject-level differences over time. We have added this clarification in the revised manuscript:

The implication of this is that the estimated subject covariance matrix should be interpreted as describing the longitudinal stability of the phenotype within individuals, rather than as separate subject and noise variance components: the off-diagonal covariances between visits reflect the degree to which measurements remain stable within individuals over time, while diagonal elements capture the total variance at each visit, including both stable subject differences and visit-specific noise. If the estimated subject covariance matrix were purely diagonal, this would indicate little longitudinal stability and variability dominated by visit-specific noise; conversely, substantial off-diagonal covariance would indicate that measurements are correlated across visits, consistent with persistent subject-level differences over time. [Page 6, lines 28-30; page 7, lines 1-6]

3. Could the authors further explain the source of the approximation "≈" in Equation (7) more explicitly? In particular, since ˆyOLS res = (I − PX)y, the residual covariance is generally (I − PX)V (I − PX) rather than V. Could the authors clarify that under what assumptions it is valid to approximate (I − PX)V (I − PX) by V in Equation (7)?

Response: Thank you for the opportunity to clarify this aspect. When estimating the fixed effects, we do not ignore the variance explained by the covariates, i.e., we do not approximate $\left( I-P_{X} \right)\hat{V}\left( I-P_{X} \right)^{\top}$ by $V$. This is because the covariates may explain substantial portion of the phenotypic variance. We account for this by estimating the total residual variance $\hat{\sigma}^{2}$ using the residuals from OLS regression (i.e., equation 6):

$$\begin{aligned} \hat{\sigma}^{2}=\frac{1}{\left( n-p \right)}\sum_{i=1}^{n} \left( \hat{y}_{i}^{\text{res,OLS}} \right)^{2}\#\left( 6 \right) \end{aligned}$$

Equation 7 follows from our random effects model (3) where the left-hand side is replaced with its asymptotic method of moments estimate:

$$\frac{\boldsymbol{V}_{i,i^{'}}}{\sigma^{2}}\approx E\frac{\left( \hat{y}_{i}^{\text{res,OLS}}\times\hat{y}_{i^{'}}^{\text{res,OLS}} \right)}{\hat{\sigma}^{2}}$$

4. In Equation (11), the unstructured covariance is estimated entrywise. However, there seems no constraint on the off-diagonal entries. From this perspective, would the estimated unstructured covariance be not non-negative definite? Do the authors apply any approach to guarantee validity and numerical stability for the solution?

Response: Yes, the Reviewer is right in spotting that for the off-diagonal entries, there is no non-negativity constraint applied, since these are covariances and do not have a zero lower bound (unlike the diagonal entries). After individually estimating all elements of the variance-covariance matrix, while computing the standard errors, we use a nearest positive semidefinite algorithm that ensures a positive semidefinite matrix. We have added this clarification in the text; please see our response to Reviewer 1 comment 3 for additional details and the revised text.

5. Are there scenarios where the local system in Equation (11) becomes ill conditioned due to limited or highly unbalanced numbers of observations or certain visit-pairs? For example, late-term visits often have high attrition. How does the implementation handle visit-pairs where the number of overlapping samples is insufficient to reliably solve the moment equations?

Response: We thank the reviewer for raising this point. FEMA-Long is designed for large samples, where we expect that even if some visits have high attrition, the overall sample sizes are large enough. Since the estimation of covariance parameters is based on pairs of visits, if the number of shared observations between a pair of time points becomes too low, we expect some instability in the estimation process. Currently, we do not have an implementation to handle visit pairs where the number of overlapping samples is small. One could think of this problem as a bias-variance trade-off: for small sample situations, we could enforce priors or regularize the estimation process (i.e., have higher bias) and/or enforce smoothness in the estimation process – however, incorporating these priors requires making an assumption about the underlying data which may be different across different application areas. This is an open question, which could form the basis of future work. We have remarked about this in the Discussion section of the manuscript:

Fifth, when performing the visit-wise estimation of the covariance pattern, for certain scenarios where the number of shared observations between the visits are too low, the method of moments estimation could be unstable. While FEMA and FEMA-Long are designed for large samples, we envision improving the stability of the covariance estimation by updating our method to perform a joint estimation of all parameters instead of visit-wise estimation; other possibilities include introducing a shrinkage or regularization on the estimation of the covariance parameters and/or enforcing smoothness during the estimation; additionally, we could interpolate the visit-wise estimated covariances, resulting in smoothly changing covariances, and further extending the scope of time-varying random effects. [Page 30, lines 10-19]

6. In Simulations 1–2, the data-generating design appears to be essentially fixed (e.g., Simulation 1 and Simulation 2 uses 5,000 unique individuals with up to 5 repeated measurements and 20,000 observations). Could the authors please add a sensitivity analysis that varies the sample size in Simulations 1–2 to assess how the statistical performance of the proposed estimation depends on sample size?

Response: Thank you for the suggestion of examining the statistical performance of FEMA-Long as a function of sample size. In response to this, your previous comment, and a related comment from Reviewer 1 (comment 4), we have now included two more simulations: simulation 4 explores the parameter recovery performance of FEMA-Long as a function of different overall sample size and the minimum number of shared sample size between pairs of visits; simulation 5 explores the false positive calibration of FEMA-Long as a function of differing number of observations and number of shared observations between pairs of visits.

From simulation 4, we observed that the estimates from FEMA-Long were robust to different sample sizes and number of shared observations between pairs of visits (**Supplementary Figures S10-S25** and **Supplementary Tables S10-S11**), as long as the sample size was reasonably large. For relatively smaller overall sample sizes, the RMSE between the ground truth and the estimates from FEMA-Long reduced as the number of shared observations increased while for larger overall sample sizes, the RMSE remained stable across increasing number of shared observations between pairs of visits; further, the RMSE between ground truth and estimates from FEMA-Long reduced as the overall sample size increased (**Supplementary Table S10**, reproduced below for reference):

**Table S10**: **Summary of differences between the ground truth and the estimated parameters from FEMA**. Mean and standard deviation (across 84 experimental conditions) of the average (across 50 repeats) root mean squared error (RMSE) between ground truth and estimated coefficients from FEMA for differing total number of observations and minimum number of observations between pairs of visits

| $\boldsymbol{n}_{\text{Obs}}$ | $\text{min}_{\text{numObs}}$ | RMSE beta: truth – FEMA | RMSE family: truth – FEMA | RMSE subject: truth – FEMA |
| --- | --- | --- | --- | --- |
| 12000 | 500 | 0.0150 ± 0.0025 | 0.0128 ± 0.0033 | 0.0152 ± 0.0042 |
| 12000 | 600 | 0.0145 ± 0.0025 | 0.0125 ± 0.0033 | 0.0148 ± 0.0042 |
| 12000 | 700 | 0.0143 ± 0.0024 | 0.0123 ± 0.0032 | 0.0145 ± 0.0040 |
| 12000 | 800 | 0.0140 ± 0.0023 | 0.0121 ± 0.0031 | 0.0143 ± 0.0040 |
| 15000 | 500 | 0.0140 ± 0.0024 | 0.0111 ± 0.0030 | 0.0124 ± 0.0036 |
| 15000 | 600 | 0.0139 ± 0.0024 | 0.0110 ± 0.0029 | 0.0122 ± 0.0036 |
| 15000 | 700 | 0.0138 ± 0.0022 | 0.0109 ± 0.0030 | 0.0120 ± 0.0035 |
| 15000 | 800 | 0.0133 ± 0.0021 | 0.0108 ± 0.0030 | 0.0118 ± 0.0034 |
| 18000 | 500 | 0.0124 ± 0.0020 | 0.0094 ± 0.0026 | 0.0096 ± 0.0030 |
| 18000 | 600 | 0.0124 ± 0.0021 | 0.0094 ± 0.0027 | 0.0096 ± 0.0029 |
| 18000 | 700 | 0.0124 ± 0.0020 | 0.0094 ± 0.0026 | 0.0096 ± 0.0030 |
| 18000 | 800 | 0.0123 ± 0.0021 | 0.0094 ± 0.0027 | 0.0095 ± 0.0030 |
| 20000 | 500 | 0.0111 ± 0.0018 | 0.0085 ± 0.0026 | 0.0082 ± 0.0027 |
| 20000 | 600 | 0.0111 ± 0.0017 | 0.0085 ± 0.0026 | 0.0082 ± 0.0027 |
| 20000 | 700 | 0.0111 ± 0.0018 | 0.0086 ± 0.0025 | 0.0082 ± 0.0027 |
| 20000 | 800 | 0.0111 ± 0.0018 | 0.0085 ± 0.0025 | 0.0082 ± 0.0027 |

We observed a similar trend when we examined the RMSE between the estimates from glmmTMB toolbox and FEMA-Long (**Supplementary Table S11**, reproduced below for your reference):

**Table S11**: **Summary of differences between the estimated parameters from glmmTMB and the estimated parameters from FEMA**. Mean and standard deviation (across 84 experimental conditions) of the average (across 50 repeats) root mean squared error (RMSE) between ground truth and estimated coefficients from FEMA for differing total number of observations and minimum number of observations between pairs of visits

| $\boldsymbol{n}_{\text{Obs}}$ | $\text{min}_{\text{numObs}}$ | RMSE beta: truth – FEMA | RMSE family: truth – FEMA | RMSE subject: truth – FEMA |
| --- | --- | --- | --- | --- |
| 12000 | 500 | 0.0006 ± 0.0002 | 0.0053 ± 0.0021 | 0.0064 ± 0.0019 |
| 12000 | 600 | 0.0005 ± 0.0002 | 0.0050 ± 0.0021 | 0.0061 ± 0.0019 |
| 12000 | 700 | 0.0005 ± 0.0002 | 0.0050 ± 0.0021 | 0.0060 ± 0.0018 |
| 12000 | 800 | 0.0004 ± 0.0002 | 0.0048 ± 0.0020 | 0.0058 ± 0.0018 |
| 15000 | 500 | 0.0005 ± 0.0002 | 0.0043 ± 0.0018 | 0.0049 ± 0.0014 |
| 15000 | 600 | 0.0004 ± 0.0002 | 0.0042 ± 0.0017 | 0.0048 ± 0.0014 |
| 15000 | 700 | 0.0004 ± 0.0001 | 0.0041 ± 0.0018 | 0.0047 ± 0.0014 |
| 15000 | 800 | 0.0004 ± 0.0001 | 0.0040 ± 0.0017 | 0.0046 ± 0.0013 |
| 18000 | 500 | 0.0003 ± 0.0001 | 0.0030 ± 0.0013 | 0.0032 ± 0.0009 |
| 18000 | 600 | 0.0003 ± 0.0001 | 0.0030 ± 0.0013 | 0.0033 ± 0.0009 |
| 18000 | 700 | 0.0003 ± 0.0001 | 0.0030 ± 0.0013 | 0.0033 ± 0.0009 |
| 18000 | 800 | 0.0003 ± 0.0001 | 0.0030 ± 0.0013 | 0.0032 ± 0.0009 |
| 20000 | 500 | 0.0002 ± 0.0001 | 0.0022 ± 0.0009 | 0.0023 ± 0.0006 |
| 20000 | 600 | 0.0002 ± 0.0001 | 0.0022 ± 0.0010 | 0.0023 ± 0.0006 |
| 20000 | 700 | 0.0002 ± 0.0001 | 0.0022 ± 0.0010 | 0.0023 ± 0.0006 |
| 20000 | 800 | 0.0002 ± 0.0001 | 0.0022 ± 0.0010 | 0.0024 ± 0.0007 |

The results from simulation 5 (**Supplementary Figures S26-S35**) show that generally, FEMA-Long false positives are well-calibrated across a range of differing number of observations and number of shared observations between pairs of visits. There were two simulation settings $\text{Var}_{\text{Fam}}=0.7, \text{Var}_{\text{Sub}}=0.2, \text{Var}_{\text{Noise}}=0.1$ and $\text{Var}_{\text{Fam}}=0.8, \text{Var}_{\text{Sub}}=0.1, \text{Var}_{\text{Noise}}=0.1$, where we observed that there were a few combinations of $n_{\text{Obs}}$ and $\text{min}_{\text{numObs}}$, the distribution of the unstructured covariance $p$-values closely followed the null distribution but deviated away from the null towards the tail of the distribution; on examining this further, this was attributable to a few iterations where, by chance, the simulated data had extreme imbalance in the last visit (**Supplementary Figure S36**, reproduced below for your reference), likely leading to unstable estimation of the covariance parameters. As the sample size increased, the false positive calibration for the unstructured covariance improved; in contrast, the compound symmetry covariance pattern resulted in inflated Q-Q plots.

**Figure S36**: **Three examples where the** $\boldsymbol{p}$**-values for unstructured covariance were not well calibrated**. In these three examples, by chance, the simulation setting created a situation where the last visit had limited sample size; therefore, when estimating the covariance for the last pair of visits, the overlapping sample size would be close to (or equal to) $\text{min}_{\text{numObs}}$, likely leading to unstable covariance estimation, leading to inflated $p$-values.

# Reviewer 3

The authors introduced FEMA-Long to model unstructured covariances for discovery of time-dependent effects in longitudinal datasets. They demonstrated the effectiveness and efficiency of FEMA-Long through extensive simulations. They also applied FEMA-Long to several GWAS datasets, highlighting the importance of modeling the time-dependent effects and revealing how the SNP effects change over time. Overall, the paper is well written, and the experimental results are solid. I have some comments as follows.

1. Nomenclature: fixed effects refer to the coefficients ($\beta$) of covariates ($X$), rather than the covariates themselves. Please correct this throughout the manuscript.

Response: Thank you for pointing this out; we have now corrected this throughout the manuscript: the term fixed effects is now used to refer to the beta coefficients, while we have used the term *covariates* when referring to the *X* variables.

2. Page 4 Line 9: $X_i$ should be a number if $p = 1$ and a vector if $p > 1$.

Response: We have corrected this in the revised manuscript:

| Original Manuscript | Revised Manuscript |
| --- | --- |
| […] $X_{i}$ are the values of the fixed effects for observation $i$ ($X_{i}$ is a vector if $p=1$ and a matrix if $p>1$). | […] $\boldsymbol{X}_{i}$ are the values of the covariates for observation $i$ ($X_{i}$ is a scalar if $p=1$ and a vector if $p>1$) [Page 5, lines 32-33] |

3. In the GWAS analysis, the dynamic patterns of the variance components are interesting. However, current findings remain largely descriptive and lack rigorous statistical validation. It would significantly strengthen the conclusions by performing formal statistical tests for the dynamic changes of the variance components.

Response: The estimated covariance components across the random effects and across visits are estimated using method of moments, which – despite having asymptotic consistency with maximum likelihood (ML)/restricted maximum likelihood (REML) estimates – does not directly support statistical inference.

A maximum likelihood implementation in FEMA could be used to enable: a) testing for the presence of random effects (i.e., testing against the null hypothesis that the random effects are zero), and b) comparing random effects against each other (or more specifically, comparing the variance and covariance parameters across time). For the first scenario, the general recommendation is to use a likelihood ratio test: compare the (log) likelihood for a model with and without including random effects. In the original version of FEMA, we do have a maximum likelihood implementation, which is straightforward as all parameters have a lower bound of zero; then, a profile likelihood can be used to compute the confidence interval. In the FEMA-Long framework, with unstructured covariance, the implementation of ML is much more involved because of the presence of both bounded parameters (variances) and unbounded parameters (covariances); this also makes the profile likelihood harder to compute. By extension, when comparing variance-covariance parameters across visits (entries of the unstructured covariance matrices), similar profile likelihood-based confidence intervals would be required to compare the parameters. Similarly, using the Fisher information matrix approach is not straightforward as it requires ML/REML estimates and then using a normal distribution approximation of the estimates to get a confidence interval. This may be a future possibility with the implementation of the joint estimation of the variance-covariance parameters using (log) Cholesky decomposition but is beyond the scope of the current manuscript.

While we currently do not have a computationally efficient way of effectively computing the confidence intervals on the covariance parameters for drawing inferences about the statistical differences either against zero or between the parameters themselves, FEMA and FEMA-Long support wild bootstrap resampling which could be used for estimating a distribution of the estimated parameters. In the current manuscript our goal was to perform inference on the fixed effects (particularly the SNPs); implementation of statistical inference on unstructured random effects is a potential future direction of our work, which we have remarked upon in the Discussion section:

Future work will explore these and incorporate additional features like binning the phenotypes, computation of age-specific risk scores, extension to non-continuous phenotypes such as Bernoulli distribution, allowing statistical inference on the random effects estimates, using longitudinal trajectories for prediction, and the application of FEMA-Long to longitudinal complex trait phenotypes such as mental health. [Page 30, lines 20-24]

4. When scaling FEMA for GWAS-like analyses, do you perform two separate statistical tests for each SNP—one to determine if the main effect is non-zero, and another to assess the presence of time-dependent effects? Please describe it clearly in the method section.

Response: Performing the omnibus Wald test (equation 13: $W=\left( \boldsymbol{L}\hat{\boldsymbol{\beta}} \right)^{\top}\left\{ \boldsymbol{L}\hat{\text{Cov}}\left( \hat{\boldsymbol{\beta}} \right)\boldsymbol{L}^{\top} \right\}^{-1}\left( \boldsymbol{L}\hat{\boldsymbol{\beta}} \right)$), computes a single hypothesis test across the main effect and the time-dependent effects for every SNP. However, a user may further perform statistical inference individually on the main effect and on the time-dependent effect; in that case, they would need to perform a multiple comparison correction (i.e., change their GWAS significance threshold). Alternatively, a user may just wish to test the null hypothesis that there is no time-dependent effect. We have added a clarifying note to this extent in the revised manuscript:

Having estimated the coefficients, we can use equation $(13)$ to either perform an omnibus test across the main and the time-dependent genetic effect, or perform an omnibus test for just the time-dependent effect and a separate test for just the main effect of the SNP (if desired). Depending on the number of tests performed, multiple comparison correction may be necessary. [Page 11, lines 26-29]

Please note that the Miami plots from GWAS analyses (Figures 5-7) depict two independent GWASes – the top panel is the GWAS version with smooth effects of age as well as time-varying genetic effects, and the lower panel is the GWAS version without smooth effects of age and without any time-varying genetic effect (i.e., standard longitudinal GWAS): we performed these two analyses to showcase the boost in discovery when allowing SNP effects to be time-varying.

5. Can your method be extended to handle binary traits? In GWAS, many clinical outcomes and diseases are recorded as binary rather than continuous.

Response: FEMA and FEMA-Long are designed for continuous phenotypes with approximately normally distributed errors. However, as the Reviewer rightly points out, there is an interest in using mixed-effects algorithms for estimating of the effect of genetic variants on binary phenotypes. At the moment, we do not have an implementation that extends FEMA/FEMA-Long to a binary phenotype. This is a non-trivial extension of the scope of FEMA and is one of the future directions of our work. We have mentioned this at the end of the Discussion section as a future direction:

| Original Manuscript | Revised Manuscript |
| --- | --- |
| Future work will […] computation of age-specific risk scores, and the use of […] | Future work will […], computation of age-specific risk scores, extension to non-continuous phenotypes such as Bernoulli distribution, allowing statistical inference on the random effects estimates, using longitudinal trajectories […]. [Page 30, lines 20-23] |

6. One notable advantage of FEMA-Long is its computational efficiency, as mentioned in the manuscript. However, this point has not been sufficiently demonstrated. Please include more competing methods for a more comprehensive comparison and highlight the novelty of your algorithm.

Response: Thank you for the suggestion; we have now additionally performed a timing (and carbon footprint) comparison of FEMA-Long with *lme4* package in *R* (using the *lmer* function). We are not aware of any other widely-used method that would allow modeling of multiple unstructured covariances within a random effects framework; hence we have limited the comparison to two well-known toolboxes in *R*. We have updated Figure 3 to include the lmer comparison as well as included the carbon footprints; please see Supplementary Tables S5, S6, and S9 for additional details on the comparison with lmer and Supplementary Tables S1-S4, and S7-S8 for additional details on the comparison with glmmTMB; updated **Figure 3** is included below for your reference:

**Figure 3: Time taken and carbon footprint for fitting linear mixed-effects models with unstructured covariances.** In each panel, the line(s) indicate the time taken (in seconds) on the left *y*-axis and the carbon footprint (in grams of carbon dioxide emission) on the right *y*-axis for fitting linear mixed-effects models with unstructured covariances for family and subject random effects and 50 covariates across a range of number of observations (shown on the *x*-axis). Panel **a)** shows the time taken by and the carbon footprint for FEMA (green), glmmTMB (orange), and lme4 (from the lme4 toolbox; purple) for a single outcome variable across a range of number of observations. Panel **b)** shows the time taken by and the carbon footprint of FEMA with serial computing, and panel **c)** shows the time taken by and the carbon footprint of FEMA with parallel computing (32 parallel processes) across a range of increasing number of outcome variables, indicated by the color of the line. In all the panels, both axes are log-spaced and the *y*-axes tick labels are rounded off.
