# Supplementary material for: FEMA-Long: Modeling unstructured covariances for discovery of time-dependent effects in large-scale longitudinal datasets
Source: PLoS Genet. 2026 Jun 11;22(6):e1012184. doi: 10.1371/journal.pgen.1012184 (PMC13286282; doi:10.1371/journal.pgen.1012184)
Supplement: Attachment — Submitted filename: FEMA-Long_R2-ResponseLetter.docx [file pgen.1012184.s068.docx]

Response to Reviewer’s Comments: R2

We thank the Reviewers for their careful review of our revised manuscript, and the Editors for giving us this opportunity to provide clarification on the few outstanding comments. Below, we provide a point-by-point response to each of the comments raised by Reviewer 2. The comments by the Reviewer are in fixed-width blue text while our responses are in regular serif font in black. Additionally, when possible, we provide a comparison between the old and new manuscript with the changed content highlighted; the corresponding page and line numbers reflect the location of the change in the tracked changes version of the revised manuscript.

I thank the authors for the substantial revision and the detailed response. The revision has addressed my major concerns. I only have a few comments remaining.

1. After re-reading Equations (3)–(8), I still have some questions about the approximation underlying Equation (7). If i ̸= i′, i.e., for off-diagonal entries, it seems that the $\sigma_{\varepsilon}^{2}$ term should not appear on the right-hand side, since the noise component is proportional to the identity matrix (please correct me if I am misunderstanding this point).

More generally, it is still unclear to me how the normalization factor $\hat{\sigma}^{2}$ should be interpreted relative to $\sigma^{2}=\text{tr}(V)/n$ since the covariance matrix of OLS residuals seems to be $(I-P_{X})V(I-P_{X})$, where $P_{X}=X\left( X^{\top}X \right)^{-1}X^{\top}$ (again please correct me if I am misunderstanding this point). Relatedly, one might expect that $E\left( \hat{\sigma}^{2} \right)=\frac{1}{n-p}\text{tr}\left[ (I-P_{X})V(I-P_{X}) \right]$, which is associated with a projected residual-scale quantity rather than directly with $\text{tr}(V)/n$.

I also wonder whether this step could be clarified by writing the expectation explicitly on the residual product in the numerator, with normalization by $\sigma^{2}$ or the estimator $\hat{\sigma}^{2}$ applied afterward. Additional technical details would make this part of the model much easier to follow.

Response: Thank you for raising this point. Our previous notation was not sufficiently explicit, leading to this ambiguity. The method-of-moments estimator is fit using the full system of equations across all observation pairs $\left( i,i^{'} \right)$; therefore, $i=i^{'}$ pairs contribute to the estimation of the residual error variance while $i\neq i^{'}$ pairs do not. In the revised manuscript, we have updated equation 7 to clarify that the corresponding entries from the block-diagonal random effects design matrices for the error term would be zero, when $i\neq i^{'}$:

$$\begin{aligned} E\frac{\left( \hat{y}_{i}^{\text{res,OLS}}\times\hat{y}_{i^{'}}^{\text{res,OLS}} \right)}{\hat{\sigma}^{2}}\approx\sigma_{F}^{2}\left( \left( Z_{F}Z_{F}^{\top} \right)_{i,i^{'}\left\{ i,i^{'} \right\}} \right)+\sigma_{A}^{2}\left( \left( Z_{A}Z_{A}^{\top} \right)_{i,i^{'}\left\{ i,i^{'} \right\}} \right)+\sigma_{S}^{2}\left( \left( Z_{S}Z_{S}^{\top} \right)_{i,i^{'}\left\{ i,i^{'} \right\}} \right)+\sigma_{\epsilon}^{2}I_{i,i^{'}} \#\left( 7 \right) \end{aligned}$$

We agree with the Reviewer regarding the subtle difference between the covariance of the OLS residuals, $\text{Cov}\left( \hat{e} \right)=(I-P_{X})V(I-P_{X})$, and the covariance of the modeled error $V$. The residual products used in the method-of-moments estimation are quantities in the projected space $(I-P_{X})V(I-P_{X})$, and serve as approximations to the corresponding entries of $V$. In the homoskedastic case, $V=\sigma^{2}I$, $\mathbb{E}\left( \frac{\hat{e}^{\top}\hat{e}}{n-p} \right)=\sigma^{2}$; in the heteroskedastic case, the discrepancy will be governed by $\text{tr}\left( P_{X}V \right)$, since $\text{tr}\left[ \left( I-P_{X} \right)V\left( I-P_{X} \right) \right]=\text{tr}(V)-\text{tr}(P_{X}V)$. Generally, for large sample sizes $n$ (which is the use case for FEMA), and when $n\gg p$, the quantity $\text{tr}\left( P_{X}V \right)/(n-p)$ will shrink to a small number, which will not have any material affect on the estimation of the variance components. For example, we created a simple simulation (*n* = 5000, *p* = 50, random effects of family and subjects); $V=\sigma_{F}^{2}Z_{F}Z_{F}^{\top}+\sigma_{S}^{2}Z_{S}Z_{S}^{\top}+\sigma_{E}^{2}I$; we observed that ${\text{tr}\left[ \left( I-P_{X} \right)V\left( I-P_{X} \right) \right]}/{(n-p)}=0.9999$ and $\text{tr}(V)/n=1$, indicating a minor difference by omitting the $\text{tr}\left( P_{X}V \right)$ term.

2. The clarification that a nearest symmetric positive semidefinite correction is applied before computing standard errors is helpful. My remaining question is about its inferential impact. It is unclear whether this is usually a minor numerical adjustment or whether it can sometimes materially affect downstream inference. If feasible, it would be helpful to include a brief sensitivity analysis, for example reporting the size of the perturbation induced by the correction and whether it meaningfully changes standard errors, test statistics, or significance conclusions. Since the simulation setting provides access to the true covariance structure, it may also be informative to compare covariance estimation error before and after the nearest-PSD correction.

Response: Thank you for the suggestion. We performed a simulation where we generated covariance matrices with pre-specified minimum singular value and the condition number. Then, we computed the standard errors before and after using the *nearestSPD* algorithm. The results are reported in Supplementary Materials, including Supplementary Figure S52 (shown below). The results show that the largest absolute difference in the standard errors were minuscule (ranging between $5.55e^{-17}$ and $1.82e^{-12}$), indicating that the *nearestSPD* algorithm did not make any substantial differences to the covariance matrix. We note that this adjustment to covariance matrices to account for numerical issues is not uncommon (see, for example, (Higham, 1988; Ruppert et al., 2003, p. 337)). Additionally, the results of our simulation analyses of false positive rates under the null indicate that the use of *nearestSPD* still results in well-calibrated *p*-values.

**Figure S52**: Maximum absolute difference in standard errors across 50 fixed effects covariates as a function of minimum singular value and condition number of the matrix, before and after ensuring that the covariance matrix is positive semidefinite.

References:

Higham, N.J., 1988. Computing a nearest symmetric positive semidefinite matrix. Linear Algebra and its Applications 103, 103–118. https://doi.org/https://doi.org/10.1016/0024-3795(88)90223-6

Ruppert, D., Wand, M.P., Carroll, R.J., 2003. Semiparametric Regression, Cambridge Series in Statistical and Probabilistic Mathematics. Cambridge University Press.

3. The added simulations varying overall sample size and minimum overlap between visit pairs are useful. One point that may be worth stating more explicitly is what is meant here by “large sample.” In the present visit-wise covariance-estimation setting, the practically relevant notion if sample size seems to be the number of overlapping observations for each visit pair, rather than only the total number of observations. The revised simulations appear to support this interpretation, since both RMSE and calibration depend strongly on the minimum overlap across visit pairs. Making this point explicit would strengthen the practical guidance for readers.

Response: The method of moments estimator that FEMA and FEMA-Long relies on is asymptotically consistent. Practically, this makes it very hard to provide guidelines or a rule of thumb in terms of how large the sample size should be. As the Reviewer notes, it is not just the overall sample size but also the overlapping samples between pairs of visits. We have now added the following statement in the Discussion section to help clarify that both the factors are important considerations when using method of moments:

Fifth, when performing the visit-wise estimation of the covariance pattern, for certain scenarios where the number of shared observations between the visits are too low, the method of moments estimation could be unstable. Practically, this implies that both overall large sample size as well as the overlap between pairs of visits are important factors to consider, when evaluating the use of method of moments estimator. While FEMA and FEMA-Long are designed for large samples, we envision improving the stability of the covariance estimation by updating our method to perform a joint estimation of all parameters instead of visit-wise estimation […] [Page 29, lines 10-16]
