# Supplementary material for: FEMA-Long: Modeling unstructured covariances for discovery of time-dependent effects in large-scale longitudinal datasets
Source: PLoS Genet. 2026 Jun 11;22(6):e1012184. doi: 10.1371/journal.pgen.1012184 (PMC13286282; doi:10.1371/journal.pgen.1012184)
Supplement: Peer review history

# Peer review: Decision Letter 0

**DOI:** 10.1371/journal.pgen.1012184.r001

**Authors:** Lin S. Chen, Xiaofeng Zhu

29 Jan 2026

PGENETICS-D-25-01372

FEMA-Long: Modeling unstructured covariances for discovery of time-dependent effects in large-scale longitudinal datasets

PLOS Genetics

Dear Dr. Parekh,

Thank you for submitting your manuscript to PLOS Genetics. The editorial board and three reviewers have read the work. The reviewers provided some detailed suggestions.  We invite you to submit a revised version of the manuscript that addresses the points raised during the review process.

Please submit your revised manuscript by Mar 30 2026 11:59PM. If you will need more time than this to complete your revisions, please reply to this message or contact the journal office at plosgenetics@plos.org. When you're ready to submit your revision, log on to https://www.editorialmanager.com/pgenetics/ and select the 'Submissions Needing Revision' folder to locate your manuscript file.

Please include the following items when submitting your revised manuscript:

* A letter that responds to each point raised by the editor and reviewer(s). You should upload this letter as a separate file labeled 'Response to Reviewers'. This file does not need to include responses to any formatting updates and technical items listed in the 'Journal Requirements' section below.

* A marked-up copy of your manuscript that highlights changes made to the original version. You should upload this as a separate file labeled 'Revised Manuscript with Track Changes'.

* An unmarked version of your revised paper without tracked changes. You should upload this as a separate file labeled 'Manuscript'.

If you would like to make changes to your financial disclosure, competing interests statement, or data availability statement, please make these updates within the submission form at the time of resubmission. Guidelines for resubmitting your figure files are available below the reviewer comments at the end of this letter.

We look forward to receiving your revised manuscript.

Kind regards,

Lin S. Chen, Ph.D.

Academic Editor

PLOS Genetics

Xiaofeng Zhu

Section Editor

PLOS Genetics

Aimée Dudley

Editor-in-Chief

PLOS Genetics

Anne Goriely

Editor-in-Chief

PLOS Genetics

**Journal Requirements:**

If the reviewer comments include a recommendation to cite specific previously published works, please review and evaluate these publications to determine whether they are relevant and should be cited. There is no requirement to cite these works unless the editor has indicated otherwise.

1) Your manuscript is missing the following sections: Supplementary Information. Please ensure that your article adheres to the standard Methods article layout and order of Abstract, Author Summary, Introduction, Description of the Method, Verification and Comparison, Applications, Discussion, Acknowledgements, References, and Supplementary Information. For details on what each section should contain, see our Methods article guidelines:

https://journals.plos.org/plosgenetics/s/submission-guidelines#loc-manuscript-organization.

2) Please upload all main figures as separate Figure files in .tif or .eps format. For more information about how to convert and format your figure files please see our guidelines:

https://journals.plos.org/plosgenetics/s/figures

3) We have noticed that you have uploaded Supporting Information files, but you have not included a list of legends. Please add a full list of legends for your Supporting Information files after the references list.

4) Please amend your detailed Financial Disclosure statement. This is published with the article. It must therefore be completed in full sentences and contain the exact wording you wish to be published.

1) State what role the funders took in the study. If the funders had no role in your study, please state: "The funders had no role in study design, data collection and analysis, decision to publish, or preparation of the manuscript."

2) If any authors received a salary from any of your funders, please state which authors and which funders..

If you did not receive any funding for this study, please simply state: u201cThe authors received no specific funding for this work.u201d

5)  Please ensure that the funders and grant numbers match between the Financial Disclosure field and the Funding Information tab in your submission form. Note that the funders must be provided in the same order in both places as well.

**Reviewers' comments:**

Reviewer's Responses to Questions

**Comments to the Authors:**

**Please note here if the review is uploaded as an attachment.**

Reviewer #1: In this manuscript, the authors extended their previous FEMA method to allow unstructured longitudinal covariance, multiple random effects, spline interactions, and GWAS-scale testing. The proposed algorithm is computationally efficient and addresses an important gap in longitudinal GWAS methodology. I suggest the acceptance for publication after resolving the following issues:

Major comments:

1. Notations. I suggest using boldface consistently for vector and matrices to avoid confusion and avoid reusing the same symbols for different objects. For example, in equation 12, the coefficient of fixed effects in the first term is denoted by beta, which is a vector, and the coefficient of the effect on spline basis functions is by denoted beta_s, which is a scalar. This creates ambiguity, because beta_1 can be interpreted as either the effect of the first fixed effect or the first spline basis. In the following Omnibus Wald test (equation 13), beta is used again. But here it appears to refer only to coefficients for the spline basis functions, that is, beta_s in equation 12, because the degree of freedom is S.

2. In the GWAS-like scaling, Vhat is estimated from a genotype-free stage-1 model and then reused for genotype residualization and SNP testing (equations 9, 15, 17). The authors are implicitly assuming that omitting g when estimating variance components introduces negligible error in Vhat, and that uncertainty in Vhat is ignored in stage-2 inference. Please clarify this assumption explicitly. It would help to add a brief justification and a sensitivity check for a few large-effect loci showing that refitting Vhat does not change inference much.

3. The proposed algorithm estimates unstructured visit-by-visit covariance matrices using pairwise method-of-moments estimates (equation 11), with each covariance term estimated independently. This approach does not guarantee that the assembled covariance matrix is positive semidefinite. Since the GLS inference requires inversion of Vhat, please clarify whether any regularization, eigenvalue correction, or nearest positive-definite projection is applied in practice. If not, a short discussion of numerical stability and why this issue does not materially affect the simulations or GWAS results would be helpful.

4. Pairwise estimation uses only observations that have both visits v and v'. Real longitudinal cohorts usually have irregular visit attendance (later visits may have fewer subjects), leading to substantially different pairwise sample sizes. As a result, some Omega_vv' are estimated from many subjects and others from very few. This may lead to unstable inverse of V_hat in GLS. Please provide justification for this approach and consider a sensitivity check in simulations that explicitly reflects uneven visit overlap.

5. Please make it clear that the proposed method is designed for only continuous, approximately Gaussian outcomes, and does not apply to binary traits.

6. In simulation 1, the description "Var ∈ [0.1, 0.9]" is ambiguous and suggests a continuous interval. The authors appear to use a discrete grid with step size 0.1, which obtains in total 84 combinations. Please clarify that variance components take values in {0.1, 0.2, …, 0.9}, or otherwise explicitly describe the discretization.

7. Simulation 1 is an unbalanced longitudinal design (5,000 subjects with up to five repeated measurements but only 20,000 total observations). However, the manuscript does not specify how observations are allocated across visits. Similarly, the procedure for assigning family membership and the resulting family size distribution are not mentioned. Please clarify these aspects of the data-generating mechanism.

8. In Figure 2, the boxplots show small absolute differences in the average number of false positives between covariance models. The QQ plot suggests separation in the extreme tail (around p < 1e-4), but the boxplots of counts have limited resolution in that regime given only 1000 tests per iteration. Consider increasing the number of null tests per iteration and reporting false-positive counts at smaller alpha. Alternatively, providing a tail summary statistics such as extreme quantiles. This will better reflect GWAS-scale settings.

9. The reported runtimes and carbon footprints are informative. Reproducibility will be enhanced by providing additional computational details, including hardware configuration (number of cores, memory), the exact number of parallel workers used, and whether runtime was dominated by model fitting or genotype I/O.

10. In real data application, the omnibus Wald tests identified substantially more genome-wide significant SNPs than the main-effect-only analysis. To clarify whether these represent genuinely new loci or additional LD-correlated variants within the same region, please report LD-clumped locus counts for each phenotype and summarize overlap between the two approaches.

11. Although simulation results show good type-I error control, the manuscript can benefit from explicit calibration summaries in the real data, especially given that a stage-1 covariance estimate is reused across millions of SNP tests. Please report QQ plots and a genomic inflation factor for the omnibus Wald test and compare with the main-effect-only test.

12. In Figure 5d, the curves represent age-dependent SNP effects, but it is unclear how these lines are constructed from the fitted model. In particular, the y-axis is labeled eta, yet eta is not defined elsewhere in the manuscript or introduced in the model notation. Please clarify how these trajectories are computed, explicitly define eta, and state the scale (whether centered or normalized) of the effect shown in the figure.

Minor comments:

1. There are repeated texts describing the features of the proposed algorithm in the Abstract, the last paragraph of the Introduction, and the Methods (Page 3, last paragraph). Consider reducing redundancy.

2. Page 6, lines 19-20, equation 11, Omega_hat should be used instead of sigma_hat.

3. Page 9, lines 3-4. My understanding is that the residuals yhat-res,GLS_i,g from (18) are the residualized phenotype for (14), estimated using the residualized genotype. Please check.

4. In equations 21 and 22, use g_i instead of g.

Reviewer #2: The reviewer report has been uploaded as an attachment.

Reviewer #3: The authors introduced FEMA-Long to model unstructured covariances for discovery of time-dependent effects in longitudinal datasets. They demonstrated the effectiveness and efficiency of FEMA-Long through extensive simulations. They also applied FEMA-Long to several GWAS datasets, highlighting the importance of modeling the time-dependent effects and revealing how the SNP effects change over time. Overall, the paper is well written, and the experimental results are solid. I have some comments as follows.

1. Nomenclature: fixed effects refer to the coefficients ($\beta$) of covariates ($X$), rather than the covariates themselves. Please correct this throughout the manuscript.

2. Page 4 Line 9: $X_i$ should be a number if $p = 1$ and a vector if $p > 1$.

3. In the GWAS analysis, the dynamic patterns of the variance components are interesting. However, current findings remain largely descriptive and lack rigorous statistical validation. It would significantly strengthen the conclusions by performing formal statistical tests for the dynamic changes of the variance components.

4. When scaling FEMA for GWAS-like analyses, do you perform two separate statistical tests for each SNP—one to determine if the main effect is non-zero, and another to assess the presence of time-dependent effects? Please describe it clearly in the method section.

5. Can your method be extended to handle binary traits? In GWAS, many clinical outcomes and diseases are recorded as binary rather than continuous.

6. One notable advantage of FEMA-Long is its computational efficiency, as mentioned in the manuscript. However, this point has not been sufficiently demonstrated. Please include more competing methods for a more comprehensive comparison and highlight the novelty of your algorithm.

**********

**Have all data underlying the figures and results presented in the manuscript been provided?**

Large-scale datasets should be made available via a public repository as described in the *PLOS Genetics*       data availability policy, and numerical data that underlies graphs or summary statistics should be provided in spreadsheet form as supporting information.

Reviewer #1: None

Reviewer #2: None

Reviewer #3: None

**********

PLOS authors have the option to publish the peer review history of their article (what does this mean?). If published, this will include your full peer review and any attached files.

If you choose “no”, your identity will remain anonymous but your review may still be made public.

**Do you want your identity to be public for this peer review?**       For information about this choice, including consent withdrawal, please see our Privacy Policy.

Reviewer #1: No

Reviewer #2: No

Reviewer #3: No

[NOTE: If reviewer comments were submitted as an attachment file, they will be attached to this email and accessible via the submission site. Please log into your account, locate the manuscript record, and check for the action link "View Attachments". If this link does not appear, there are no attachment files.]

**Figure resubmission:**

While revising your submission, we strongly recommend that you use PLOS’s NAAS tool (https://ngplosjournals.pagemajik.ai/artanalysis) to test your figure files. NAAS can convert your figure files to the TIFF file type and meet basic requirements (such as print size, resolution), or provide you with a report on issues that do not meet our requirements and that NAAS cannot fix.

After uploading your figures to PLOS’s NAAS tool - https://ngplosjournals.pagemajik.ai/artanalysis, NAAS will process the files provided and display the results in the "Uploaded Files" section of the page as the processing is complete. If the uploaded figures meet our requirements (or NAAS is able to fix the files to meet our requirements), the figure will be marked as "fixed" above. If NAAS is unable to fix the files, a red "failed" label will appear above. When NAAS has confirmed that the figure files meet our requirements, please download the file via the download option, and include these NAAS processed figure files when submitting your revised manuscript.

**Reproducibility:**

To enhance the reproducibility of your results, we recommend that authors of applicable studies deposit laboratory protocols in protocols.io, where a protocol can be assigned its own identifier (DOI) such that it can be cited independently in the future. Additionally, PLOS ONE offers an option to publish peer-reviewed clinical study protocols. Read more information on sharing protocols at https://plos.org/protocols?utm_medium=editorial-email&utm_source=authorletters&utm_campaign=protocols

Attachment Submitted filename: Reviews.pdf

# Author comment: Author response to Decision Letter 1

**DOI:** 10.1371/journal.pgen.1012184.r002

25 Mar 2026

Attachment Submitted filename: FEMA-Long_R1-ResponseLetter.docx

# Peer review: Decision Letter 1

**DOI:** 10.1371/journal.pgen.1012184.r003

**Authors:** Lin S. Chen, Xiaofeng Zhu

15 Apr 2026

PGENETICS-D-25-01372R1

FEMA-Long: Modeling unstructured covariances for discovery of time-dependent effects in large-scale longitudinal datasets

PLOS Genetics

Dear Dr. Parekh,

Thank you for submitting your manuscript to PLOS Genetics. The three reviewers were largely satisfied with the revision, though a few questions and suggestions remain. We therefore invite you to submit a revised version of the manuscript that addresses the points.

Please submit your revised manuscript within by May 15 2026 11:59PM. If you will need more time than this to complete your revisions, please reply to this message or contact the journal office at plosgenetics@plos.org. When you're ready to submit your revision, log on to https://www.editorialmanager.com/pgenetics/ and select the 'Submissions Needing Revision' folder to locate your manuscript file.

Please include the following items when submitting your revised manuscript:

* A letter that responds to each point raised by the editor and reviewer(s). You should upload this letter as a separate file labeled 'Response to Reviewers'. This file does not need to include responses to formatting updates and technical items listed in the 'Journal Requirements' section below.

* A marked-up copy of your manuscript that highlights changes made to the original version. You should upload this as a separate file labeled 'Revised Manuscript with Track Changes'.

* An unmarked version of your revised paper without tracked changes. You should upload this as a separate file labeled 'Manuscript'.

If you would like to make changes to your financial disclosure, competing interests statement, or data availability statement, please make these updates within the submission form at the time of resubmission. Guidelines for resubmitting your figure files are available below the reviewer comments at the end of this letter.

We look forward to receiving your revised manuscript.

Kind regards,

Lin S. Chen, Ph.D.

Academic Editor

PLOS Genetics

Xiaofeng Zhu

Section Editor

PLOS Genetics

Aimée Dudley

Editor-in-Chief

PLOS Genetics

Anne Goriely

Editor-in-Chief

PLOS Genetics

**Reviewers' comments:**

Reviewer's Responses to Questions

**Comments to the Authors:**

**Please note here if the review is uploaded as an attachment.**

Reviewer #1: The authors have addressed all my comments. The manuscript has been significantly strengthened and I have no further major concerns.

Reviewer #2: The review is uploaded as an attachment.

Reviewer #3: The authors have addressed all of my concerns.

**********

**Have all data underlying the figures and results presented in the manuscript been provided?**

Large-scale datasets should be made available via a public repository as described in the *PLOS Genetics*       data availability policy, and numerical data that underlies graphs or summary statistics should be provided in spreadsheet form as supporting information.

Reviewer #1: None

Reviewer #2: None

Reviewer #3: None

**********

PLOS authors have the option to publish the peer review history of their article (what does this mean?). If published, this will include your full peer review and any attached files.

If you choose “no”, your identity will remain anonymous but your review may still be made public.

**Do you want your identity to be public for this peer review?**       For information about this choice, including consent withdrawal, please see our Privacy Policy.

Reviewer #1: No

Reviewer #2: No

Reviewer #3: No

[NOTE: If reviewer comments were submitted as an attachment file, they will be attached to this email and accessible via the submission site. Please log into your account, locate the manuscript record, and check for the action link "View Attachments". If this link does not appear, there are no attachment files.]

**Figure resubmission:**

While revising your submission, we strongly recommend that you use PLOS’s NAAS tool (https://ngplosjournals.pagemajik.ai/artanalysis) to test your figure files. NAAS can convert your figure files to the TIFF file type and meet basic requirements (such as print size, resolution), or provide you with a report on issues that do not meet our requirements and that NAAS cannot fix.

After uploading your figures to PLOS’s NAAS tool - https://ngplosjournals.pagemajik.ai/artanalysis, NAAS will process the files provided and display the results in the "Uploaded Files" section of the page as the processing is complete. If the uploaded figures meet our requirements (or NAAS is able to fix the files to meet our requirements), the figure will be marked as "fixed" above. If NAAS is unable to fix the files, a red "failed" label will appear above. When NAAS has confirmed that the figure files meet our requirements, please download the file via the download option, and include these NAAS processed figure files when submitting your revised manuscript.

**Reproducibility:**

To enhance the reproducibility of your results, we recommend that authors deposit laboratory protocols in protocols.io, where a protocol can be assigned its own identifier (DOI) such that it can be cited independently in the future. Additionally, PLOS ONE offers an option to publish peer-reviewed clinical study protocols. Read more information on sharing protocols at https://plos.org/protocols?utm_medium=editorial-email&utm_source=authorletters&utm_campaign=protocols

Attachment Submitted filename: Review_R2.pdf

# Author comment: Author response to Decision Letter 2

**DOI:** 10.1371/journal.pgen.1012184.r004

15 May 2026

Attachment Submitted filename: FEMA-Long_R2-ResponseLetter.docx

# Peer review: Decision Letter 2

**DOI:** 10.1371/journal.pgen.1012184.r005

**Authors:** Lin S. Chen, Xiaofeng Zhu

26 May 2026

Dear Dr Parekh,

We are pleased to inform you that your manuscript entitled "FEMA-Long: Modeling unstructured covariances for discovery of time-dependent effects in large-scale longitudinal datasets" has been editorially accepted for publication in PLOS Genetics. Congratulations!

Before your submission can be formally accepted and sent to production you will need to complete our formatting changes, which you will receive in a follow up email. Please be aware that it may take several days for you to receive this email; during this time no action is required by you. Please note: the accept date on your published article will reflect the date of this provisional acceptance, but your manuscript will not be scheduled for publication until the required changes have been made.

Once your paper is formally accepted, an uncorrected proof of your manuscript will be published online ahead of the final version, unless you’ve already opted out via the online submission form. If, for any reason, you do not want an earlier version of your manuscript published online or are unsure if you have already indicated as such, please let the journal staff know immediately at plosgenetics@plos.org.

In the meantime, please log into Editorial Manager at https://www.editorialmanager.com/pgenetics/, click the "Update My Information" link at the top of the page, and update your user information to ensure an efficient production and billing process. Note that PLOS requires an ORCID iD for all corresponding authors. Therefore, please ensure that you have an ORCID iD and that it is validated in Editorial Manager. To do this, go to ‘Update my Information’ (in the upper left-hand corner of the main menu), and click on the Fetch/Validate link next to the ORCID field.  This will take you to the ORCID site and allow you to create a new iD or authenticate a pre-existing iD in Editorial Manager.

If you have a press-related query, or would like to know about making your underlying data available (as you will be aware, this is required for publication), please see the end of this email. If your institution or institutions have a press office, please notify them about your upcoming article at this point, to enable them to help maximise its impact. Inform journal staff as soon as possible if you are preparing a press release for your article and need a publication date.

Thank you again for supporting open-access publishing; we are looking forward to publishing your work in PLOS Genetics!

Yours sincerely,

Lin S. Chen, Ph.D.

Academic Editor

PLOS Genetics

Xiaofeng Zhu

Section Editor

PLOS Genetics

Aimée Dudley

Editor-in-Chief

PLOS Genetics

Anne Goriely

Editor-in-Chief

PLOS Genetics

www.plosgenetics.org

BlueSky: @plos.bsky.social

----------------------------------------------------

Comments from the reviewers (if applicable):

Reviewer's Responses to Questions

**Comments to the Authors:**

**Please note here if the review is uploaded as an attachment.**

Reviewer #2: The authors have successfully addressed all of my concerns in the revised manuscript. I therefore recommend acceptance.

**********

**Have all data underlying the figures and results presented in the manuscript been provided?**

Large-scale datasets should be made available via a public repository as described in the *PLOS Genetics*       data availability policy, and numerical data that underlies graphs or summary statistics should be provided in spreadsheet form as supporting information.

Reviewer #2: None

**********

PLOS authors have the option to publish the peer review history of their article (what does this mean?). If published, this will include your full peer review and any attached files.

If you choose “no”, your identity will remain anonymous but your review may still be made public.

**Do you want your identity to be public for this peer review?**       For information about this choice, including consent withdrawal, please see our Privacy Policy.

Reviewer #2: No

----------------------------------------------------

**Data Deposition**

If you have submitted a Research Article or Front Matter that has associated data that are not suitable for deposition in a subject-specific public repository (such as GenBank or ArrayExpress), one way to make that data available is to deposit it in the Dryad Digital Repository. As you may recall, we ask all authors to agree to make data available; this is one way to achieve that. A full list of recommended repositories can be found on our website.

The following link will take you to the Dryad record for your article, so you won't have to re‐enter its bibliographic information, and can upload your files directly:

http://datadryad.org/submit?journalID=pgenetics&manu=PGENETICS-D-25-01372R2

More information about depositing data in Dryad is available at http://www.datadryad.org/depositing. If you experience any difficulties in submitting your data, please contact help@datadryad.org for support.

Additionally, please be aware that our data availability policy requires that all numerical data underlying display items are included with the submission, and you will need to provide this before we can formally accept your manuscript, if not already present.

----------------------------------------------------

**Press Queries**

If you or your institution will be preparing press materials for this manuscript, or if you need to know your paper's publication date for media purposes, please inform the journal staff as soon as possible so that your submission can be scheduled accordingly. Your manuscript will remain under a strict press embargo until the publication date and time. This means an early version of your manuscript will not be published ahead of your final version. PLOS Genetics may also choose to issue a press release for your article. If there's anything the journal should know or you'd like more information, please get in touch via plosgenetics@plos.org.

# Editor report: Acceptance letter

**DOI:** 10.1371/journal.pgen.1012184.r006

**Authors:** Lin S. Chen, Xiaofeng Zhu

PGENETICS-D-25-01372R2

FEMA-Long: Modeling unstructured covariances for discovery of time-dependent effects in large-scale longitudinal datasets

Dear Dr Parekh,

We are pleased to inform you that your manuscript entitled "FEMA-Long: Modeling unstructured covariances for discovery of time-dependent effects in large-scale longitudinal datasets" has been formally accepted for publication in PLOS Genetics! Your manuscript is now with our production department and you will be notified of the publication date in due course.

The corresponding author will soon be receiving a typeset proof for review, to ensure errors have not been introduced during production. Please review the PDF proof of your manuscript carefully, as this is the last chance to correct any errors. Please note that major changes, or those which affect the scientific understanding of the work, will likely cause delays to the publication date of your manuscript.

Soon after your final files are uploaded, unless you have opted out or your manuscript is a front-matter piece, the early version of your manuscript will be published online. The date of the early version will be your article's publication date. The final article will be published to the same URL, and all versions of the paper will be accessible to readers.

For Research Articles, you will receive an invoice from PLOS for your publication fee after your manuscript has reached the completed accept phase. If you receive an email requesting payment before acceptance or for any other service, this may be a phishing scheme. Learn how to identify phishing emails and protect your accounts at https://explore.plos.org/phishing.

Thank you again for supporting PLOS Genetics and open-access publishing. We are looking forward to publishing your work!

With kind regards,

Anita Estes

PLOS Genetics

On behalf of:

The PLOS Genetics Team

Carlyle House, Carlyle Road, Cambridge CB4 3DN | United Kingdom

plosgenetics@plos.org | +44 (0) 1223-442823

plosgenetics.org | Twitter: @PLOSGenetics
